# Aligned ELF and VCTE cut-offs facilitate risk staging in patients with metabolic dysfunction-associated steatohepatitis

**DOI:** 10.1016/j.jhepr.2026.102041

**Published:** 2026-09-18

**Authors:** Matthew F.W. Gee, Andrew N. Billin, Donald B. Chalfin, Rachel Fonstad, Edward B. De Vol, Jordan J. Feld, Keyur Patel, Arun J. Sanyal

**Affiliations:** 1 Institute of Medical Science, Temerty Faculty of Medicine, University of Toronto, Toronto, Ontario, Canada; 2 Siemens Healthcare Diagnostics Inc., Tarrytown, New York, United States; 3 Billin Life Sciences Consulting, LLC, San Mateo, California, United States; 4 University of California School of Medicine, San Francisco, California, United States; 5 Division of Gastroenterology, Department of Medicine, Toronto Centre for Liver Disease, Toronto General Hospital, University Health Network, Toronto, Ontario, Canada; 6 Stravitz-Sanyal Institute for Liver Disease and Metabolic Health, Division of Gastroenterology, Hepatology and Nutrition, Virginia Commonwealth University School of Medicine, Richmond, Virginia, United States

**Keywords:** Enhanced liver fibrosis (ELF) test, Fibrosis-4 (FIB-4), Steatotic liver disease (SLD), Metabolic dysfunction-associated steatotic liver disease (MASLD), Nonalcoholic fatty liver disease (NAFLD), Metabolic dysfunction-associated steatohepatitis (MASH), Nonalcoholic steatohepatitis (NASH), Immunoassay, Liver-related event (LRE), Liver stiffness measurement (LSM), Vibration-controlled transient elastography (VCTE)

## Abstract

**Background & Aims:**

Clinical practice guidance for non-invasive test (NIT)-based management of metabolic dysfunction-associated steatohepatitis (MASH) includes the enhanced liver fibrosis (ELF) test and vibration-controlled transient elastography (VCTE). However, recommended thresholds were derived independently and not calibrated to yield equivalent liver-related event (LRE) risk across tests. We aimed to identify ELF and VCTE cut-offs that are prognostically aligned and to evaluate whether agreement-based combinations at aligned thresholds improve near-term risk stratification.

**Methods:**

Individual-level data were pooled from patients across placebo-controlled trials of MASH and advanced fibrosis. Baseline ELF and VCTE were evaluated in relation to subsequent LREs and histological change at 1 year. Prognostic alignment was performed by identifying VCTE thresholds associated with observed LRE risks corresponding to pre-specified ELF cut-offs. The prognostic performance of individual tests and ELF/VCTE combinations was assessed using event risk, likelihood ratios, Kaplan-Meier analysis, and discrimination metrics (C-index and AUROC).

**Results:**

Risk-aligned pairings supported ELF 9.80/VCTE 12.0 kPa, ELF 10.50/VCTE 20.0 kPa and ELF 11.30/VCTE 30.0 kPa. Agreement-based ELF/VCTE stratification improved discrimination *vs*. VCTE alone (AUROC 0.779 *vs*. 0.705; *p =* 0.0129). At ELF 11.30/VCTE 30.0 kPa, LRE risk increased across negative concordance, discordance, and positive concordance strata (1.3%, 5.4%, and 21.6%, respectively). For prediction of histological change using ELF 9.80/VCTE 12.0 kPa, F3-to-F4 progression rates were 2.2%, 14.4%, and 25.5%, and F4 regression rates were 56.9%, 27.3%, and 12.2% across negative concordance, discordance, and positive concordance strata, respectively.

**Conclusions:**

ELF and VCTE thresholds can be aligned for comparable prognostic risk staging in advanced MASH. Agreement-based combinations of ELF and VCTE at aligned thresholds may help refine prediction of near-term LREs and histological change, supporting clinical risk assessment and informing MASH clinical trial enrichment strategies.

**Impact and implications:**

Current MASH care pathways increasingly rely on non-invasive tests, yet recommended enhanced liver fibrosis (ELF) and vibration-controlled transient elastography (VCTE) thresholds have largely been established independently. We found that ELF and VCTE thresholds can be aligned according to comparable risk and that concordant and discordant test results provide clinically meaningful stratification of both liver-related outcomes and likelihood of histological progression and regression. These findings may help support more consistent risk assessment and patient stratification across clinical practice and research settings.

## Highlights

• ELF and VCTE thresholds can be aligned to enable comparable risk staging.
• Stratification based on ELF/VCTE agreement predicts clinical outcomes and fibrosis trajectory.
• Concordantly high ELF/VCTE results identify patients most likely to progress.
• Concordantly low ELF/VCTE results identify patients most likely to regress.
• Discordant ELF/VCTE results identify an intermediate-risk patient population.

## Graphical abstract

**Figure:**
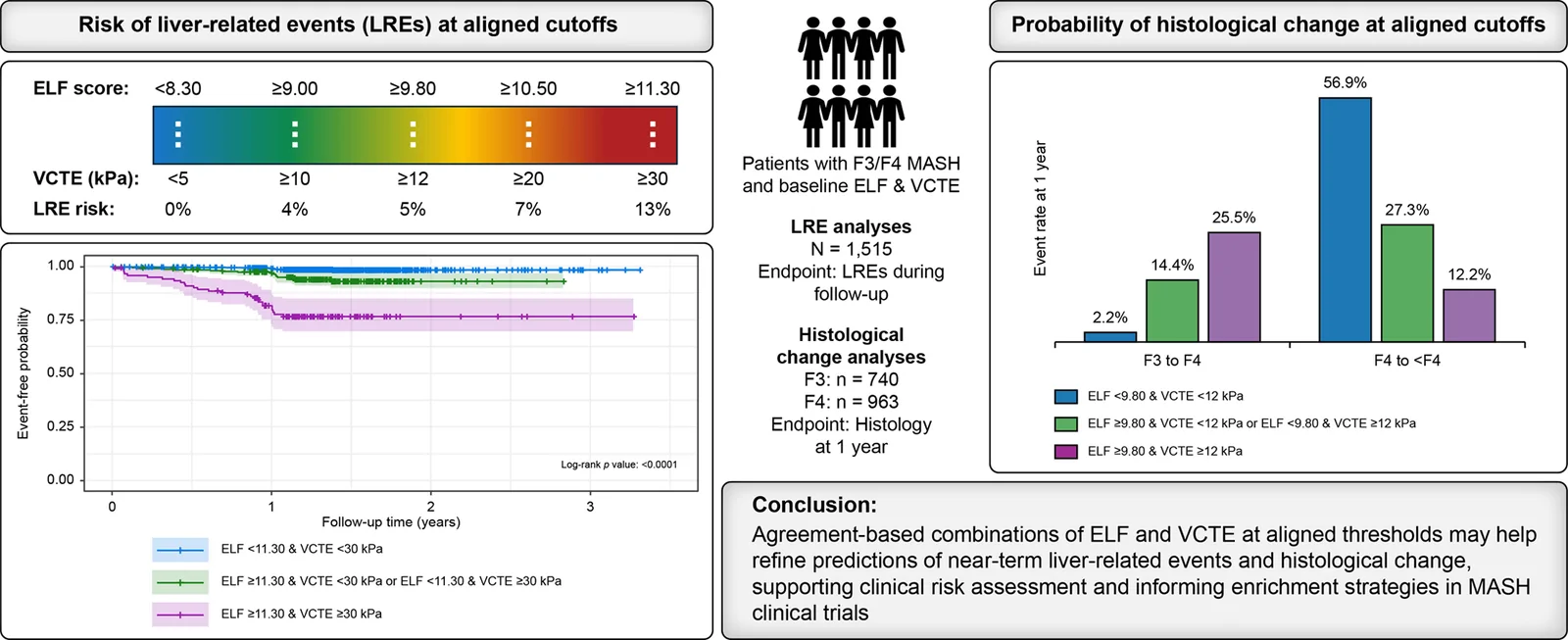

## Introduction

Metabolic dysfunction-associated steatotic liver disease (MASLD) is the most common chronic liver disease worldwide, with a global prevalence of up to 38% and a prevalence as high as 65% among individuals with type 2 diabetes mellitus.[1], [2], [3], [4], [5], [6] Metabolic dysfunction-associated steatohepatitis (MASH), characterized by hepatic steatosis, inflammation, and hepatocyte ballooning, is the more active form of the disease and a leading cause of cirrhosis complications, hepatocellular carcinoma, indication for transplantation, and liver-related death.^3^^,^^7^^,^^8^

Due to the invasive nature of liver biopsy and the inherent variability of histological findings, non-invasive tests (NITs), such as the enhanced liver fibrosis (ELF) test and liver stiffness measurement (LSM) by vibration-controlled transient elastography (VCTE), are recommended by clinical practice guidance from the American Association for the Study of Liver Diseases (AASLD) and the European Association for the Study of the Liver (EASL) as useful methods for detecting advanced fibrosis and assessing the risk of disease progression following first-line screening with Fibrosis-4 (FIB-4).^3^^,^^9^^,^^10^

The scientific literature delineates many different NIT cut-offs, with studies often deriving cut-offs *de novo* and optimizing them for their study population (*e.g*. quartiles, Youden index). This has led to non-standardized thresholds, such as ELF ≥9.50, often derived independently for either diagnostic or prognostic use.^11^ Although there is more consistency amongst the cut-off recommendations in clinical practice guidelines,^3^^,^^9^ the thresholds for different NITs were derived independently from one another.^12^^,^^13^ The lack of head-to-head NIT cut-off validation in the same population is problematic due to the heterogeneity across study populations and test thresholds, along with limited data on comparative studies, as was highlighted as a limitation in a recent AASLD systematic review of blood-based NITs for staging fibrosis in chronic liver disease.[14], [15], [16] Consequently, although many of the thresholds for ELF, VCTE, and FIB-4 recommended in clinical practice guidelines are supported by studies demonstrating good performance of individual NITs, whether thresholds across different NITs can yield equivalent diagnostic and prognostic performance for a given purpose has not been fully explored.

Studies have shown that ELF and VCTE prognosticate liver-related clinical outcomes, as would be expected due to their correlation with histology, a well-established predictor of clinical outcomes.[17], [18], [19], [20], [21], [22], [23], [24], [25], [26] However, the guideline-recommended thresholds for these NITs were primarily established based on their correlation with histological fibrosis stage rather than their prognostic performance.^12^^,^^13^ Rather than simply using NITs as a surrogate for histology, it is essential to establish test performance in terms of clinical outcome risk.^27^ Accordingly, a common dataset is necessary to permit test comparisons and to determine which thresholds across NIT modalities are equivalent with respect to prognostic performance. However, to the best of our knowledge, no studies have evaluated which ELF and VCTE thresholds are most aligned in relation to the risk of clinical outcomes.

To compare the ability of NITs to identify histological fibrosis stage and predict clinical events, we analyzed data from large placebo-controlled trials in patients with advanced fibrosis due to MASH. This pooled dataset includes NIT measurements, biopsy results and recorded liver-related clinical outcomes, thereby permitting direct performance comparisons between the NITs. In this study, we aimed to determine which ELF and VCTE cut-offs yield equivalent prognostic performance for liver-related clinical outcomes, and whether combining ELF and VCTE at aligned thresholds improves risk assessment compared with either test alone.

## Patients and methods

### Study population

This study pooled individual-level data from previously published, randomized, placebo-controlled phase II and III trials in participants with advanced fibrosis (F3 & F4) due to MASH, which evaluated selonsertib (STELLAR-3 [NCT03053050], STELLAR-4 [NCT03053063]), simtuzumab (GS-US-321-0105 [NCT01672866], GS-US-321-0106 [NCT01672879]), and belapectin (NASH-CX [NCT02462967]). An additional study was included for assessment of histological change at 1 year (ATLAS [NCT03449446]).

The NASH-CX study concluded after the planned follow-up period of 52 weeks, while the selonsertib and simtuzumab studies were discontinued after their interim analyses at 48 weeks and 96 weeks, respectively, after determining that these therapies were ineffective. Preliminary findings from these studies, including analyses of the ELF test, and detailed methods have been reported previously.[17], [18], [19]^,^^21^^,^^22^^,^[28], [29], [30], [31] As there were no meaningful differences between placebo and active treatment groups, all study arms were pooled for the analyses conducted in this study. Additionally, trial populations were pooled where appropriate to address study endpoints. A summary of relevant characteristics of the five clinical trials and the populations used for each analysis is presented in Table S1. The studies were approved by the institutional review boards or independent ethics committees at all participating sites and conducted in accordance with the Declarations of Helsinki and Istanbul. All participants provided informed consent.

### Study assessments

Participants included in the analysis had complete baseline measurements for VCTE and laboratory tests (including ELF) and had recorded follow-up for clinical outcomes (Fig. S1).

Liver fibrosis was staged at the time of enrollment (all studies), Week 48 (STELLAR-3, STELLAR-4, GS-US-321-0105, GS-US-321-0106, ATLAS), and Week 52 (NASH-CX). All biopsies were read by a single central reader (Dr. Zachary Goodman, Inova Health System) who was blinded to treatment assignment but not biopsy sequence. Histological assessments included, among other features, the adequacy of the biopsy specimen, confirmation of the diagnosis, fibrosis stage according to the NASH Clinical Research Network (NASH CRN) criteria, and modified Ishak fibrosis classifications.[17], [18], [19]^,^^21^^,^^22^^,^[28], [29], [30], [31]

The ELF test was performed using ADVIA Centaur XP systems (Siemens Healthcare Diagnostics Inc., Tarrytown, NY, USA), with the ELF score automatically calculated by the instrument. The ELF test is a non-invasive serum-based test that quantifies three extracellular matrix components reflecting liver fibrosis and fibrogenesis: hyaluronic acid, amino-terminal propeptide of type III procollagen, and tissue inhibitor of matrix metalloproteinase 1.[1], [2], [3] Results are combined in an algorithm, calculated automatically by the system, that yields a unitless ELF score.[4], [5], [6]

LSM by VCTE was performed according to manufacturer recommendations using FibroScan (Echosens, Paris, France) by trained operators, with participants in a fasting state and following standard reliability and probe selection criteria, as previously described.^22^^,^^32^ Fasting blood samples were used to calculate the FIB-4 score according to the published formula.^33^

Liver-related events (LREs) were adjudicated by a central committee of experts and defined as clinically apparent ascites requiring treatment, Grade ≥2 hepatic encephalopathy according to the West Haven criteria that required treatment, and portal hypertension-related gastrointestinal bleeding, liver transplantation, qualification for transplantation (model for end-stage liver disease ≥15), liver-related death, or hepatocellular carcinoma.[17], [18], [19]^,^^21^^,^^22^^,^[28], [29], [30], [31]

### Analyses and endpoints

The primary aims of the study were to 1) determine which established ELF and VCTE cut-offs yield equivalent prognostic performance for LREs and 2) evaluate whether agreement-based combinations of ELF and VCTE at aligned thresholds could further stratify near-term LRE risk and improve prognostic performance compared with either test alone. The utility of agreement-based combinations of ELF and VCTE at aligned thresholds to predict histological change at 1 year was evaluated as a contextual, secondary analysis.

### Statistical approaches

Observed risk of LREs was estimated as the proportion of participants with baseline NIT values at or above a given cut-off who experienced an event during follow-up. Exact binomial 95% CIs were calculated using the Clopper-Pearson method. Baseline ELF and VCTE measurements were categorized using thresholds described in clinical practice guidelines and prior studies.^3^^,^^9^^,^^22^^,^[34], [35], [36] Eight ELF cut-offs ranging from 7.70 to 12.80 and seven VCTE cut-offs ranging from 5.0 to 30.0 kPa were evaluated.

To align ELF and VCTE thresholds with respect to prognostic performance, event risks were calculated across the observed distributions of baseline ELF and VCTE values. For each ELF cut-off, the VCTE threshold yielding the closest observed LRE risk was identified by minimizing the absolute difference in event proportions above each threshold. This approach yielded risk-based alignments between ELF and VCTE cut-offs without assuming a linear or monotonic relationship between test values. To facilitate clinical interpretation, these empirically derived thresholds were mapped to the nearest established VCTE cut-offs used in prior studies and guidelines, and corresponding event risks were recalculated with exact binomial confidence intervals, as described in the Supplemental Materials. Inverse mapping was also performed to identify ELF cut-offs corresponding to established VCTE thresholds to verify the robustness of the risk-based alignment strategy.

Risk stratification according to ELF and VCTE, alone and in combination, and according to histologic fibrosis stage was further characterized using multiple complementary metrics, including absolute event rate, interval likelihood ratio, and incidence of LREs per 1,000 person-years of follow-up. Absolute event rate was calculated as the number of events divided by the number of participants within each stratum, with exact 95% CIs. Interval likelihood ratios were calculated as the ratio of the proportion of events occurring within a given stratum to the proportion of non-events occurring within that stratum. Time-to-event analyses were performed using Cox proportional hazards regression, with follow-up time expressed in years and LREs as the outcome. Kaplan-Meier methods were used to estimate event-free probability across the various risk strata.

For model-based discrimination analyses, Cox proportional hazards models were specified using categorical risk strata derived from single-test ELF and VCTE thresholds and from agreement-based combinations of aligned ELF and VCTE cut-offs. Single-test models were constructed using guideline-recommended thresholds for advanced fibrosis (ELF 9.80; VCTE 12.0 kPa) and cirrhosis rule-in (ELF 11.30; VCTE 20.0 kPa), which defined low-, intermediate-, and high-risk strata.^3^^,^^9^ These models were compared with an agreement-based combination model defined using aligned thresholds of ELF 11.30 and VCTE 30.0 kPa to evaluate relative discrimination for LREs. Model discrimination was assessed using Harrell’s concordance index (C-index). Model fit and relative performance were compared using log-likelihood, Akaike information criterion (AIC), and Bayesian information criterion (BIC), with lower AIC/BIC and higher log-likelihood indicating improved model fit.

Receiver-operating characteristic (ROC) analyses were used to further assess discrimination of agreement-based combination models relative to single-test models. These included logistic regression-based step-function ROC with comparison of area under the ROC curve (AUROC) using DeLong’s test, Cox regression-based ROC, and time-dependent ROC analyses.

All statistical analyses were performed using R version 4.4.2 (R Foundation for Statistical Computing, Vienna, Austria), including the *survival*, *survivalROC*, *timeROC*, *ggsurvfit*, and *ggplot2* packages. Two-sided *p* values <0.05 were considered statistically significant.

## Results

### Demographics, baseline characteristics and clinical outcomes

Full demographic details for the studies included in this analysis have previously been described.[17], [18], [19]^,^^21^^,^^22^^,^[28], [29], [30], [31] Summaries of the baseline demographics and clinical characteristics are presented in Table 1. Baseline mean and median VCTE measurements arranged by baseline ELF strata are presented in Table S2. The median (Q1, Q3) follow-up time in this dataset was 15.7 (13.5, 18.3) months. A total of 57 LREs were recorded during the follow-up period (Table S3).

**Table 1:** Baseline demographics and clinical characteristics.

Baseline parameter	Main dataset (progression to LREs)	Supplementary dataset (histological changes)
N	1,515	1,703
Female (%)	915 (60%)	1,041 (61%)
Type 2 diabetes present (%)	1,097 (72%)	1,237 (73%)
Age category (%)		
≤34	22 (1%)	20 (1%)
35-39	36 (2%)	37 (1%)
40-44	71 (5%)	73 (4%)
45-49	148 (10%)	154 (9%)
50-54	204 (13%)	222 (13%)
55-59	332 (22%)	381 (22%)
60-64	328 (22%)	377 (22%)
≥65	374 (25%)	439 (26%)
NASH CRN stage (%)		
F3	660 (44%)	740 (43.5%)
F4	853 (56%)	963 (56.5%)
Missing	2 (0.1%)	N/A
Median ELF score (IQR)	10.31 (9.69, 11.02)	10.26 (9.64, 10.96)
Median FIB-4 score (IQR)	2.16 (1.44, 3.24)	2.13 (1.44, 3.08)
Median VCTE in kPa (IQR)	16.8 (11.6, 25.2)	16.6 (11.5, 24.0)

ELF, enhanced liver fibrosis; FIB-4, fibrosis-4; LRE, liver-related event; NASH, non-alcoholic steatohepatitis; VCTE, vibration-controlled transient elastography.

### Prognostic alignment

In this dataset, the probability of LREs increased non-linearly with baseline ELF, remaining low across much of the lower range before rising sharply at ELF values greater than 11.30, indicating a pronounced inflection at the upper end of the distribution (Fig. 1A). In contrast, baseline VCTE showed a more gradual increase in event probability across its range, without a clear inflection point (Fig. 1B). Given the non-linear relationship of ELF and VCTE with LRE risk, prognostic alignment was performed by identifying VCTE thresholds associated with event risks comparable to predefined ELF cut-offs.

**Fig. 1:**
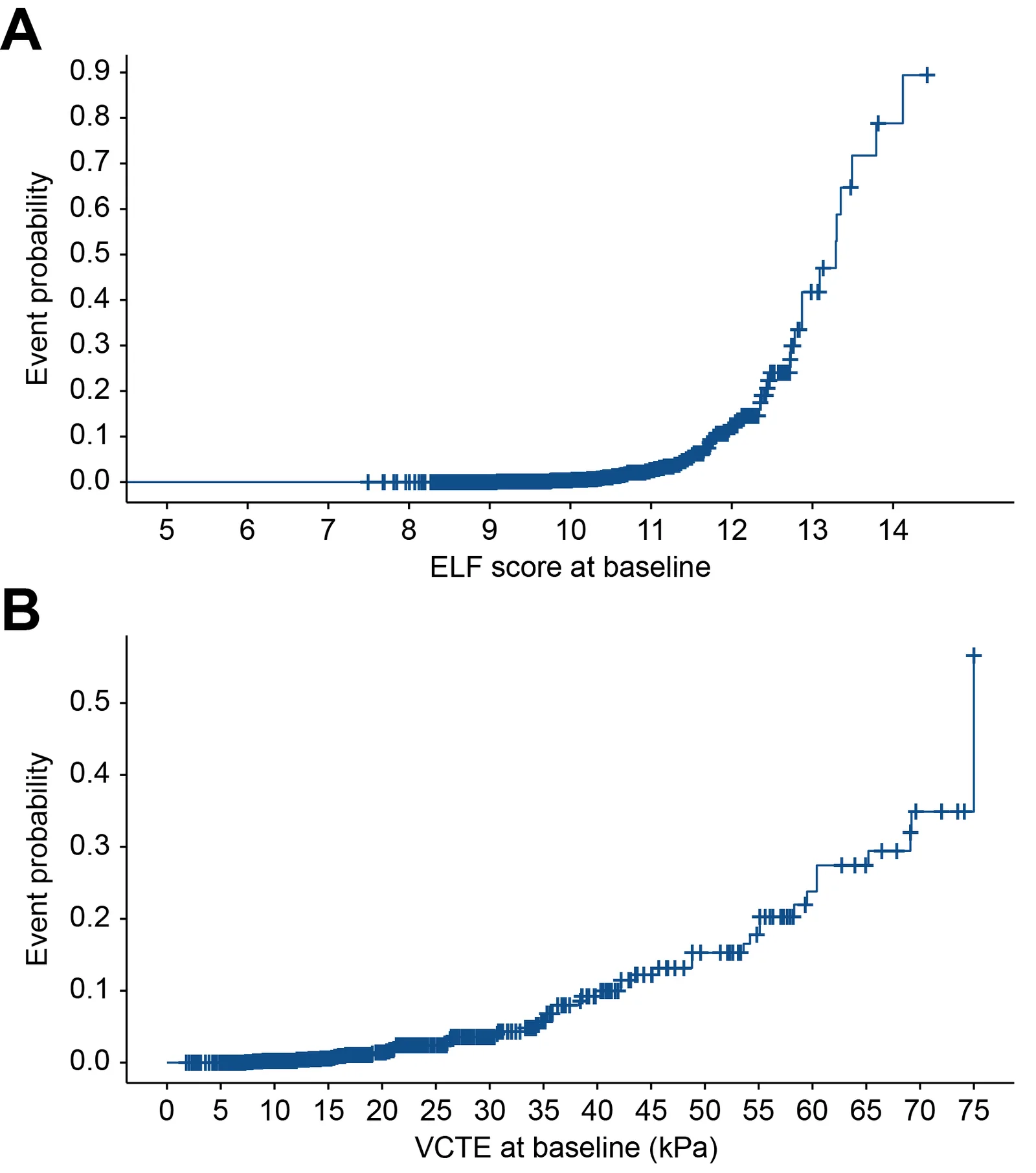
Cumulative probability of liver-related clinical outcomes according to baseline NIT value, estimated using the Kaplan-Meier method. (A) ELF, (B) VCTE (kPa). ELF, enhanced liver fibrosis; NITs, non-invasive tests; VCTE, vibration-controlled transient elastography.

**Fig. 2:**
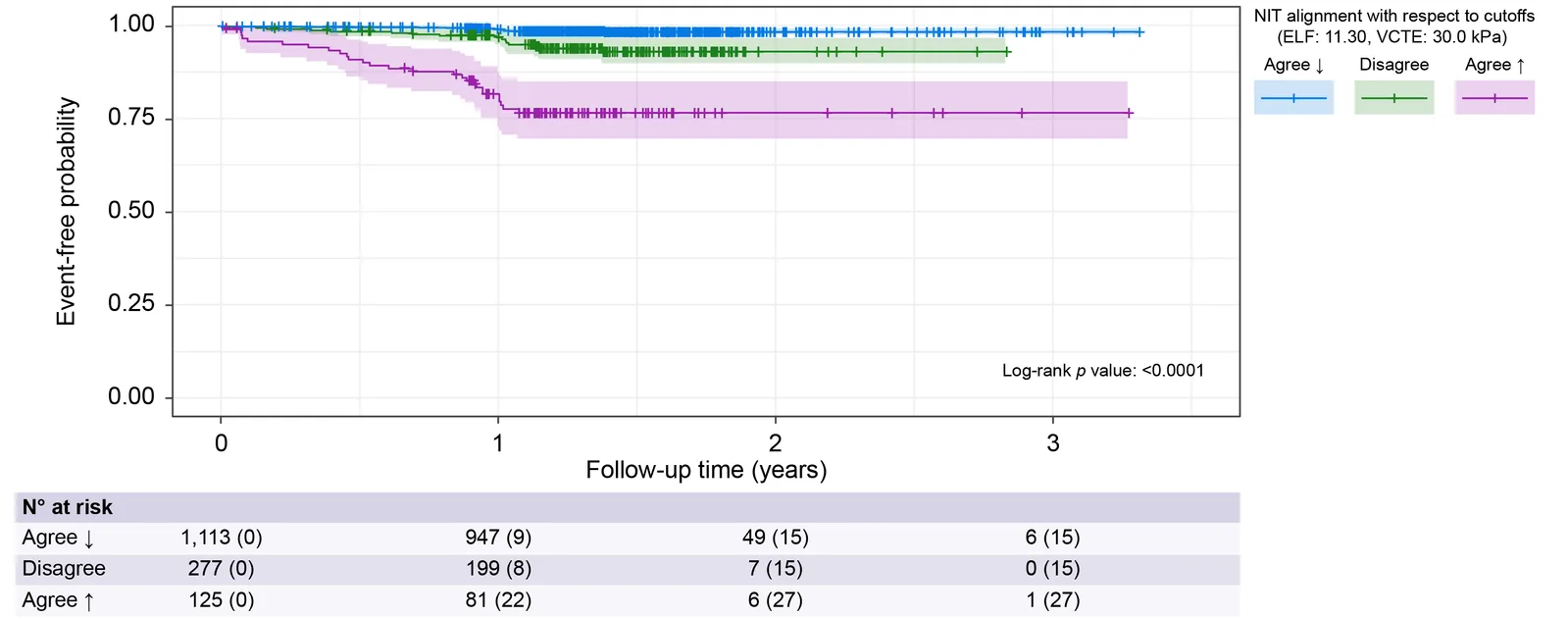
Kaplan-Meier curves for event-free probability of LREs Risk stratification based on NIT alignment with respect to cut-offs (ELF:11.30 and VCTE:30.0 kPa). “Agree ↓” (Blue): ELF <11.30 and VCTE <30.0 kPa; “Disagree” (Green): ELF ≥11.30 and VCTE <30.0 kPa OR ELF <11.30 and VCTE ≥30.0 kPa; “Agree ↑” (Purple): ELF ≥11.30 and VCTE ≥30.0 kPa. ELF, enhanced liver fibrosis; LREs, liver-related events; NITs, non-invasive tests; VCTE, vibration-controlled transient elastography.

Patients with baseline ELF scores greater than or equal to 7.70, 8.30, 9.00, 9.80, 10.50, 11.30, 12.00, and 12.80 had LRE risks of 3.8%, 3.8%, 4.1%, 4.9%, 7.2%, 12.8%, 21.3%, and 47.4%, respectively. VCTE cut-offs with event risks comparable to those for the predetermined ELF thresholds were 2.5, 5.0, 9.6, 12.4, 18.6, 29.2, 51.8, and 75.0 kPa, respectively. Although baseline ELF ≥12.80 was associated with a 47.4% risk of LREs, the highest point estimate for risk obtainable by VCTE (at the upper analytical range of 75 kPa) was 33.3% (Table 2). Inverse mapping to identify ELF cut-offs corresponding to established VCTE thresholds yielded similar findings (Table S4), and similar trends were observed when aligning ELF and FIB-4 cut-offs (Table S5). Additionally, the LRE risk associated with NASH CRN stage F4 in this population was 5.9% (95% CI 4.4–7.7%), with ELF and VCTE cut-offs of ≥10.14 and ≥15.5 kPa, respectively, corresponding to an equivalent risk. Conversely, the LRE risk associated with F3 fibrosis in this population was 1.1% (95% CI 0.4–2.2%), corresponding to ELF and VCTE cut-offs of <10.39 and <14.0 kPa, respectively (Table S6). Similar findings were observed when analyses were restricted to events occurring within 18 months of baseline assessment (Table S7).

**Table 2:** Corresponding VCTE thresholds to established ELF cut-offs with respect to risk of LREs.

ELF		VCTE				
Established ELF cut-off	LRE risk (95% CI)	Empirically derived cut-off (kPa)	Cut-off range (kPa)^a^	LRE risk (95% CI)	Nearest VCTE cut-off in clinical use (kPa)	LRE risk (95% CI)
≥7.70	3.8% (2.9%–4.9%)	≥2.5	1.8 to 6.4	3.8% (2.9%–4.9%)	≥5.0	3.8% (2.9%–4.9%)
≥8.30	3.8% (2.9%–4.9%)	≥5.0	1.8 to 7.4	3.8% (2.9%–4.9%)		
≥9.00	4.1% (3.1%–5.3%)	≥9.6	8.6 to 10.0	4.1% (3.1%–5.3%)	≥10.0^c^	4.2% (3.1%–5.4%)
≥9.80	4.9% (3.7%–6.4%)	≥12.4	12.0 to 12.5	4.9% (3.7%–6.4%)	≥12.0	4.8% (3.6%–6.2%)
≥10.50	7.2% (5.3%–9.4%)	≥18.6	18.4 to 19.6	7.2% (5.3%–9.5%)	≥20.0	7.3% (5.4%–9.7%)
≥11.30	12.8% (9.1%–17.3%)	≥29.2	28.9 to 29.3	12.8% (9.1%–17.5%)	≥30.0^d^	13.4% (9.5%–18.3%)
≥12.00	21.3% (13.5%–30.9%)	≥51.8	51.5 to 54.8	21.3% (13.5%–30.9%)	N/A	N/A
≥12.80	47.4% (24.4%–71.1%)	≥75.0^a^	Not calculable	33.3% (11.8%–61.6%)^b^	N/A	N/A

ELF, enhanced liver fibrosis; LRE, liver-related event; VCTE, vibration-controlled transient elastography.

a Range of VCTE thresholds associated with an observed LRE risk within ±0.1 percentage points of the corresponding ELF-associated risk.

b A VCTE threshold matching the LRE risk of ELF ≥12.80 was not identified. 75 kPa reflects the upper limit of the analytical range for LSM by VCTE.

c The LRE risk at VCTE ≥8.0 kPa is 4.0% (3.0%, 5.2%) and may be considered an alternate aligned cut-off for ELF ≥9.00.

d The LRE risk at VCTE ≥25.0 kPa is 9.9% (7.1%, 13.4%).

Many of the derived cut-offs were close to pre-established VCTE cut-offs of 5.0, 10.0, 12.0, 20.0, and 30.0 kPa. The risk of LREs at each of these pre-established cut-offs was not significantly different from the risk for the experimentally derived thresholds (Table 2). Similar trends between ELF and VCTE at the various cut-offs were observed using 12-month event risk to account for differences in follow-up time between patients (Table S8). Given that the same ELF and VCTE cut-offs are commonly used to assess liver fibrosis stage, diagnostic performance at these aligned thresholds was also evaluated against histology. At aligned cut-offs, ELF and VCTE demonstrated broadly comparable sensitivity and specificity with respect to histologic fibrosis stage, consistent with the intended use of these thresholds (Table S16–S20; Fig. S6).

### NIT combinations

Having established ELF and VCTE clinical cut-offs aligned to predict LREs, we next evaluated the utility of combining these tests at their aligned thresholds to enhance near-term risk stratification (Table 3). For the ELF 9.80/VCTE 12.0 kPa combination, event risk remained low until both NITs were above threshold. For the ELF 10.50/VCTE 20.0 kPa and ELF 11.30/VCTE 30.0 kPa combinations, event risk was lowest when both NITs were below threshold (negative concordance), increased with discordance, and was highest when both tests were concordant above threshold (positive concordance). For example, within the ELF 11.30/VCTE 30.0 kPa pairing, event risk rose stepwise from 1.3% (95% CI 0.8%–2.2%) with negative concordance, to 5.4% (95% CI 3.1%–8.8%) with discordance, and to 21.6% (95% CI 14.7%–29.8%) with positive concordance. This stepwise increase in LRE risk across ELF and VCTE strata is illustrated in two-dimensional risk heatmaps (Fig. S2), which highlight the concentration of highest risk in patients with concordantly elevated ELF and VCTE values.

**Table 3:** Risk of liver-related clinical outcomes stratified by ELF and VCTE combinations.

Interpretation	ELF and VCTE relative to respective cut-offs	Total patients	Patients with events	LRE risk (95% CI)^a^	Likelihood ratio^b^ (95% CI)	LRE incidence per 1,000 PY (95% CI)	Cox proportional hazard ratio (95% CI) [*p* value]
Agree below (↓)	ELF <9.80; VCTE <12.0 kPa	206	2	1.0% (0.1%–3.5%)	0.25 (0.06–0.98)	6.9 (0.0–17.3)	1.00 (Reference)
Disagree	ELF <9.80; VCTE ≥12.0 kPa	233	2	0.9% (0.1%–3.1%)	0.22 (0.06–0.87)	6.2 (0.0–15.6)	—
	ELF ≥9.80; VCTE <12.0 kPa	205	2	1.0% (0.1%–3.5%)	0.25 (0.06–0.98)	6.9 (0.0–17.3)	—
	Total	438	4	1.0% (0.1%–3.5%)	0.24 (0.09–0.61)	6.6 (1.6–13.2)	0.99 (0.18–5.42) [*p =* 0.9924]
Agree above (↑)	ELF ≥9.80; VCTE ≥12.0 kPa	871	51	5.9% (4.4%–7.6%)	1.59 (1.44–1.76)	45.7 (34.0–58.2)	6.59 (1.60–27.07) [*p =* 0.0089]

Agree below (↓)	ELF <10.50; VCTE <20.0 kPa	640	4	0.6% (0.2%–1.6%)	0.16 (0.06–0.41)	4.5 (1.1–8.9)	1.00 (Reference)
Disagree	ELF <10.50; VCTE ≥20.0 kPa	221	6	2.7% (1.0%–5.8%)	0.71 (0.33–1.54)	20.3 (6.8–37.2)	—
	ELF ≥10.50; VCTE <20.0 kPa	275	9	3.3% (1.5%–6.1%)	0.87 (0.47–1.59)	25.3 (11.2–42.2)	—
	Total	496	15	3.0% (1.7%–4.9%)	0.80 (0.51–1.24)	23.0 (12.3–35.3)	5.14 (1.71–15.48) [*p =* 0.0036]
Agree above (↑)	ELF ≥10.50; VCTE ≥20.0 kPa	379	38	10.0% (7.2%–13.5%)	2.85 (2.32–3.50)	82.0 (56.1–110.1)	18.12 (6.46–50.78) [*p <*0.0001]

Agree below (↓)	ELF <11.30; VCTE <30.0 kPa	1,113	15	1.3% (0.8%–2.2%)	0.35 (0.23–0.54)	9.9 (5.3–15.2)	1.00 (Reference)
Disagree	ELF <11.30; VCTE ≥30.0 kPa	128	7	5.5% (2.2%–10.9%)	1.48 (0.72–3.02)	41.3 (11.8–76.7)	—
	ELF ≥11.30; VCTE <30.0 kPa	149	8	5.4% (2.3%–10.3%)	1.45 (0.75–2.81)	42.8 (16.1–74.9)	—
	Total	277	15	5.4% (3.1%–8.8%)	1.46 (0.94–2.29)	42.1 (22.5–64.6)	4.31 (2.11–8.81) [*p <*0.0001]
Agree above (↑)	ELF ≥11.30; VCTE ≥30.0 kPa	125	27	21.6% (14.7%–29.8%)	7.05 (5.05–9.84)	188.2 (118.5–264.9)	19.23 (10.22–36.18) [*p <*0.0001]

ELF, enhanced liver fibrosis; LRE, liver-related event; PY, patient-years; VCTE, vibration-controlled transient elastography.

*p* values for the respective ELF/VCTE cut-off pairings were: 9.80/12.0 kPa (*p =* 1.000), 10.50/20.0 kPa (*p =* 0.7966), and 11.30/30.0 kPa (*p =* 1.000).

a Fisher’s exact test was used to compare LRE rates between discordant groups (VCTE < cut-off & ELF ≥ cut-off *vs*. ELF < cut-off & VCTE ≥ cut-off).

b LR = proportion of events in stratum / proportion of non-events in stratum.

Across these cut-off pairings, event risk in the concordantly positive strata approximately doubled with increasing thresholds, from 5.9% (95% CI 4.4%–7.6%) for ELF 9.80/VCTE 12.0 kPa, to 10.0% (95% CI 7.2%–13.5%) for ELF 10.50/VCTE 20.0 kPa, and 21.6% (95% CI 14.7%–29.8%) for ELF 11.30/VCTE 30.0 kPa. Event risk did not differ significantly by Fisher’s exact test between discordant strata, regardless of which test exceeded threshold. The ELF 9.80/VCTE 12.0 kPa pairing showed event risk comparable to NASH CRN F4 staging (5.9%), whereas higher threshold pairings were associated with markedly higher risk (Table S6).

To further characterize the discordant strata, baseline laboratory characteristics were compared across concordance groups (Table S8). Although patients with discordant results demonstrated similar LRE risks regardless of whether ELF or VCTE exceeded the threshold, biochemical profiles were not identical. Patients with ELF ≥11.30 and VCTE <30.0 kPa had higher AST, ALT, and ALP concentrations than patients with ELF <11.30 and VCTE ≥30.0 kPa, whereas platelet counts, GGT, and bilirubin levels were comparable between discordant groups. These findings suggest that ELF and VCTE may identify partially overlapping but distinct manifestations of advanced liver disease despite being associated with similar intermediate clinical risk.

Analyses using alternative, non-aligned cut-offs showed comparable discrimination, with modest variation in absolute risk. The positive concordant stratum for the ELF 11.30/VCTE 25.0 kPa pairing demonstrated slightly lower event risk (18.5%; 95% CI 12.7%–25.4%) than the corresponding aligned ELF 11.30/VCTE 30.0 kPa pairing (Tables S6 and S9). In addition, a three-NIT concordance approach incorporating ELF, VCTE, and FIB-4 yielded qualitatively similar risk stratification, with the highest LRE risk observed in the positive concordant stratum (26.2%; 95% CI 17.2%–36.9%) (Table S10).

As the ELF 11.30/VCTE 30.0 kPa pairing yielded the highest LRE risk across all two-NIT analyses evaluated, it was selected for further evaluation. Kaplan-Meier analysis showed clear and graded separation in event-free probability across concordant and discordant strata within the ELF 11.30/VCTE 30.0 kPa group (Fig. 2). By comparison, stratification by NASH CRN stage yielded minimal separation between F3 and F4 (Fig. S3), while the three-NIT concordance model showed comparable separation to the ELF/VCTE combination (Fig. S4).

To evaluate whether this framework also reflects underlying histological disease activity, we assessed its association with fibrosis progression and regression using thresholds relevant to fibrosis staging. Using lower aligned cut-offs commonly used for identification of advanced fibrosis (ELF 9.80 and VCTE 12.0 kPa),^3^^,^^9^ we next evaluated whether agreement-based combinations of ELF and VCTE stratified the likelihood of short-term histological progression and regression (Table 4). Among patients with baseline F3 fibrosis, the probability of progression to F4 increased across concordance strata, rising from 2.2% (95% CI 0.6%–5.7%) in the negative agreement group to 25.4% (95% CI 20.3%–31.0%) in the positive agreement group, with intermediate risk observed in discordant strata (14.4%; 95% CI 10.6%–18.9%). Conversely, among patients with baseline F4 fibrosis, the likelihood of regression to <F4 was highest in the negative agreement group (56.9%; 95% CI 43.2%–69.8%) and lowest in the positive agreement group (12.2%; 95% CI 9.9%–14.9%), with discordant strata demonstrating intermediate probabilities (27.3%; 95% CI 21.6%–33.6%). Similar to the analyses of liver-related clinical outcomes, rates of histological change did not differ significantly between discordant strata, regardless of whether ELF or VCTE exceeded the threshold. These findings demonstrate a consistent, graded relationship between ELF/VCTE concordance and histological disease dynamics, whereby concordantly elevated values identify patients at higher risk of progression and lower likelihood of regression, while concordantly low values indicate a more favorable histological trajectory. We next evaluated whether this agreement-based risk stratification translated into improved model discrimination compared with guideline-based single-test approaches.

**Table 4:** Likelihood of histological progression and regression at 1 year stratified by ELF and VCTE combinations.

Interpretation	ELF and VCTE relative to respective cut-offs	Total patients	Patients with events	Event rate (95% CI)^a^	Likelihood ratio^b^ (95% CI)
**Histological progression from F3 to F4**^c^					
Agree below (↓)	ELF <9.80; VCTE <12.0 kPa	178	4	2.2% (0.6%–5.7%)	0.13 (0.05–0.33)
Disagree	ELF <9.80; VCTE ≥12.0 kPa	145	23	15.9% (10.3%–22.8%)	1.03 (0.69–1.53)
	ELF ≥9.80; VCTE <12.0 kPa	146	19	13.0% (8.0%–19.6%)	0.82 (0.53–1.27)
	Total	291	42	14.4% (10.6%–19.0%)	0.92 (0.71–1.19)
Agree above (↑)	ELF ≥9.80; VCTE ≥12.0 kPa	271	69	25.5% (20.4%–31.1%)	1.86 (1.54–2.25)
Overall	—	740	115	15.5% (13.0%–18.4%)	—

**Histological regression from F4 to <F4**^d^					
Agree below (↓)	ELF <9.80; VCTE <12.0 kPa	58	33	56.9% (43.2%–69.8%)	5.82 (3.55–9.54)
Disagree	ELF <9.80; VCTE ≥12.0 kPa	139	37	26.6% (19.5 %–34.8%)	1.60 (1.14–2.25)
	ELF ≥9.80; VCTE <12.0 kPa	88	25	28.4% (19.3%–39.0%)	1.75 (1.13–2.70)
	Total	227	62	27.3% (21.6%–33.6%)	1.66 (1.30–2.11)
Agree above (↑)	ELF ≥9.80; VCTE ≥12.0 kPa	678	83	12.2% (9.9%–14.9%)	0.62 (0.52–0.72)
Overall	—	963	178	18.5% (16.1%–21.1%)	—

*P* values for the progression and regression analyses were *p* = 0.5093 and *p* = 0.7625, respectively.

a Fisher’s exact test was used to compare rates of histological change between discordant groups (VCTE <12.0 kPa & ELF ≥9.80 *vs*. ELF <9.80 & VCTE ≥12.0 kPa).

b LR = proportion of events in stratum / proportion of non-events in stratum.

c Baseline: all patients staged as NASH CRN F3. Events: histological staging of F4 at Week 48.

d Baseline: all patients staged as NASH CRN F4. Events: histological staging of F0 to F3 at Week 48/52.

### Model comparisons

In comparisons of LRE prognostication models, agreement-based combinations of ELF and VCTE demonstrated greater discrimination for LREs than either test alone (Table S11). While ELF alone demonstrated better discrimination than VCTE alone (C-index 0.758 *vs*. 0.708), the combination of ELF 11.30 and VCTE 30.0 kPa demonstrated the highest discrimination (C-index = 0.786) and best model fit (lowest AIC/BIC). Overall, these findings suggest that the combination of ELF and VCTE provides incremental value primarily at higher, concordant risk thresholds, where agreement between markers identifies a subgroup with more distinct risk stratification.

ROC analyses showed a similar pattern, with improved discrimination for the ELF/VCTE combination compared with models using either test alone at typical thresholds. In logistic regression-based step-function ROC analyses (Fig. 3A), the ELF/VCTE combination achieved a higher AUROC (0.779; 95% CI 0.714, 0.844) than VCTE alone (0.705; 95% CI 0.651, 0.760; DeLong *p =* 0.0129), while differences between the ELF/VCTE combination and ELF alone (AUROC 0.753; 95% CI 0.692, 0.815) were not statistically significant (*p =* 0.2632). Similar patterns were observed in Cox regression-based ROC analyses at 12 months (Fig. 3B), with the ELF/VCTE combination demonstrating the highest discrimination (AUROC 0.801; 95% CI 0.711, 0.848), compared with ELF (0.790; 95% CI 0.696, 0.825) and VCTE (0.694; 95% CI 0.650, 0.769).

**Fig. 3:**
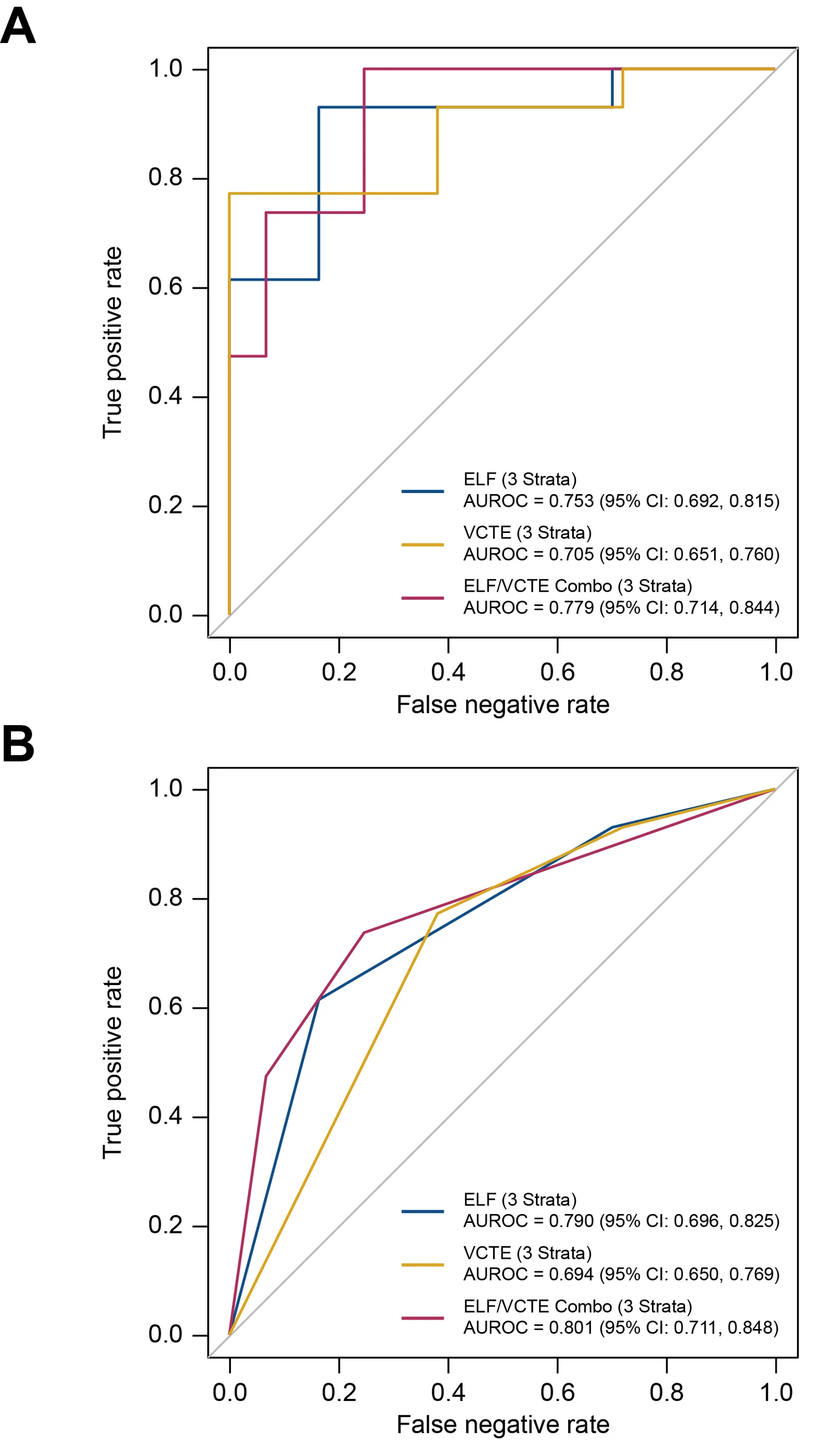
Receiver-operating characteristic curves by measurement of baseline ELF (stratified by cut-offs at 9.80 and 11.30), VCTE (stratified by cut-offs at 12.0 and 20.0 kPa), or ELF and VCTE combination (agreement-based stratification using 11.30 and 30.0 kPa cut-offs). (A) Logistic regression-based step-function ROC curves. *p* values of DeLong’s test: ELF *vs*. VCTE (*p =* 0.1448), ELF/VCTE Combo *vs*. ELF (*p =* 0.2632), ELF/VCTE Combo *vs*. VCTE (*p =* 0.0129). (B) Cox regression-based ROC curves at 12 months. AUROC, area under the ROC curve; ELF, enhanced liver fibrosis; ROC, receiver-operating characteristic; VCTE, vibration-controlled transient elastography.

Time-dependent ROC analyses further supported the discriminative utility of the ELF/VCTE combination over follow-up (Fig. S5; Table S12). Across evaluated time points from 3 to 12 months, AUC(t) values for the ELF/VCTE combination were significantly higher than those for VCTE alone (all *p <*0.01). Although AUC(t) values for the ELF/VCTE combination remained numerically higher than those for VCTE through later follow-up, these differences were not statistically significant beyond 1 year. Relative to ELF alone, AUC(t) values for the ELF/VCTE combination were comparable.

Collectively, these findings indicate that agreement-based combinations of ELF and VCTE maintain or improve discrimination for LREs relative to either test alone, particularly compared with VCTE, and complement the risk stratification patterns observed in concordance and time-to-event analyses.

## Discussion

Our study generated several insights with potential implications for clinical practice. Current clinical guidelines recommend ELF and VCTE thresholds that are often interpreted as broadly equivalent across tests. However, these thresholds are based on independent studies, were typically validated against histological fibrosis stage and have not been explicitly calibrated to yield equivalent risk of LREs across tests. For example, commonly cited guidelines associate ELF 7.70 with VCTE 8.0 kPa, ELF 9.80 with VCTE 12.0 kPa, and ELF 11.30 with VCTE 20.0 kPa.^3^^,^^9^^,^^37^^,^^38^ While our analyses confirm the pairing of ELF 9.80/VCTE 12.0 kPa, they suggest that the lower and upper thresholds may be more appropriately aligned as ELF 9.00/VCTE 8.0 kPa and ELF 11.30/VCTE 30.0 kPa, respectively. These findings indicate that risk-aligned threshold pairings may enable more consistent prognostic assessment in patients with MASH.

While ELF ≥11.30 and VCTE ≥30.0 kPa were each individually associated with an event risk of approximately 13%, concordance at these thresholds was associated with a substantially higher risk of 21.6%. In contrast, when the tests were discordant, with one above and one below threshold, the event risk was lower, at 5.4%. This pattern suggests a synergistic rather than additive effect, likely due to ELF and VCTE capturing distinct but converging biological processes of matrix turnover and architectural distortion, respectively.^39^ Discordance of ELF and VCTE may indicate an intermediate phase of fibroinflammatory injury with elevated near-term risk and likely reflects a clinically meaningful transitional state. Rather than defaulting to the “more severe” test or dismissing the discrepancy, clinicians could consider discordance itself as a marker of elevated risk that warrants closer monitoring or further evaluation. Although patients in this putative transitional phase may be ideal candidates for treatment, recent recommendations exclude patients with either ELF ≥11.30 or VCTE ≥20.0 kPa from treatments indicated for patients with F2/F3 fibrosis.^40^^,^^41^ Analyses from ongoing phase III studies evaluating clinical and liver-related outcomes will likely be needed to determine whether patients with discordant ELF and VCTE measurements respond to MASH therapeutics.

The biochemical analysis of discordant ELF and VCTE results provides additional insight into the potential biological basis of risk stratification. Although patients with ELF <11.30/VCTE ≥30.0 kPa and ELF ≥11.30/VCTE <30.0 kPa had nearly identical LRE risks, their biochemical profiles were not identical. Patients with ELF ≥11.30/VCTE <30.0 kPa had significantly higher AST and ALT concentrations, and modestly higher ALP concentrations, than patients with ELF <11.30/VCTE ≥30.0 kPa, whereas platelet count, GGT, and bilirubin levels were comparable between discordant groups. These findings suggest that patients identified as high-risk by ELF alone may exhibit greater evidence of ongoing hepatocellular injury despite having LSM below the highest-risk threshold. Conversely, patients identified as high-risk by VCTE alone may have comparable clinical risk despite less pronounced biochemical evidence of liver injury. Together, these observations support the concept that ELF and VCTE capture partially overlapping but distinct biological dimensions of advanced liver disease and provide complementary prognostic information when interpreted together.

A similar pattern was observed when the aligned cut-off framework was evaluated against histological progression and regression using lower aligned thresholds of ELF 9.80 and VCTE 12.0 kPa. Among patients with baseline F3 fibrosis, concordantly elevated ELF and VCTE values were associated with the highest likelihood of progression to F4, whereas concordantly low results identified patients with the lowest likelihood of progression. Conversely, among patients with baseline F4 fibrosis, concordantly low ELF and VCTE values were associated with the greatest likelihood of regression to <F4, while concordantly elevated values identified patients least likely to regress. Notably, rates of histological change were similar between discordant strata, regardless of whether ELF or VCTE exceeded the threshold. The consistency of concordance patterns across both histological and clinical endpoints suggests that the relationship between ELF/VCTE agreement and risk reflects a broader biological continuum of disease severity and progression rather than an association specific to a single outcome measure. Together, these findings suggest that agreement between ELF and VCTE reflects underlying disease biology, and that discordance may represent an intermediate disease state characterized by neither the low-risk profile associated with concordantly negative results nor the high-risk profile associated with concordantly positive results.

Although this dataset included a high proportion of patients with F4 fibrosis (56%), the findings from this study have important implications for the design of non-cirrhotic MASH clinical trials. Natural history studies suggest that the expected rate of decompensation in patients with F2 or F3 fibrosis is approximately 2% over 4 years.^26^ Given that current MASH therapeutic trials typically span 4-5 years, most primary endpoints are likely to reflect progression to cirrhosis.[42], [43], [44] As a result, despite efforts to replace histological endpoints with non-invasive alternatives for accelerated approval, traditional approval will likely continue to rely on histologically confirmed progression unless a validated surrogate endpoint is qualified.^45^

The US FDA has indicated that specific NITs, or combinations thereof, may qualify as surrogate endpoints if highly predictive of clinical outcomes.^45^ Our study provides evidence that ELF ≥11.30 and VCTE ≥30.0 kPa are individually associated with more than double the risk of LREs compared with histologically staged F4, and that concordance at these thresholds is associated with nearly threefold higher risk. This suggests that the combination of ELF and VCTE at these thresholds represents a potential candidate for a validated surrogate endpoint, while also reducing potential analytical noise associated with false results from a single test. Notably, even discordant results at these thresholds confer a risk comparable to histological F4, offering additional reassurance that this combination identifies a clinically meaningful high-risk population. Further studies are warranted to determine whether this combination can support biomarker qualification as a validated surrogate endpoint.

In this study, we evaluated a VCTE threshold of 30.0 kPa, extending beyond the highest guideline-recommended cut-off of 25.0 kPa proposed in the Baveno VII consensus to rule-in clinically significant portal hypertension. While a VCTE threshold of 25.0 kPa was associated with slightly lower LRE risk than a threshold of 30.0 kPa when used alone or in combination with ELF, these differences were not statistically significant. Importantly, when paired with an ELF threshold of 11.30, both VCTE cut-offs identified patients at similarly elevated risk, suggesting that 25.0 kPa represents an acceptable threshold for clinical application within combined ELF/VCTE risk stratification frameworks. However, the use of 30.0 kPa provided greater separation of prognostic risk strata, supporting its use when the objective is greater discrimination of advanced risk rather than alignment with clinically significant portal hypertension rule-in criteria alone.

There are some limitations to this study. The primary analyses in this study were limited to ELF and VCTE, which were selected based on data availability and their prominent inclusion in current clinical practice guidance.^3^^,^^9^ Although a growing number of newer non-invasive markers and composite scores (*e.g*. FAST, MAST, Agile scores, NIS4/NIS2+) have been developed,[46], [47], [48], [49] these were not available for inclusion in the present dataset. Future studies incorporating both established and emerging NITs may further refine risk-aligned threshold combination strategies.

An additional limitation relates to the pooling of data from five clinical trials, including participants from both placebo and treatment arms. While this approach increased statistical power, it also introduced heterogeneity in the study design. Although no significant differences in LRE risk were observed across ELF strata between treatment and placebo arms and in other sensitivity analyses (Tables S13–S15), the potential influence of investigational therapies on disease course cannot be entirely excluded. Additionally, all biopsies were read by a single pathologist, which reduced inter-reader variability, but may have introduced interpretation bias.

It should also be recognized that the risk-aligned ELF and VCTE thresholds were derived empirically from observed event rates within this pooled clinical trial dataset. Although threshold-tolerance analyses suggested that the principal clinically relevant alignments were relatively stable across small differences in observed LRE risk, these analyses do not account for all sources of variability, including biological variability, analytical variability, measurement variability, or VCTE reliability characteristics. In particular, interquartile range-to-median ratio (IQR/M) data were not available for VCTE measurements. Consequently, the potential impact of LSM reliability on risk stratification and threshold alignment could not be assessed.

In this study, participants were enrolled through clinical trial eligibility criteria and therefore represent a selected population characterized by older age, advanced liver disease, and a high prevalence of advanced fibrosis. As a result, the observed risk relationships and threshold alignments may not be directly applicable to younger patients, individuals with less advanced fibrosis, or routine clinical practice populations. The absence of an external validation cohort represents a limitation of the present study. Therefore, although the proposed risk-aligned thresholds were supported by internal consistency across analyses of LREs and histological change, they were derived from a selected advanced-fibrosis clinical trial population with a limited number of clinical events. Independent validation in external real-world and clinical trial cohorts will be necessary to establish their reproducibility, generalizability, and clinical utility.

Despite these limitations, this study demonstrates that ELF and VCTE cut-offs can be aligned to yield broadly comparable prognostic performance. Accordingly, modest refinement of guideline-recommended thresholds may be informative when these methods are interpreted in relation to one another for LRE risk staging, rather than as strictly diagnostic surrogates. In addition, our findings show that combining ELF and VCTE can improve prognostication of liver-related clinical outcomes, particularly in higher risk strata. Collectively, these results may support more consistent risk assessment in specialist settings and improve patient enrichment strategies in clinical trials of therapeutics for MASH.

## Abbreviations

AUROC, area under the ROC curve; ELF, enhanced liver fibrosis; LRE, liver-related event; LSM, liver stiffness measurement; MASH, metabolic dysfunction-associated steatohepatitis; MASLD, metabolic dysfunction-associated steatotic liver disease; NAFLD, nonalcoholic fatty liver disease; NASH CRN, Nonalcoholic Steatohepatitis Clinical Research Network; NITs, non-invasive tests; ROC, receiver-operating characteristic; VCTE, vibration-controlled transient elastography.

## Authors’ contributions

MG: study concept and design, interpretation of data, and drafting of manuscript; ANB: study concept, review and editing of manuscript; DBC: interpretation of data, review and editing of manuscript; RF, ED and CB: statistical analysis, interpretation of data, review and editing of manuscript; AJS, JJF and KP: study supervision, data interpretation, critical revision of the manuscript.

## Data availability

The data underlying this study were obtained under data sharing agreements and cannot be made publicly available by the authors. Access to the original datasets is subject to the approval and data-sharing policies of the respective data owners.

## Financial support

This study was supported by 10.13039/501100004830Siemens Healthcare Diagnostics Inc. (USA) and by the 10.13039/501100004191Novo DevSTAR programme (Denmark).

## Conflicts of interest

MG, DBC, RF, ED and RB are employees of Siemens Healthineers. ANB was an employee and is a stockholder of Gilead Sciences; KP is consultant for Novo, Merck, and Resalis, and has received grants from Novo. AJS has stock options in Durect, Inversago, Tiziana, Rivus, Exhalenz, Genfit. He has served as a consultant to Intercept, Gilead, Takeda, Meck, Eli Lilly, Novo, Astra Zeneca, Boehringer Ingelheim, Alnylam, Regeneron, Histoindex, Path AI, Pfizer, 89Bio, Altimmune, Northsea, Akero, Madrigal, Salix, Myovant, Poxel, Surrozen, Hanmi, Aligos, Promed, Zydus. His institution has received grants from Intercept, Novo, Eli Lilly, Boehringer Ingelheim, Echosens, Hanmi, Madrigal, Gilead, Salix, Meck, Takeda. He receives royalties from Elsevier and Wolter Kluwers. KP has served as a consultant to Gilead Sciences, Galectin Therapeutics, Novo, Resalis, Inipharm, and Merck. He has received grants from Novo, Madrigal, Gilead Sciences, Merck, GlaxoSmithKline, and 89bio.

Please refer to the accompanying ICMJE disclosure forms for further details.
