# Supplementary material for: Aligned ELF and VCTE cut-offs facilitate risk staging in patients with metabolic dysfunction-associated steatohepatitis
Source: JHEP Rep. 2026 Sep 18;8(12):102041. doi: 10.1016/j.jhepr.2026.102041 (PMC13770651; doi:10.1016/j.jhepr.2026.102041)
Supplement: Supplementary file 1 [file mmc1.pdf]

Aligned ELF and VCTE cutoffs facilitate risk staging in patients with metabolic dysfunction-associated steatohepatitis

Matthew F.W. Gee, Andrew N. Billin, Donald B. Chalfin, Rachel Fonstad, Edward B. De Vol, Jordan J. Feld, Keyur Patel, Arun J. Sanyal

Table of Contents

1. Study Population and Baseline Characteristics 2
2. Clinical Outcomes 5
3. Prognostic Alignment 6
 3.1 Methodology to Derive Equivalent Thresholds with Respect to LRE Risk 6
4. NIT Combinations....................12
5. Model Comparisons 19
6. Sensitivity Analyses....................23
7. Diagnostic Analyses 25
8. TRIPOD Statement Checklist 32
9. References....................36

1. Study Population and Baseline Characteristics

Fig. S1. Exclusions from liver-related event prognostic analyses

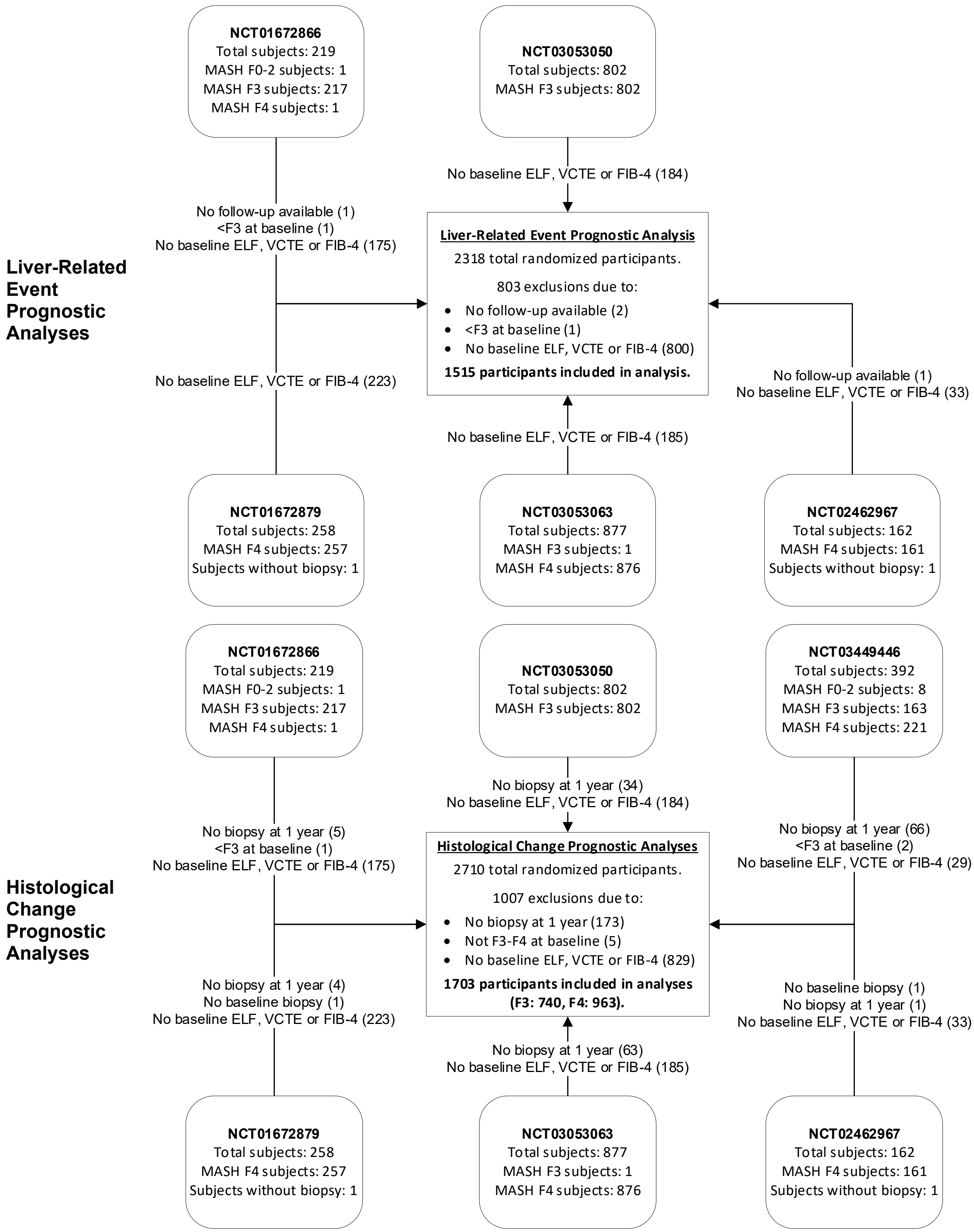

Table S1. Description of clinical trial datasets used for analyses

**Study Identifiers**	NCT01672866<br>GS-US-321-0105	NCT03053050<br>STELLAR-3	NCT01672879<br>GS-US-321-0106	NCT03053063<br>STELLAR-4	NCT02462967<br>NASH-CX	NCT03449446<br>ATLAS[a]
**Patient Population**	Bridging fibrosis (F3) due to MASH	Bridging fibrosis (F3) due to MASH	Compensated cirrhosis (F4) due to MASH	Compensated cirrhosis (F4) due to MASH	Compensated cirrhosis (F4) due to MASH	Bridging fibrosis (F3) & compensated cirrhosis (F4) due to MASH
**Phase**	Phase 2b	Phase 3	Phase 2b	Phase 3	Phase 2b	Phase 2b
**Study Drug**	Simtuzumab (Sim)	Selonsertib (Sel)	Simtuzumab (Sim)	Selonsertib (Sel)	Belapectin (Bel)	Selonsertib (Sel)<br>Firsocostat (Fir)<br>Cilofexor (Cilo)
**Randomization**	1:1:1<br>Sim 75 mg<br>Sim 125 mg<br>Placebo	2:2:1<br>Sel 6 mg<br>Sel 18 mg<br>Placebo	1:1:1<br>Sim 200 mg<br>Sim 700 mg<br>Placebo	2:2:1<br>Sel 6 mg<br>Sel 18 mg<br>Placebo	1:1:1<br>Bel 2 mg/kg<br>Bel 8 mg/kg<br>Placebo	1:1:1:2:2:2:1<br>Sel 18 mg<br>Fir 20 mg<br>Cilo 30 mg<br>Fir/Sel<br>Cilo/Sel<br>Cilo/Fir<br>Placebo
**Total Subjects in Study**	219	802	258	877	162	392
**Study Dates**	2012-2016	2017-2019	2012-2017	2017-2019	2015-2017	2018-2019
**Study Setting / Locations**	Refer to clinicaltrials.gov for full details					
**Eligibility Criteria**	Refer to clinicaltrials.gov for full details					
**Demographics**	Described in Harrison et al. (2018)[3] and Sanyal et al. (2019)[4]	Described in Harrison et al. (2020)[5] and Younossi et al. (2021)[6]	Described in Harrison et al. (2018),[3] Sanyal et al. (2019)[4] and Sanyal et al. (2022)[7]	Described in Harrison et al. (2020),[5] Younossi et al. (2021)[6] and Sanyal et al. (2022)[7]	Described in Chalasani et al. (2020)[8] and Are et al. (2021)[9]	Described in Loomba et al. (2021)[10]

[a] This dataset was not used for the liver-related event prognostic analyses.

Table S2. Distributions of VCTE and FIB-4 measurements at baseline arranged by ELF strata

ELF Score	N	VCTE (kPa)		FIB-4	
		Mean (SD)	Median (IQR)	Mean (SD)	Median (IQR)
<9.00	127	14.3 (9.5)	12.0 (8.9, 15.3)	1.37 (0.97)	1.20 (0.88, 1.58)
≥9.00 to <9.80	312	14.6 (8.1)	12.4 (9.5, 17.3)	1.71 (1.14)	1.46 (1.08, 2.06)
≥9.80 to <10.50	422	18.4 (11.1)	15.8 (11.4, 21.8)	2.18 (1.05)	2.00 (1.43, 2.67)
≥10.50 to <11.30	380	22.1 (12.5)	19.2 (14.3, 26.3)	3.02 (1.69)	2.64 (1.89, 3.64)
≥11.30 to <12.00	180	29.4 (17.1)	26.3 (16.8, 35.5)	3.87 (1.73)	3.49 (2.58, 4.97)
≥12.00 to <12.80	75	35.1 (17.6)	31.6 (21.3, 47.2)	4.79 (2.40)	4.36 (3.14, 6.12)
≥7.70	1511	20.6 (13.7)	16.8 (11.6, 25.4)	2.60 (1.72)	2.17 (1.44, 3.24)
≥8.30	1494	20.7 (13.7)	16.8 (11.7, 25.4)	2.62 (1.72)	2.19 (1.45, 3.26)
≥9.00	1388	21.2 (13.8)	17.3 (11.9, 26.0)	2.71 (1.73)	2.29 (1.52, 3.34)
≥9.80	1076	23.1 (14.5)	19.3 (13.3, 27.0)	3.00 (1.76)	2.58 (1.79, 3.65)
≥10.50	654	26.2 (15.7)	21.6 (15.4, 32.0)	3.52 (1.92)	3.09 (2.21, 4.36)
≥11.30	274	31.8 (17.8)	27.0 (18.7, 39.7)	4.22 (2.00)	3.81 (2.78, 5.29)
≥12.00	94	36.4 (18.2)	32.6 (21.3, 48.6)	4.89 (2.29)	4.38 (3.30, 6.13)
≥12.80	19	41.1 (20.3)	38.5 (25.9, 51.2)	5.31 (1.82)	4.41 (3.93, 6.79)

2. Clinical Outcomes

Table S3. Liver-related clinical events observed during patient follow-up

**Description of First Recorded Liver-Related Event**	**Number of Subjects with Liver-Related Clinical Outcome as a First Recorded Event**[a]
Clinically apparent ascites	16
Hepatic encephalopathy	12
Portal hypertension-related gastrointestinal events	20
Progression from small to medium or large varices	8
Newly diagnosed varies in those without prior varices	5
Esophageal variceal bleeding	1
Upper gastrointestinal bleeding	5
Gastropathy hemorrhage	1
Qualification for liver transplant (MELD Score ≥15)	4
≥2 increase in CPT Score	1
Liver-related death	0
Liver transplantation	1
Other decompensation event (description not available)	3
**Total**	**57**

[a] Only the first recorded clinical outcome was used in the analysis. Subsequent events occurring for these individuals was not considered.

3. Prognostic Alignment

3.1 Methodology to Derive Equivalent Thresholds with Respect to LRE Risk

All calculations were performed using R version 4.4.2. The following presents the workflow to empirically derive VCTE thresholds aligned in terms of LRE risk to established ELF cutoffs. The same approach was also used to derive ELF cutoffs from established VCTE thresholds, and to derive FIB-4 cutoffs from established ELF thresholds.

<u>Step 1: Determine LRE Risk at Target ELF Cutoffs</u>

Pre-specified ELF cutoffs were selected based on usage in guidelines and prior studies as follows: 7.70, 8.30, 9.00, 9.80, 10.50, 11.30, 12.00, 12.80.

$$c_{ELF} = \{7.70, 8.30, 9.00, 9.80, 10.50, 11.30, 12.00, 12.80\}$$

For each c_{ELF}, the observed risk of liver-related events for each ELF cutoff was calculated as the proportion of participants at or above the cutoff who experienced an event, as follows:

$$\hat{p}_{ELF} = \frac{k_{ELF}}{n_{ELF}}$$

where k_{ELF} is the number participants with an ELF value ≥ c_{ELF} who experienced an LRE, and n_{ELF} is the number participants with an ELF value ≥ c_{ELF}.

Exact binomial 95% confidence intervals (CIs) were calculated using the binom.confint() function with the “exact” method.

<u>Step 2: Generate Candidate VCTE Thresholds</u>

All unique observed baseline VCTE values for use as candidate thresholds were generated by using the R function sort(unique()) function with decreasing = TRUE, and were defined by:

$$T_{VCTE} = \{VCTE_1, VCTE_2 \dots VCTE_n\}$$

<u>Step 3: Calculate Observed LRE Risk at Each Candidate VCTE Threshold</u>

Analogous to Step 1, the observed risk of liver-related events for each candidate VCTE threshold was calculated as the proportion of participants at or above the threshold who experienced an event, as follows

$$\hat{p}_{VCTE} = \frac{k_{VCTE}}{n_{VCTE}}$$

Step 4: Map ELF Cutoffs to VCTE Threshold

For each c_{ELF}, the equivalent VCTE threshold was defined as the value where the observed LRE risk is closest that for ELF.

$$t^*_{VCTE} = \underset{t \in T_{VCTE}}{\operatorname{argmin}} | \hat{p}_{VCTE} - \hat{p}_{ELF} |$$

Step 5: Map Empirically Derived VCTE Cutoffs to Nearest Established Cutoff

To enhance clinical interpretability, each derived t^*_{VCTE} was mapped to the nearest established VCTE cutoff in the set of 5.0, 8.0, 10.0, 12.0, 15.0, 20.0, 25.0, and 30 kPa. (NOTE: The previously derived 30.7 kPa prognostic cutoff for VCTE was rounded down to 30.0 kPa to better reflect the rule-of-five framework established by Baveno VII.)[1,2]

Observed risks were recalculated for these mapped thresholds with 95% CIs derived using the exact binomial approach.

Table S4. Corresponding ELF thresholds to established VCTE cutoffs with respect to risk of liver-related events

VCTE		ELF			
**Established VCTE Cutoff (kPa)**	**LRE Risk (95% CI)**	**Empirically Derived ELF Cutoff**	**LRE Risk (95% CI)**	**Nearest ELF Cutoff in Clinical Use**	**LRE Risk (95% CI)**
≥5.0	3.8% (2.9%, 4.9%)	≥8.30	3.8% (2.9%, 4.9%)	≥8.30	3.8% (2.9%, 4.9%)
≥8.0	4.0% (3.0%, 5.2%)	≥8.82	4.0% (3.0%, 5.2%)	≥9.00	4.1% (3.1%, 5.3%)
≥10.0	4.2% (3.1%, 5.4%)	≥9.14	4.2% (3.2%, 5.4%)		
≥12.0	4.8% (3.6%, 6.2%)	≥9.64	4.8% (3.6%, 6.2%)	≥9.80	4.9% (3.7%, 6.4%)
≥15.0	5.7% (4.3%, 7.5%)	≥10.09	5.7% (4.3%, 7.5%)		
≥20.0	7.3% (5.4%, 9.7%)	≥10.54	7.3% (5.4%, 9.7%)	≥10.50	7.2% (5.3%, 9.4%)
≥25.0	9.9% (7.1%, 13.4%)	≥10.98	9.9% (7.2%, 13.2%)	≥11.30	12.8% (9.1%, 17.3%)

LRE: liver-related event

Table S5. Corresponding FIB-4 thresholds to established ELF cutoffs with respect to risk of liver-related events

ELF		FIB-4			
**Established ELF Cutoff**	**LRE Risk (95% CI)**	**Empirically Derived Cutoff**	**LRE Risk (95% CI)**	**Nearest FIB-4 Cutoff in Clinical Use (kPa)**	**LRE Risk (95% CI)**
≥7.70	3.8% (2.9%, 4.9%)	≥0.50	3.8% (2.9%, 4.9%)	≥1.00	4.1% (3.1%, 5.3%)
≥8.30	3.8% (2.9%, 4.9%)	≥0.60	3.8% (2.9%, 4.9%)		
≥9.00	4.1% (3.1%, 5.3%)	≥1.00	4.1% (3.1%, 5.3%)		
≥9.80	4.9% (3.7%, 6.4%)	≥1.40	4.9% (3.7%, 6.4%)	≥1.30[a]	4.6% (3.5%, 6.0%)
≥10.50	7.2% (5.3%, 9.4%)	≥2.20	7.2% (5.4%, 9.4%)	≥2.00[b]	6.2% (4.7%, 8.1%)
≥11.30	12.8% (9.1%, 17.3%)	≥3.60	12.7% (9.1%, 17.0%)	≥3.48	11.9% (8.5%, 15.9%)
≥12.00	21.3% (13.5%, 30.9%)	≥6.30	21.3% (11.9%, 33.7%)	N/A	N/A
≥12.80	47.4% (24.4%, 71.1%)	≥13.60	50.0% (1.3%, 98.7%)	N/A	N/A

LRE: liver-related event

[a] The LRE risk at FIB-4 ≥1.67 is 5.5% (4.2%, 7.1%).

[b] The LRE risk at FIB-4 ≥2.67 is 8.3% (6.1%, 11.0%).

Table S6. Risk of LREs at multiple different thresholds

Test / Combination[a]	Total Patients	Patients with Events	Clinical Outcome Risk (95% CI)	Likelihood Ratio (95% CI)	LRE Incidence per 1000 PY (95% CI)
**Histology**					
NASH CRN F3	660	7	1.1% (0.4%, 2.2%)	0.27 (0.14, 0.55)	7.7 (2.2, 14.4)
NASH CRN F4	853	50	5.9% (4.4%, 7.7%)	1.59 (1.43, 1.77)	45.2 (33.5, 57.9)
Overall (F3 & F4)	1513	57	3.8% (2.9%, 4,9%)	---	28.4 (21.4, 35.8)
**ELF Alone**					
ELF <10.39[b]	793	9	1.1% (0.5%, 2.1%)	0.29 (0.16, 0.54)	8.2 (3.6, 13.6)
ELF ≥9.80	1076	53	4.9% (3.7%, 6.4%)	1.33 (1.22, 1.43)	37.8 (27.8, 48.5)
ELF ≥10.14[c]	870	51	5.9% (4.4%, 7.6%)	1.59 (1.44, 1.76)	45.8 (34.1, 58.3)
ELF ≥10.50	654	47	7.2% (5.3%, 9.4%)	1.98 (1.73, 2.27)	57.4 (41.5, 74.5)
ELF ≥11.30	274	35	12.8% (9.1%, 17.3%)	3.75 (2.96, 4.74)	105.9 (72.6, 142.3)
**VCTE Alone**					
VCTE <14.0 kPa[b]	563	6	1.1% (0.4%, 2.3%)	0.28 (0.13, 0.59)	7.6 (2.5, 14.0)
VCTE ≥12.0 kPa	1104	53	4.8% (3.6%, 6.2%)	1.29 (1.19, 1.39)	36.9 (27.1, 47.3)
VCTE ≥15.0 kPa	875	50	5.7% (4.3%, 7.5%)	1.55 (1.39, 1.73)	44.6 (33.0, 57.0)
VCTE ≥15.5 kPa[c]	836	49	5.9% (4.4%, 7.7%)	1.59 (1.42, 1.79)	45.9 (33.7, 59.0)
VCTE ≥20.0 kPa	600	44	7.3% (5.4%, 9.7%)	2.02 (1.73, 2.36)	57.9 (40.8, 75.1)
VCTE ≥25.0 kPa	382	38	9.9% (7.1%, 13.4%)	2.83 (2.30, 3.47)	80.3 (55.0, 107.8)
VCTE ≥30.0 kPa	253	34	13.4% (9.5%, 18.3%)	3.97 (3.11, 5.08)	108.7 (73.5, 147.0)
**ELF and VCTE Combination**					
ELF ≥9.80 & VCTE ≥12.0 kPa	871	51	5.9% (4.4%, 7.6%)	1.59 (1.44, 1.76)	45.7 (34.0, 58.2)
ELF ≥10.50 & VCTE ≥15.0 kPa	504	42	8.3% (6.1%, 11.1%)	2.33 (1.96, 2.76)	67.6 (48.3, 88.6)
ELF ≥10.50 & VCTE ≥20.0 kPa	379	38	10.0% (7.2%, 13.5%)	2.85 (2.32, 3.50)	82.0 (56.1, 110.1)
ELF ≥11.30 & VCTE ≥20.0 kPa	200	31	15.5% (10.8%, 21.3%)	4.69 (3.56, 6.19)	131.5 (89.1, 178.1)
ELF ≥11.30 & VCTE ≥25.0 kPa	157	29	18.5% (12.7%, 25.4%)	5.80 (4.28, 7.85)	160.6 (105.2, 221.5)
ELF ≥11.30 & VCTE ≥30.0 kPa	125	27	21.6% (14.7%, 29.8%)	7.05 (5.05, 9.84)	188.2 (118.5, 264.9)

[a] Rows shaded in blue and green demonstrate liver-related event risk comparable to NASH CRN stage F3 and F4, respectively across multiple risk metrics.

[b] Empirically derived ELF (<10.39) and VCTE (<14.0 kPa) thresholds with equivalent risk as NASH CRN stage F3.

[c] Empirically derived ELF (≥10.14) and VCTE (≥15.5 kPa) thresholds with equivalent risk as NASH CRN stage F4.

Table S7.
Comparison of liver-related event risks at 18 months for ELF and VCTE at established thresholds

ELF		VCTE	
**ELF Cutoff**	**Clinical Outcome Risk (95% CI)**	**VCTE Cutoff (kPa)**	**Clinical Outcome Risk (95% CI)**
≥8.30	12.1% (9.3%, 15.4%)	≥5.0	12.1% (9.3%, 15.4%)
≥9.00	12.9% (9.9%, 16.4%)	≥8.0	13.0% (10.0%, 16.5%)
		≥10.0	13.6% (10.4%, 17.4%)
≥9.80	15.8% (12.0%, 20.1%)	≥12.0	16.0% (12.2%, 20.4%)
≥10.50	24.6% (18.7%, 31.3%)	≥20.0	24.7% (18.6%, 31.7%)
≥11.30	37.6% (27.8%, 48.3%)	≥30.0	37.0% (27.1%, 47.7%)
≥12.00	62.5% (43.7%, 78.9%)	N/A	N/A
≥12.80	75.0% (42.8%, 94.5%)	N/A	N/A

18-month event risk was calculated with the following groups:
Patients with Clinical Outcome: number of patients with LRE ≤18 months
Patients without Clinical Outcome: number of patients without LRE and follow-up >18 months

4. NIT Combinations

Table S8. Laboratory characteristics of patients stratified by concordance / discordance of ELF and VCTE measurements

Interpretation	ELF and VCTE Relative to Respective Cutoffs	Platelets [count]		AST [IU/L]		ALT [IU/L]		GGT [IU/L]		ALP [IU/L]		Bilirubin [mg/dL]	
		Median (IQR)	Wilcoxon Rank Sum p-value	Median (IQR)	Wilcoxon Rank Sum p-value	Median (IQR)	Wilcoxon Rank Sum p-value	Median (IQR)	Wilcoxon Rank Sum p-value	Median (IQR)	Wilcoxon Rank Sum p-value	Median (IQR)	Wilcoxon Rank Sum p-value
Agree Below (↓)	ELF <11.30 VCTE <30.0 kPa	190.0 (147.0, 244.0)		41.5 (31.5, 55.7)		45.5 (32.0, 68.0)		58.0 (38.0, 101.0)		81.0 (67.0, 101.0)		0.6 (0.4, 0.8)	
Disagree	ELF <11.30 VCTE ≥30.0 kPa	153.0 (118.0, 219.8)	0.0823	47.2 (32.4, 62.2)	<0.0001	39.8 (29.9, 63.0)	0.0002	102.0 (62.5, 164.0)	0.9084	92.5 (76.8, 118.2)	0.0328	0.6 (0.4, 0.8)	0.3469
	ELF ≥11.30 VCTE <30.0 kPa	143.0 (110.0, 181.0)		61.0 (46.0, 79.0)		54.5 (38.0, 79.5)		98.0 (58.0, 185.0)		101.0 (82.0, 124.0)		0.6 (0.5, 0.9)	
Agree Above (↑)	ELF ≥11.30 VCTE ≥30.0 kPa	118.0 (97.0, 169.0)		59.0 (46.0, 82.0)		41.0 (32.0, 59.0)		126.0 (77.0, 227.0)		123.0 (90.0, 152.0)		0.8 (0.5, 1.1)	

Table S9. Risk of liver-related clinical outcomes stratified by ELF and VCTE combinations (alternate cutoff pairs)

Interpretation	ELF and VCTE Relative to Respective Cutoffs	Total Patients	Patients with Events	LRE Risk (95% CI)[a]	Interval Likelihood Ratio (95% CI)	LRE Incidence per 1000 PY (95% CI)	Cox Proportional Hazard Ratio (95% CI) [P-Value]
Agree Below (↓)	ELF <10.50; VCTE <15.0 kPa	490	2	0.4% (0.0%, 1.5%)	0.10 (0.03, 0.41)	2.9 (0.0, 7.2)	1.00 (Reference)
Disagree	ELF <10.50; VCTE ≥15.0 kPa	371	8	2.2% (0.9%, 4.2%)	0.56 (0.29, 1.08)	16.0 (6.0, 27.9)	---
	ELF ≥10.50; VCTE <15.0 kPa	150	5	3.3% (1.1%, 7.6%)	0.88 (0.38, 2.07)	25.3 (5.1, 50.5)	---
	Total	521	13	2.5% (1.3%, 4.2%)	0.65 (0.40, 1.06)	18.6 (8.6, 30.0)	6.35 (1.43, 28.15) [p=0.0149]
Agree Above (↑)	ELF ≥10.50; VCTE ≥15.0 kPa	504	42	8.3% (6.1%, 11.1%)	2.33 (1.96, 2.76)	67.6 (48.3, 88.6)	23.02 (5.57, 95.11) [p<0.0001]
Agree Below (↓)	ELF <11.30; VCTE <20.0 kPa	841	9	1.1% (0.5%, 2.0%)	0.28 (0.15, 0.50)	7.8 (3.5, 12.9)	1.00 (Reference)
Disagree	ELF <11.30; VCTE ≥20.0 kPa	400	13	3.2% (1.7%, 5.5%)	0.86 (0.53, 1.40)	24.8 (11.5, 40.1)	---
	ELF ≥11.30; VCTE <20.0 kPa	74	4	5.4% (1.5%, 13.3%)	1.46 (0.55, 3.86)	42.3 (10.6, 84.6)	---
	Total	474	17	3.6% (2.1%, 5.7%)	0.95 (0.63, 1.43)	27.5 (14.6, 42.1)	3.50 (1.56, 7.86) [p=0.0024]
Agree Above (↑)	ELF ≥11.30; VCTE ≥20.0 kPa	200	31	15.5% (10.8%, 21.3%)	4.69 (3.56, 6.19)	131.5 (89.1, 178.1)	17.07 (8.12, 35.88) [p<0.0001]
Agree Below (↓)	ELF <11.30; VCTE <25.0 kPa	1016	13	1.3% (0.7%, 2.2%)	0.33 (0.21, 0.54)	9.4 (4.3, 15.1)	1.00 (Reference)
Disagree	ELF <11.30; VCTE ≥25.0 kPa	225	9	4.0% (1.8%, 7.5%)	1.07 (0.58, 1.97)	30.8 (13.7, 51.3)	---
	ELF ≥11.30; VCTE <25.0 kPa	117	6	5.1% (1.9%, 10.8%)	1.38 (0.64, 3.01)	40.1 (13.4, 73.5)	---
	Total	342	15	4.4% (2.5%, 7.1%)	1.17 (0.75, 1.83)	33.9 (18.1, 52.0)	3.64 (1.73, 7.65) [p=0.0007]
Agree Above (↑)	ELF ≥11.30; VCTE ≥25.0 kPa	157	29	18.5% (12.7%, 25.4%)	5.80 (4.28, 7.85)	160.6 (105.2, 221.5)	17.50 (9.09, 33.69) [p<0.0001]

[a] Fisher's exact test was used to compare liver-related event rates between discordant groups (VCTE < cutoff & ELF ≥ cutoff vs. ELF < cutoff & VCTE ≥ cutoff). P-values for the respective ELF/VCTE cutoff pairings were: 10.50/15.0 kPa (p=0.5350), 11.30/20.0 kPa (p=0.3191), and 11.30/25.0 kPa (p=0.5925).

Table S10. Risk of liver-related clinical outcomes stratified by ELF, VCTE and FIB-4 combinations

Interpretation[a]	NIT Cutoffs	Total Patients	Patients with Events	LRE Risk (95% CI)[a]	Interval Likelihood Ratio (95% CI)	LRE Incidence per 1000 PY (95% CI)	Cox Proportional Hazard Ratio (95% CI) [P-Value]
All Below (↓)	ELF: 9.80<br>VCTE: 12.0 kPa<br>FIB-4: 1.30	96	0	0.0% (0.0%, 3.8%)	0.00	0.0	0.00
Disagree		618	6	1.0% (0.4%, 2.1%)	0.25 (0.12, 0.54)	6.9 (2.3, 12.7)	1.00 (Reference)
All Above (↑)		801	51	6.4% (4.8%, 8.3%)	1.74 (1.57, 1.93)	50.1 (37.3, 63.8)	6.95 (2.98, 16.19) [p<0.0001]
All Below (↓)	ELF: 10.50<br>VCTE: 20.0 kPa<br>FIB-4: 2.67	551	1	0.2% (0.0%, 1.0%)	0.05 (0.01, 0.32)	1.3 (0.0, 3.9)	1.00 (Reference)
Disagree		698	24	3.4% (2.2%, 5.1%)	0.91 (0.67, 1.24)	26.2 (16.4, 37.1)	20.27 (2.74, 149.86) [p=0.0032]
All Above (↑)		266	32	12.0% (8.4%, 16.6%)	3.50 (2.70, 4.53)	100.3 (65.8, 137.9)	77.34 (10.56, 566.21) [p<0.0001]
All Below (↓)	ELF: 11.30<br>VCTE: 30.0 kPa<br>FIB-4: 3.48	988	7	0.7% (0.3%, 1.5%)	0.18 (0.09, 0.37)	5.2 (1.5, 9.6)	1.00 (Reference)
Disagree		443	28	6.3% (4.2%, 9.0%)	1.73 (1.31, 2.28)	49.6 (31.9, 69.1)	9.72 (4.24, 22.26) [p<0.0001]
All Above (↑)		84	22	26.2% (17.2%, 36.9%)	9.08 (6.03, 13.65)	243.8 (144.1, 354.7)	48.16 (20.55, 112.87) [p<0.0001]

[a] All Below: all 3 NITs < respective cutoffs; Disagree: 1 or 2 NITs < respective cutoffs; All Above: all 3 NITs ≥ respective cutoffs

Fig. S2.

Heatmaps of observed liver-related event risk by baseline ELF and VCTE category

A

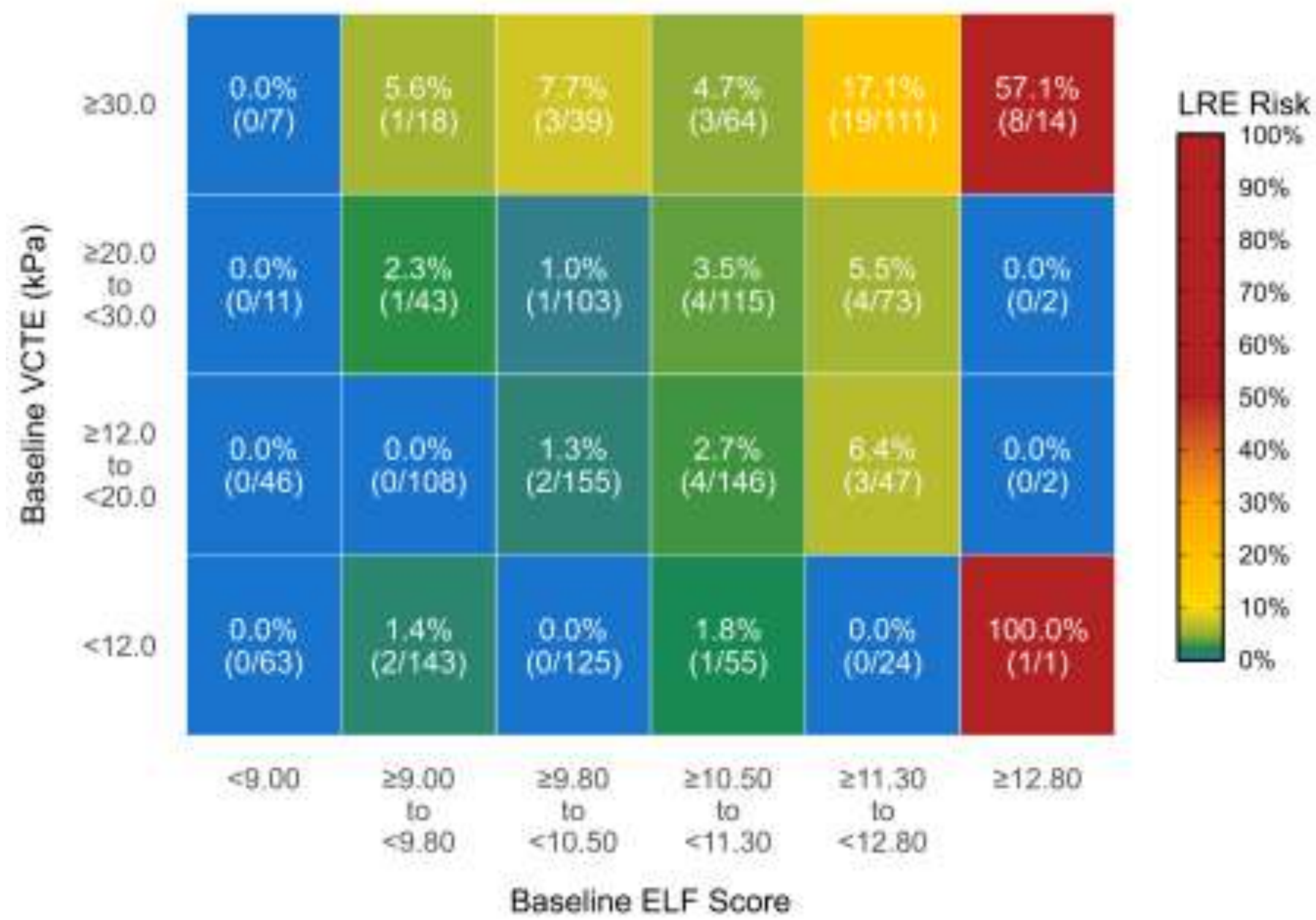

B

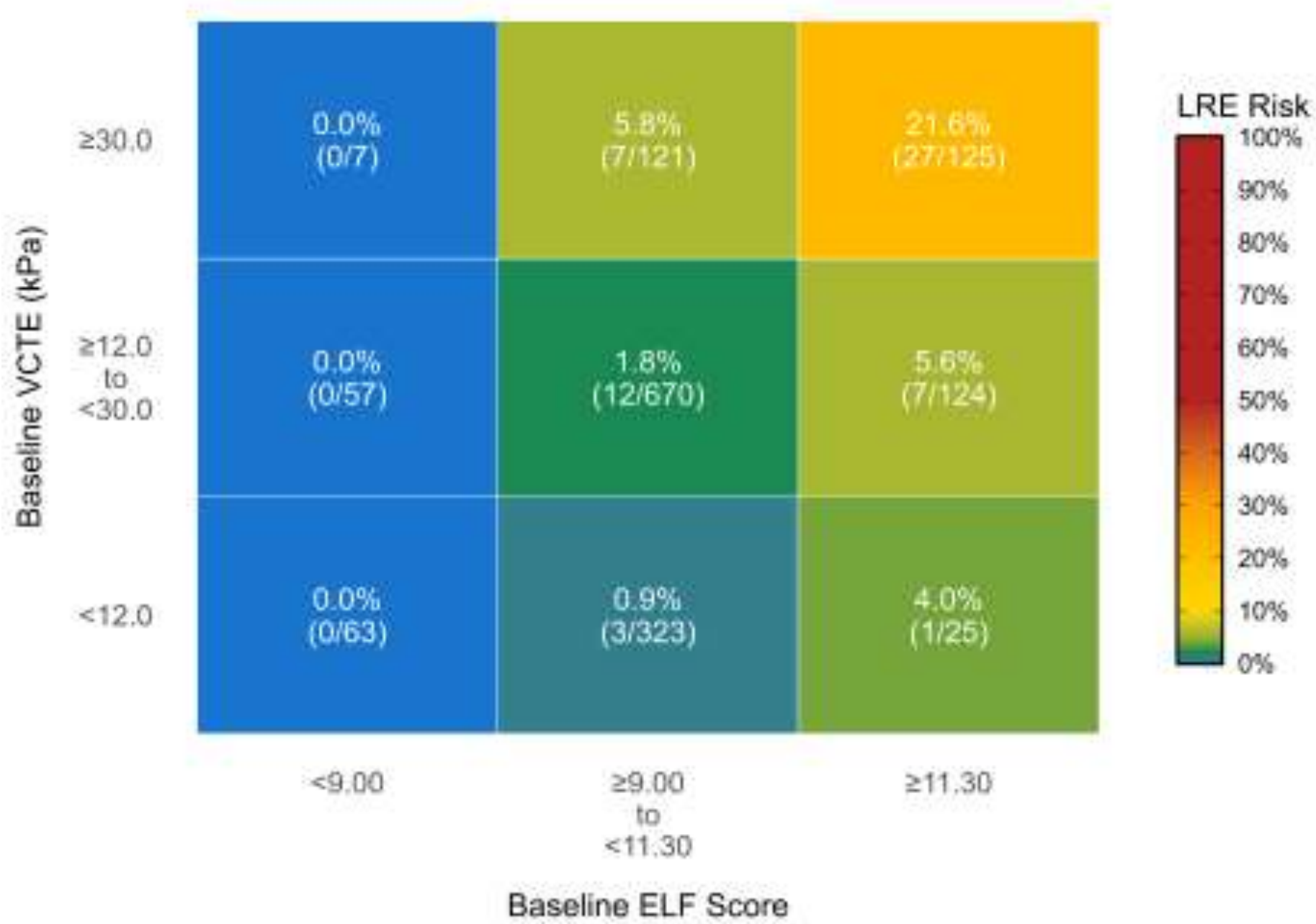

Cells display the proportion of patients experiencing a liver-related event within each ELF/VCTE stratum, with denominators shown in parentheses. Colour intensity reflects increasing event risk, but does not imply statistical significance between strata. (A) Full stratification of baseline ELF and VCTE categories. (B) Reduced stratification combining adjacent strata with similar event rates and limited sample sizes for display purposes.

Fig. S3.
Kaplan-Meier curves for event-free probability of LREs (stratification by baseline NASH CRN stage)

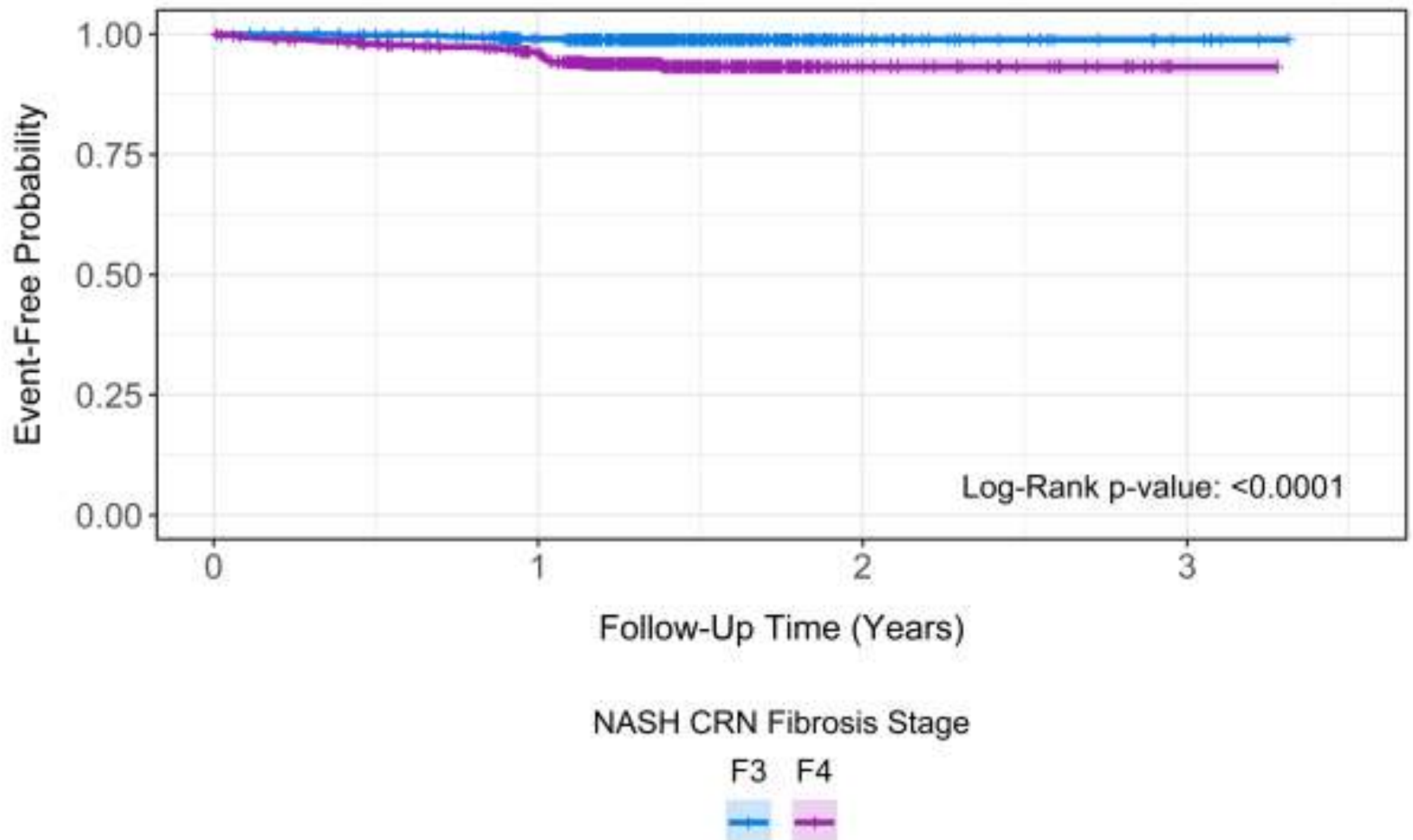

At Risk (Events)

F3	660 (0)	537 (6)	32 (7)	6 (7)
F4	853 (0)	689 (33)	29 (50)	1 (50)

Fig. S4.

Kaplan-Meier curves for event-free probability of LREs based on alignment of ELF, VCTE and FIB-4

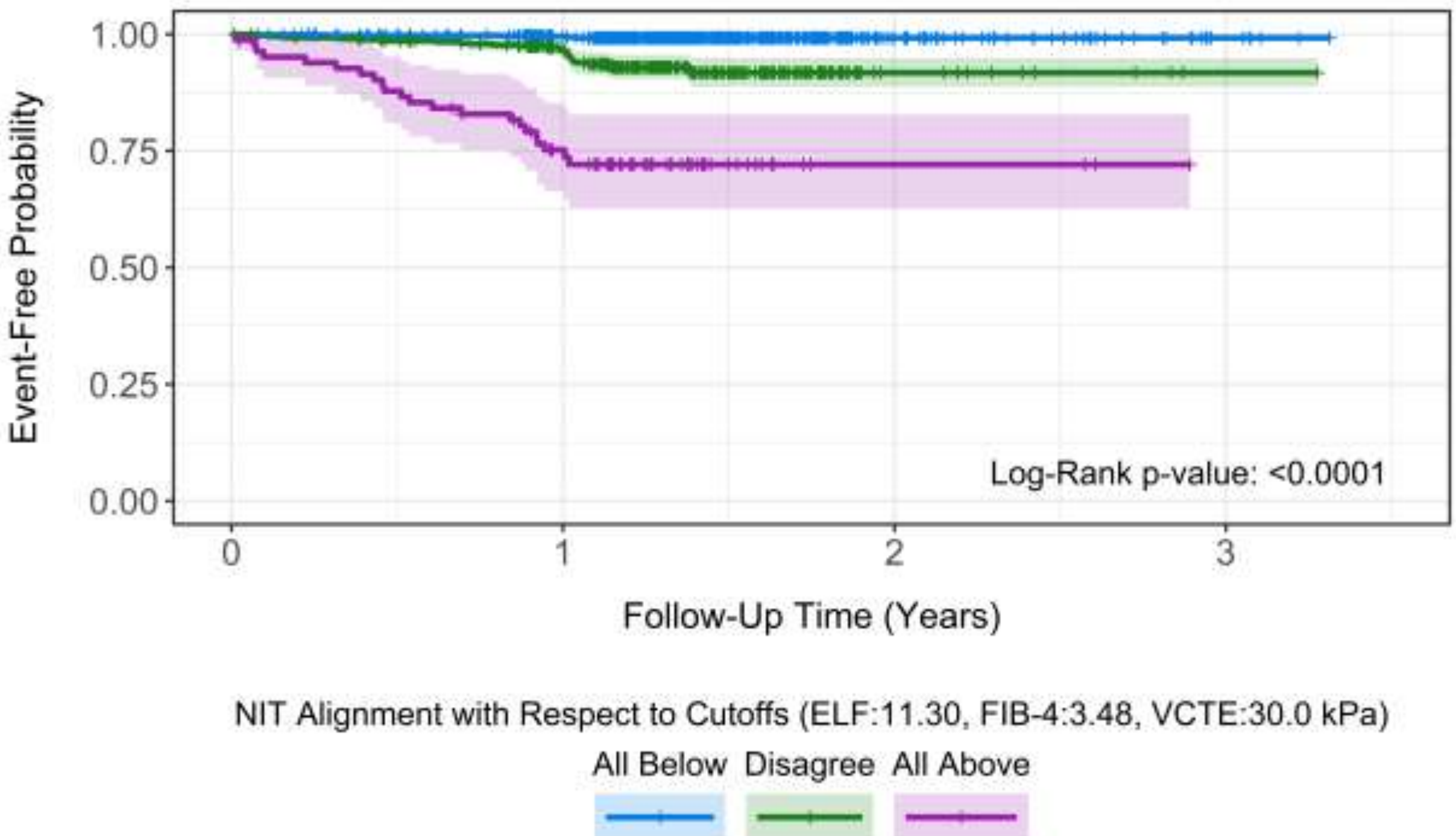

At Risk (Events)

All Below	988 (0)	858 (5)	48 (7)	6 (7)
Disagree	443 (0)	321 (14)	11 (28)	1 (28)
All Above	84 (0)	48 (20)	3 (22)	0 (22)

Risk stratification based on NIT alignment with respect to cutoffs (ELF:11.30, VCTE:30.0 kPa, FIB-4: 3.48). “All Below” (Blue): ELF <11.30 & VCTE <30.0 kPa & FIB-4 <3.48 ; “Disagree” (Green): ELF <11.30 & VCTE <30.0 kPa & FIB-4 ≥3.48 OR ELF <11.30 & VCTE ≥30.0 kPa & FIB-4 <3.48 OR ELF ≥11.30 & VCTE <30.0 kPa & FIB-4 <3.48 OR ELF ≥11.30 & VCTE ≥30.0 kPa & FIB-4 <3.48 OR ELF ≥11.30 & VCTE <30.0 kPa & FIB-4 ≥3.48 OR ELF <11.30 & VCTE ≥30.0 kPa & FIB-4 ≥3.48; “All Agree” (Purple): ELF ≥11.30 & VCTE ≥30.0 kPa & FIB-4 ≥3.48

5. Model Comparisons

Table S11. Summary statistics from Cox regressions for risk-stratification models of ELF and VCTE alone and in combination

Model	Low Stratum	Mid Stratum	High Stratum	C-Index	logLik	AIC	BIC
VCTE Alone<br>12.0 / 20.0 kPa	<12.0 kPa	≥12.0 to <20.0 kPa	≥20.0 kPa	0.708	-390.88	785.75	789.84
ELF Alone<br>9.80 / 11.30	<9.80	≥9.80 to <11.30	≥11.30	0.758	-379.01	762.03	766.12
ELF/VCTE Combo 1<br>10.50 / 15.0 kPa	ELF <10.50 &<br>VCTE <15.0 kPa	Disagree[a]	ELF ≥10.50 &<br>VCTE ≥15.0 kPa	0.750	-383.67	771.34	775.43
ELF/VCTE Combo 2<br>10.50 / 20.0 kPa	ELF <10.50 &<br>VCTE <20.0 kPa	Disagree	ELF ≥10.50 &<br>VCTE ≥20.0 kPa	0.770	-380.35	764.70	768.78
ELF/VCTE Combo 3<br>11.30 / 20.0 kPa	ELF <11.30 &<br>VCTE <20.0 kPa	Disagree	ELF ≥11.30 &<br>VCTE ≥20.0 kPa	0.781	-374.25	752.5	756.59
ELF/VCTE Combo 4<br>11.30 / 25.0 kPa	ELF <11.30 &<br>VCTE <25.0 kPa	Disagree	ELF ≥11.30 &<br>VCTE ≥25.0 kPa	0.783	-370.84	745.68	749.77
ELF/VCTE Combo 5<br>11.30 / 30.0 kPa	ELF <11.30 &<br>VCTE <30.0 kPa	Disagree	ELF ≥11.30 &<br>VCTE ≥30.0 kPa	0.786	-368.21	740.42	744.51

[a] “Disagree” for the combination models refers to one NIT < cutoff and the other NIT ≥ cutoff

Fig. S5.
Time-dependent area under the receiver operator characteristic curve by baseline ELF, VCTE or ELF/VCTE combination

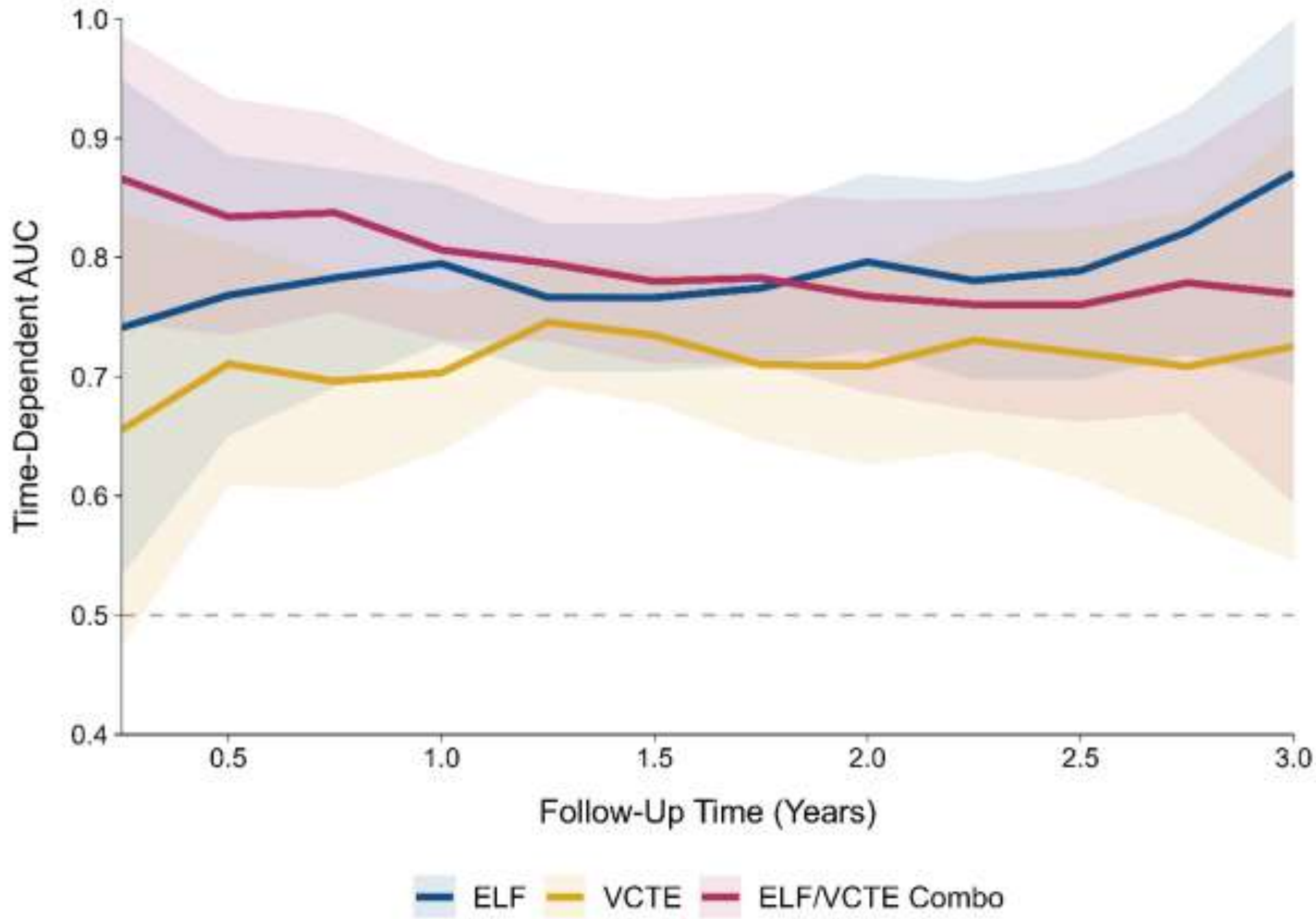

ELF (stratified by cutoffs at 9.80 and 11.30), VCTE (stratified by cutoffs at 12.0 and 20.0 kPa), or ELF and VCTE combination (agreement-based stratification using 11.30 and 30.0 kPa cutoffs).

Table S12.
Time-dependent area under the receiver operator characteristic curve (AUC(t)) by baseline ELF and VCTE alone and combination

Time (Years)	AUC(t) (95% CI) for Each Model			P-Value for Comparisons		
	ELF	VCTE	ELF/VCTE Combination	ELF vs VCTE	ELF/VCTE vs ELF	ELF/VCTE vs VCTE
0.25	0.741 (0.533, 0.950)	0.656 (0.474, 0.837)	0.866 (0.746, 0.986)	0.4508	0.0767	0.0004
0.50	0.768 (0.650, 0.886)	0.711 (0.609, 0.813)	0.834 (0.735, 0.933)	0.3561	0.1364	0.0099
0.75	0.783 (0.691, 0.875)	0.696 (0.606, 0.787)	0.838 (0.755, 0.921)	0.0930	0.1170	0.0001
1.00	0.795 (0.728, 0.862)	0.703 (0.637, 0.770)	0.806 (0.730, 0.882)	0.0097	0.6613	0.0010
1.25	0.766 (0.704, 0.829)	0.746 (0.692, 0.800)	0.795 (0.730, 0.861)	0.5294	0.2209	0.1057
1.50	0.766 (0.704, 0.829)	0.735 (0.677, 0.793)	0.780 (0.711, 0.849)	0.3648	0.5748	0.1589
1.75	0.774 (0.709, 0.840)	0.710 (0.645, 0.775)	0.783 (0.711, 0.854)	0.0888	0.7403	0.0354
2.00	0.796 (0.722, 0.870)	0.709 (0.626, 0.792)	0.767 (0.687, 0.848)	0.0443	0.2807	0.1462
2.25	0.781 (0.697, 0.864)	0.731 (0.638, 0.823)	0.760 (0.672, 0.849)	0.2969	0.4675	0.4840
2.50	0.789 (0.697, 0.881)	0.720 (0.615, 0.825)	0.760 (0.662, 0.858)	0.1870	0.3179	0.3982
2.75	0.821 (0.718, 0.925)	0.709 (0.580, 0.837)	0.779 (0.670, 0.888)	0.0801	0.1798	0.2436
3.00	0.871 (0.695, 1.000)	0.725 (0.545, 0.905)	0.769 (0.594, 0.945)	0.0729	0.0001	0.5767

ELF Strata
Low: ELF <9.80
Mid: ELF ≥9.80 to <11.30
High: ELF ≥11.30

VCTE Strata
Low: VCTE <12.0 kPa
Mid: VCTE ≥12.0 to <20.0 kPa
High: VCTE ≥20.0 kPa

ELF + VCTE Combination Strata
Agree ↓: ELF <11.30 & VCTE <30.0 kPa
Disagree: ELF ≥11.30 & VCTE <30.0 kPa; OR ELF <11.30 & VCTE ≥30.0 kPa
Agree ↑: ELF ≥11.30 & VCTE ≥30.0 kPa

6. Sensitivity Analyses

Table S13.
Baseline ELF/VCTE combination to predict liver-related events (sensitivity analysis: treatment vs placebo)

Baseline ELF/VCTE Strata	Study Arm	n	Events	Event Rate (95% CI)	P-value[a]
Agree Above (↑): ELF ≥11.30 & VCTE ≥30.0 kPa	Treatment	106	24	22.6% (15.1%, 31.8%)	0.7625
	Placebo	19	3	15.8% (3.4%, 39.6%)	
	Pooled	125	27	21.6% (14.7%, 29.8%)	---
Disagree: ELF ≥11.30 & VCTE <30.0 kPa OR ELF <11.30 & VCTE ≥30.0 kPa	Treatment	219	10	4.6% (2.2%, 8.2%)	0.3530
	Placebo	58	5	8.6% (2.9%, 19.0%)	
	Pooled	277	15	5.4% (3.1%, 8.8%)	---
Agree Below (↓): ELF <11.30 & VCTE <30.0 kPa	Treatment	866	9	1.0% (0.5%, 2.0%)	0.1148
	Placebo	247	6	2.4% (0.9%, 5.2%)	
	Pooled	1113	15	1.3% (0.8%, 2.2%)	---

[a] P-values determined by Fisher's exact 2-sided test.

Table S14.
Baseline ELF/VCTE combination to predict liver-related events (sensitivity analysis: diabetes status)

Baseline ELF/VCTE Strata	Diabetes	n	Events	Event Rate (95% CI)	P-value[a]
Agree Above (↑): ELF ≥11.30 & VCTE ≥30.0 kPa	Present	95	17	17.9% (10.8%, 27.1%)	0.0812
	Absent	30	10	33.3% (17.3%, 52.8%)	
	Pooled	125	27	21.6% (14.7%, 29.8%)	---
Disagree: ELF ≥11.30 & VCTE <30.0 kPa OR ELF <11.30 & VCTE ≥30.0 kPa	Present	191	9	4.7% (2.2%, 8.8%)	0.5664
	Absent	86	6	7.0% (2.6%, 14.6%)	
	Pooled	277	15	5.4% (3.1%, 8.8%)	---
Agree Below (↓): ELF <11.30 & VCTE <30.0 kPa	Present	811	12	1.5% (0.8%, 2.6%)	0.7710
	Absent	302	3	1.0% (0.2%, 2.9%)	
	Pooled	1113	15	1.3% (0.8%, 2.2%)	---

[a] P-values determined by Fisher's exact 2-sided test.

Table S15.
Baseline ELF/VCTE combination to predict liver-related events (sensitivity analysis: age)

Baseline ELF/VCTE Strata	Age	n	Events	Event Rate (95% CI)	P-value[a]
Agree Above (↑): ELF ≥11.30 & VCTE ≥30.0 kPa	<60	61	9	14.8% (7.0%, 26.2%)	0.0839
	≥60	64	18	28.1% (17.6%, 40.8%)	
	Pooled	125	27	21.6% (14.7%, 29.8%)	---
Disagree: ELF ≥11.30 & VCTE <30.0 kPa OR ELF <11.30 & VCTE ≥30.0 kPa	<60	144	6	4.2% (1.5%, 8.8%)	0.4287
	≥60	133	9	6.8% (3.1%, 12.5%)	
	Pooled	277	15	5.4% (3.1%, 8.8%)	---
Agree Below (↓): ELF <11.30 & VCTE <30.0 kPa	<60	608	6	1.0% (0.4%, 2.1%)	0.3012
	≥60	505	9	1.8% (0.8%, 3.4%)	
	Pooled	1113	15	1.3% (0.8%, 2.2%)	---

[a] P-values determined by Fisher's exact 2-sided test.

7. Diagnostic Analyses

Comparability of diagnostic performance of the prognostically-matched NIT cutoffs was evaluated. Data were pooled from 1443 participants in the following 6 clinical trials: STELLAR-3, STELLAR-4, GS-US-321-0105, GS-US-321-0106, ATLAS, and NASH-CX (Fig. S6). Samples were included in the analysis which had complete measurements at 1-year for NASH CRN fibrosis stage, VCTE, and laboratory tests including ELF (N = 1443). Additionally, an expanded dataset with complete cases for ELF and NASH CRN stage (N = 2390) was also used for ELF-only analyses.

The ELF cutoffs used in this comparison were: 8.30, 9.00, 9.80, 10.50, 11.30, and 12.00. The cutoffs for VCTE were 5.0, 10.0, 12.0, 20.0, 30.0, and 50.0 kPa. These thresholds were used individually and in combination as a prognostically-aligned ELF/VCTE pair.

Analyses for the discrimination of significant fibrosis (≥F2), advanced fibrosis (≥F3) and cirrhosis (F4) were evaluated using samples with histological stage (NASH CRN), ELF, VCTE, and FIB-4 measurements. Measurements of histology and NITs were based on the 1-year time point of the respective trials due to limited <F2 samples at baseline. Calculations were performed for each NIT alone and in combination with one another. To assess correlation to histology, sensitivity, specificity, diagnostic accuracy, and associated Clopper-Pearson exact 95% confidence intervals were calculated.

Summaries of the baseline demographics and clinical characteristics in the diagnostic datasets are presented in Table S16.

Results for are presented in Table S17 (≥F2 vs <F2), Table S18 (≥F3 vs <F3), Table S19 (F4 vs <F4), and Table S20 (expanded dataset for ELF).

Fig. S6. Exclusions from primary diagnostic analyses

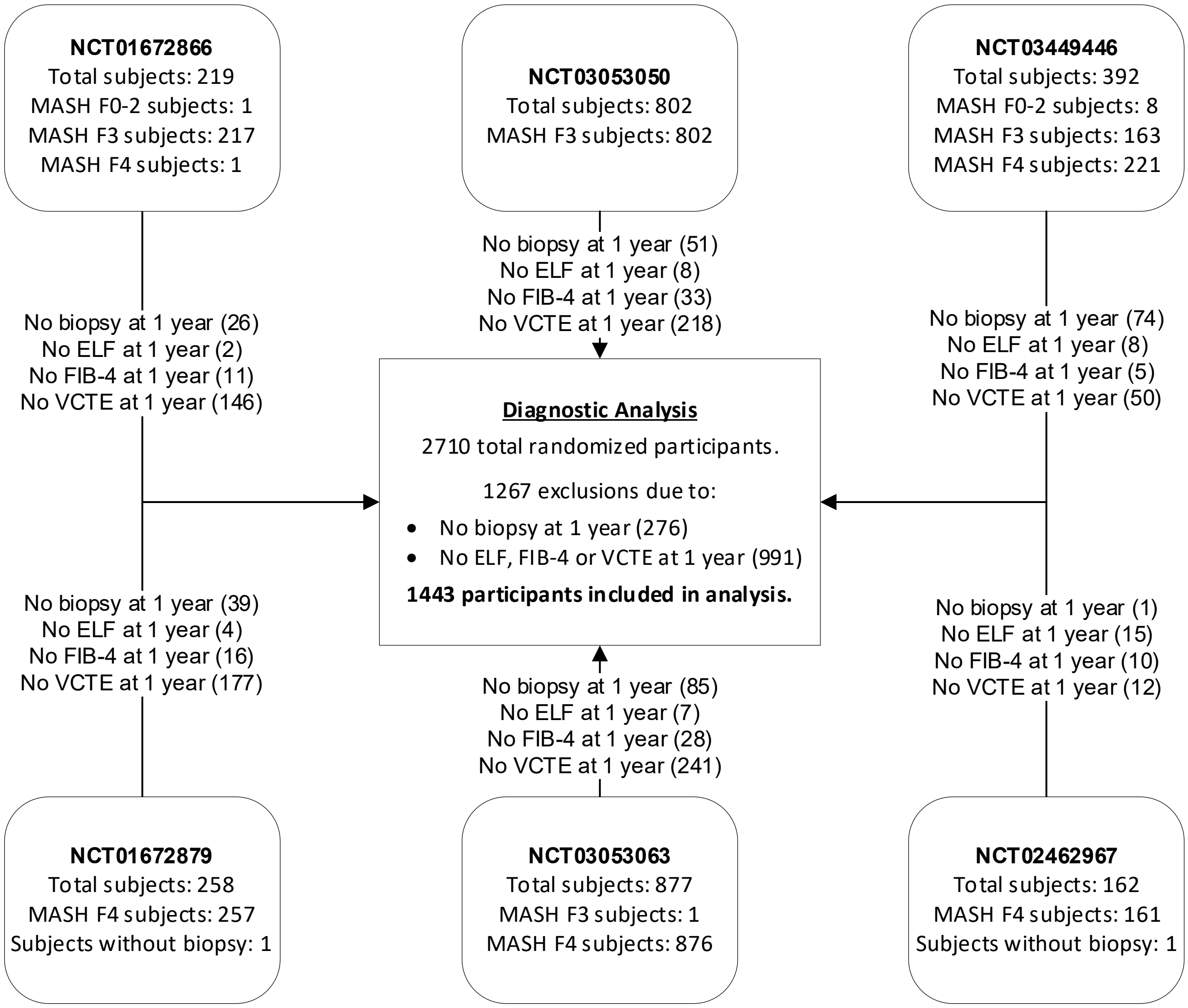

Table S16. Baseline demographics and clinical characteristics of diagnostic datasets

Baseline Parameter	Primary Diagnostic Dataset: Complete Cases for ELF, VCTE, FIB-4, NASH CRN Stage at 1 Year	Expanded Dataset: Complete Cases for ELF and NASH CRN Stage at 1 Year
N	1443	2390
Female (%)	883 (61%)	883 (61%)
Type 2 diabetes present (%)	1047 (73%)	1717 (72%)
Age category (%)		
≤34	18 (1%)	39 (2%)
35-39	31 (2%)	50 (2%)
40-44	64 (4%)	93 (4%)
45-49	133 (9%)	227 (9%)
50-54	190 (13%)	322 (13%)
55-59	312 (22%)	550 (23%)
60-64	316 (22%)	550 (23%)
≥65	379 (26%)	559 (23%)
NASH CRN stage (%)[a]		
F0	4 (0%)	6 (0%)
F1	21 (1%)	38 (1%)
F2	73 (5%)	123 (5%)
F3	586 (41%)	945 (41%)
F4	759 (53%)	1278 (53%)
Median ELF score (IQR)[a]	10.38 (9.70, 11.10)	10.40 (9.69, 11.16)
Median FIB-4 score (IQR)[a]	2.09 (1.40, 3.16)	2.11 (1.40, 3.26)
Median VCTE in kPa (IQR)[a]	15.3 (10.6, 21.8)	15.4 (10.7, 21.8)

[a] Parameters for the diagnostic dataset were obtained at the 1-year timepoint.

Table S17. ELF, VCTE and combinations vs NASH CRN stage at 1-year timepoint (≥F2 vs <F2)

Analysis	ELF Cutoff	VCTE Cutoff (kPa)	TP	FP	FN	TN	Sensitivity (95% CI)	Specificity (95% CI)	Total Agreement (95% CI)
ELF Only	8.30	---	1394	23	24	2	98.3% (97.5%, 98.9%)	8.0% (1.0%, 26.0%)	96.7% (95.7%, 97.6%)
	9.00	---	1303	14	115	11	91.9% (90.3%, 93.3%)	44.0% (24.4%, 65.1%)	91.1% (89.5%, 92.5%)
	9.80	---	1035	6	383	19	73.0% (70.6%, 75.3%)	76.0% (54.9%, 90.6%)	73.0% (70.7%, 75.3%)
	10.50	---	664	1	754	24	46.8% (44.2%, 49.5%)	96.0% (79.6%, 99.9%)	47.7% (45.1%, 50.3%)
	11.30	---	294	0	1124	25	20.7% (18.7%, 22.9%)	100.0% (86.3%, 100.0%)	22.1% (20.0%, 24.3%)
	12.00	---	102	0	1316	25	7.2% (5.9%, 8.7%)	100.0% (86.3%, 100.0%)	8.8% (7.4%, 10.4%)
VCTE Only	---	5.0	1403	21	15	4	98.9% (98.3%, 99.4%)	16.0% (4.5%, 36.1%)	97.5% (96.6%, 98.2%)
	---	8.0[a]	1284	10	134	15	90.6% (88.9%, 92.0%)	60.0% (38.7%, 78.9%)	90.0% (88.4%, 91.5%)
	---	10.0[a]	1139	5	279	20	80.3% (78.2%, 82.4%)	80.0% (59.3%, 93.2%)	80.3% (78.2%, 82.3%)
	---	12.0	969	3	449	22	68.3% (65.8%, 70.8%)	88.0% (68.8%, 97.5%)	68.7% (66.2%, 71.1%)
	---	20.0	474	1	944	24	33.4% (31.0%, 36.0%)	96.0% (79.6%, 99.9%)	34.5% (32.1%, 37.0%)
	---	30.0	192	1	1226	24	13.5% (11.8%, 15.4%)	96.0% (79.6%, 99.9%)	15.0% (13.2%, 16.9%)
	---	50.0	47	0	1371	25	3.3% (2.4%, 4.4%)	100.0% (86.3%, 100.0%)	5.0% (3.9%, 6.2%)
ELF/VCTE Combo	8.30	5.0	1381	20	37	5	97.4% (96.4%, 98.2%)	20.0% (6.8%, 40.7%)	96.0% (94.9%, 97.0%)
	9.00	8.0[a]	1206	5	212	20	85.0% (83.1%, 86.9%)	80.0% (59.3%, 93.2%)	85.0% (83.0%, 86.8%)
	9.00	10.0[a]	1085	1	333	24	76.5% (74.2%, 78.7%)	96.0% (79.6%, 99.9%)	76.9% (74.6%, 79.0%)
	9.80	12.0	806	1	612	24	56.8% (54.2%, 59.4%)	96.0% (79.6%, 99.9%)	57.5% (54.9%, 60.1%)
	10.50	20.0	349	0	1069	25	24.6% (22.4%, 26.9%)	100.0% (86.3%, 100.0%)	25.9% (23.7%, 28.3%)
	11.30	30.0	105	0	1313	25	7.4% (6.1%, 8.9%)	100.0% (86.3%, 100.0%)	9.0% (7.6%, 10.6%)
	12.00	50.0	20	0	1398	25	1.4% (0.9%, 2.2%)	100.0% (86.3%, 100.0%)	3.1% (2.3%, 4.2%)

TP = True Positive, FP = False Positive, FN = False Negative, TN = True Negative

[a] VCTE cutoffs of 8.0 and 10.0 kPa both have prognostic alignment to ELF 9.00.

Table S18. ELF, VCTE and combinations vs NASH CRN stage at 1-year timepoint (≥F3 vs <F3)

Analysis	ELF Cutoff	VCTE Cutoff (kPa)	TP	FP	FN	TN	Sensitivity (95% CI)	Specificity (95% CI)	Total Agreement (95% CI)
**ELF Only**	8.30	---	1329	88	16	10	98.8% (98.1%, 99.3%)	10.2% (5.0%, 18.0%)	92.8% (91.3%, 94.1%)
	9.00	---	1253	64	92	34	93.2% (91.7%, 94.5%)	34.7% (25.4%, 45.0%)	89.2% (87.5%, 90.7%)
	9.80	---	1012	29	333	69	75.2% (72.8%, 77.5%)	70.4% (60.3%, 79.2%)	74.9% (72.6%, 77.1%)
	10.50	---	655	10	690	88	48.7% (46.0%, 51.4%)	89.8% (82.0%, 95.0%)	51.5% (48.9%, 54.1%)
	11.30	---	294	0	1051	98	21.9% (19.7%, 24.2%)	100.0% (96.3%, 100.0%)	27.2% (24.9%, 29.5%)
	12.00	---	102	0	1243	98	7.6% (6.2%, 9.1%)	100.0% (96.3%, 100.0%)	13.9% (12.1%, 15.8%)
**VCTE Only**	---	5.0	1331	93	14	5	99.0% (98.3%, 99.4%)	5.1% (1.7%, 11.5%)	92.6% (91.1%, 93.9%)
	---	8.0[a]	1233	61	112	37	91.7% (90.1%, 93.1%)	37.8% (28.2%, 48.1%)	88.0% (86.2%, 89.6%)
	---	10.0[a]	1102	42	243	56	81.9% (79.8%, 84.0%)	57.1% (46.7%, 67.1%)	80.2% (78.1%, 82.3%)
	---	12.0	950	22	395	76	70.6% (68.1%, 73.1%)	77.6% (68.0%, 85.4%)	71.1% (68.7%, 73.4%)
	---	20.0	471	4	874	94	35.0% (32.5%, 37.6%)	95.9% (89.9%, 98.9%)	39.2% (36.6%, 41.7%)
	---	30.0	190	3	1155	95	14.1% (12.3%, 16.1%)	96.9% (91.3%, 99.4%)	19.8% (17.7%, 21.9%)
	---	50.0	47	0	1298	98	3.5% (2.6%, 4.6%)	100.0% (96.3%, 100.0%)	10.0% (8.5%, 11.7%)
**ELF/VCTE Combo**	8.30	5.0	1317	84	28	14	97.9% (97.0%, 98.6%)	14.3% (8.0%, 22.8%)	92.2% (90.7%, 93.6%)
	9.00	8.0[a]	1168	43	177	55	86.8% (84.9%, 88.6%)	56.1% (45.7%, 66.1%)	84.8% (82.8%, 86.6%)
	9.00	10.0[a]	1057	29	288	69	78.6% (76.3%, 80.8%)	70.4% (60.3%, 79.2%)	78.0% (75.8%, 80.1%)
	9.80	12.0	800	7	545	91	59.5% (56.8%, 62.1%)	92.9% (85.8%, 97.1%)	61.7% (59.2%, 64.3%)
	10.50	20.0	349	0	996	98	25.9% (23.6%, 28.4%)	100.0% (96.3%, 100.0%)	31.0% (28.6%, 33.4%)
	11.30	30.0	105	0	1240	98	7.8% (6.4%, 9.4%)	100.0% (96.3%, 100.0%)	14.1% (12.3%, 16.0%)
	12.00	50.0	20	0	1325	98	1.5% (0.9%, 2.3%)	100.0% (96.3%, 100.0%)	8.2% (6.8%, 9.7%)

TP = True Positive, FP = False Positive, FN = False Negative, TN = True Negative

[a] VCTE cutoffs of 8.0 and 10.0 kPa both have prognostic alignment to ELF 9.00.

Table S19. ELF, VCTE and combinations vs NASH CRN stage at 1-year timepoint (F4 vs <F4)

Analysis	ELF Cutoff	VCTE Cutoff (kPa)	TP	FP	FN	TN	Sensitivity (95% CI)	Specificity (95% CI)	Total Agreement (95% CI)
ELF Only	8.30	---	749	668	10	16	98.7% (97.6%, 99.4%)	2.3% (1.3%, 3.8%)	53.0% (50.4%, 55.6%)
	9.00	---	732	585	27	99	96.4% (94.9%, 97.6%)	14.5% (11.9%, 17.3%)	57.6% (55.0%, 60.2%)
	9.80	---	646	395	113	289	85.1% (82.4%, 87.6%)	42.3% (38.5%, 46.1%)	64.8% (62.3%, 67.3%)
	10.50	---	464	201	295	483	61.1% (57.6%, 64.6%)	70.6% (67.0%, 74.0%)	65.6% (63.1%, 68.1%)
	11.30	---	238	56	521	628	31.4% (28.1%, 34.8%)	91.8% (89.5%, 93.8%)	60.0% (57.4%, 62.6%)
	12.00	---	90	12	669	672	11.9% (9.6%, 14.4%)	98.2% (97.0%, 99.1%)	52.8% (50.2%, 55.4%)
VCTE Only	---	5.0	757	667	2	17	99.7% (99.1%, 100.0%)	2.5% (1.5%, 3.9%)	53.6% (51.0%, 56.2%)
	---	8.0[a]	746	548	13	136	98.3% (97.1%, 99.1%)	19.9% (17.0%, 23.1%)	61.1% (58.6%, 63.6%)
	---	10.0[a]	712	432	47	252	93.8% (91.9%, 95.4%)	36.8% (33.2%, 40.6%)	66.8% (64.3%, 69.2%)
	---	12.0	651	321	108	363	85.8% (83.1%, 88.2%)	53.1% (49.3%, 56.9%)	70.3% (67.8%, 72.6%)
	---	20.0	396	79	363	605	52.2% (48.6%, 55.8%)	88.5% (85.8%, 90.7%)	69.4% (66.9%, 71.7%)
	---	30.0	172	21	587	663	22.7% (19.7%, 25.8%)	96.9% (95.3%, 98.1%)	57.9% (55.3%, 60.4%)
	---	50.0	46	1	713	683	6.1% (4.5%, 8.0%)	99.9% (99.2%, 100.0%)	50.5% (47.9%, 53.1%)
ELF/VCTE Combo	8.30	5.0	747	654	12	30	98.4% (97.3%, 99.2%)	4.4% (3.0%, 6.2%)	53.8% (51.2%, 56.4%)
	9.00	8.0[a]	722	489	37	195	95.1% (93.3%, 96.5%)	28.5% (25.2%, 32.1%)	63.5% (61.0%, 66.0%)
	9.00	10.0[a]	693	393	66	291	91.3% (89.1%, 93.2%)	42.5% (38.8%, 46.3%)	68.2% (65.7%, 70.6%)
	9.80	12.0	575	232	184	452	75.8% (72.5%, 78.8%)	66.1% (62.4%, 69.6%)	71.2% (68.8%, 73.5%)
	10.50	20.0	301	48	458	636	39.7% (36.2%, 43.2%)	93.0% (90.8%, 94.8%)	64.9% (62.4%, 67.4%)
	11.30	30.0	99	6	660	678	13.0% (10.7%, 15.6%)	99.1% (98.1%, 99.7%)	53.8% (51.2%, 56.4%)
	12.00	50.0	20	0	739	684	2.6% (1.6%, 4.0%)	100.0% (99.5%, 100.0%)	48.8% (46.2%, 51.4%)

TP = True Positive, FP = False Positive, FN = False Negative, TN = True Negative

[a] VCTE cutoffs of 8.0 and 10.0 kPa both have prognostic alignment to ELF 9.00.

Table S20. ELF vs NASH CRN stage at 1-year timepoint for fibrosis staging (expanded dataset)

Comparison	ELF Cutoff	TP	FP	FN	TN	Sensitivity (95% CI)	Specificity (95% CI)	Total Agreement (95% CI)
**≥F2 vs <F2**	8.30	2306	40	40	4	98.3% (97.7%, 98.8%)	9.1% (2.5%, 21.7%)	96.7% (95.9%, 97.3%)
	9.00	2149	27	197	17	91.6% (90.4%, 92.7%)	38.6% (24.4%, 54.5%)	90.6% (89.4%, 91.8%)
	9.80	1703	11	643	33	72.6 (70.7%, 74.4%)	75.0% (59.7%, 86.8%)	72.6% (70.8%, 74.4%)
	10.50	1121	4	1225	40	47.8% (45.7%, 49.8%)	90.9% (78.3%, 97.5%)	48.6% (46.6%, 50.6%)
	11.30	510	1	1836	43	21.7% (20.1%, 23.5%)	97.7% (88.0%, 99.9%)	23.1% (21.5%, 24.9%)
	12.00	200	1	2146	43	8.5% (7.4%, 9.7%)	97.7% (88.0%, 99.9%)	10.2% (9.0%, 11.4%)
**≥F3 vs <F3**	8.30	2191	155	32	12	98.6% (98.0%, 99.0%)	7.2% (3.8%, 12.2%)	92.2% (91.0%, 93.2%)
	9.00	2061	115	162	52	92.7% (91.6%, 93.8%)	31.1% (24.2%, 38.8%)	88.4% (87.1%, 89.7%)
	9.80	1667	47	556	120	75.0% (73.1%, 76.8%)	71.9% (64.4%, 78.5%)	74.8% (73.0%, 76.5%)
	10.50	1105	20	1118	147	49.7% (47.6%, 51.8%)	88.0% (82.1%, 92.5%)	52.4% (50.4%, 54.4%)
	11.30	508	3	1715	164	22.9% (21.1%, 24.7%)	98.2% (94.8%, 99.6%)	28.1% (26.3%, 30.0%)
	12.00	199	2	2024	165	9.0% (7.8%, 10.2%)	98.8% (95.7%, 99.9%)	15.2% (13.8%, 16.7%)
**F4 vs <F4**	8.30	1263	1083	15	29	98.8% (98.1%, 99.3%)	2.6% (1.8%, 3.7%)	54.1% (52.0%, 56.1%)
	9.00	1231	945	47	167	96.3% (95.1%, 97.3%)	15.0% (13.0%, 17.3%)	58.5% (56.5%, 60.5%)
	9.80	1088	626	190	486	85.1% (83.1%, 87.0%)	43.7% (40.8%, 46.7%)	65.9% (63.9%, 67.8%)
	10.50	809	316	469	796	63.3% (60.6%, 66.0%)	71.6% (68.8%, 74.2%)	67.2% (65.2%, 69.0%)
	11.30	424	87	854	1025	33.2% (30.6%, 35.8%)	92.2% (90.4%, 93.7%)	60.6% (58.6%, 62.6%)
	12.00	180	21	1098	1091	14.1% (12.2%, 16.1%)	98.1% (97.1%, 98.8%)	53.2% (51.2%, 55.2%)

TP = True Positive, FP = False Positive, FN = False Negative, TN = True Negative

8. TRIPOD Statement Checklist

Section/Topic	Item	Development (D) or Validation (V)?	Checklist Item	Section in Manuscript
**Title and Abstract**				
Title	1	D;V	Identify the study as developing and/or validating a multivariable prediction model, the target population, and the outcome to be predicted.	Title
Abstract	2	D;V	Provide a summary of objectives, study design, setting, participants, sample size, predictors, outcome, statistical analysis, results, and conclusions.	Abstract
**Introduction**				
Background and objectives	3a	D;V	Explain the medical context (including whether diagnostic or prognostic) and rationale for developing or validating the multivariable prediction model, including references to existing models.	Introduction
	3b	D;V	Specify the objectives, including whether the study describes the development or validation of the model, or both.	Introduction
**Methods**				
Source of data	4a	D;V	Describe the study design or source of data (e.g., randomized trial, cohort, or registry data), separately for the development and validation data sets, if applicable.	Methods (Study population)
	4b	D;V	Specify the key study dates, including start of accrual; end of accrual; and, if applicable, end of follow-up.	Table S2
Participants	5a	D;V	Specify key elements of the study setting (e.g., primary care, secondary care, general population) including number and location of centres.	Table S2
	5b	D;V	Describe eligibility criteria for participants.	Table S2
	5c	D;V	Give details of treatments received, if relevant.	Table S2

Section/Topic	Item	Development (D) or Validation (V)?	Checklist Item	Section in Manuscript
Outcome	6a	D;V	Clearly define the outcome that is predicted by the prediction model, including how and when assessed.	Methods<br>(Analyses and endpoints)
	6b	D;V	Report any actions to blind assessment of the outcome to be predicted.	Methods<br>(Study assessments)
Predictors	7a	D;V	Clearly define all predictors used in developing the multivariable prediction model, including how and when they were measured.	Methods<br>(Analyses and endpoints)
	7b	D;V	Report any actions to blind assessment of predictors for the outcome and other predictors.	Methods<br>(Study assessments)
Sample size	8	D;V	Explain how the study size was arrived at.	Methods<br>(Study population)
Missing data	9	D;V	Describe how missing data were handled (e.g., complete-case analysis, single imputation, multiple imputation) with details of any imputation method.	Study inclusion required samples with results for ELF, VCTE and FIB-4 at baseline. There were no missing data for critical variables.
Statistical analysis methods	10a	D	Describe how predictors were handled in the analyses.	N/A
	10b	D	Specify type of model, all model-building procedures (including any predictor selection), and method for internal validation.	N/A
	10c	V	For validation, describe how the predictions were calculated.	Methods<br>(Analyses and endpoints)
	10d	D;V	Specify all measures used to assess model performance and, if relevant, to compare multiple models.	Methods<br>(Analyses and endpoints)
	10e	V	Describe any model updating (e.g., recalibration) arising from the validation, if done.	N/A

Section/Topic	Item	Development (D) or Validation (V)?	Checklist Item	Section in Manuscript
Risk groups	11	D;V	Provide details on how risk groups were created, if done.	Methods<br>(Analyses and endpoints)
Development vs. validation	12	V	For validation, identify any differences from the development data in setting, eligibility criteria, outcome, and predictors.	Methods<br>(Analyses and endpoints)
**Results**				
Participants	13a	D;V	Describe the flow of participants through the study, including the number of participants with and without the outcome and, if applicable, a summary of the follow-up time. A diagram may be helpful.	Fig. S1
	13b	D;V	Describe the characteristics of the participants (basic demographics, clinical features, available predictors), including the number of participants with missing data for predictors and outcome.	Table S2
	13c	V	For validation, show a comparison with the development data of the distribution of important variables (demographics, predictors and outcome).	Discussion
Model development	14a	D	Specify the number of participants and outcome events in each analysis.	N/A
	14b	D	If done, report the unadjusted association between each candidate predictor and outcome.	N/A
Model specification	15a	D	Present the full prediction model to allow predictions for individuals (i.e., all regression coefficients, and model intercept or baseline survival at a given time point).	N/A
	15b	D	Explain how to use the prediction model.	N/A
Model performance	16	D;V	Report performance measures (with CIs) for the prediction model.	Results

Section/Topic	Item	Development (D) or Validation (V)?	Checklist Item	Section in Manuscript
Model updating	17	V	If done, report the results from any model updating (i.e., model specification, model performance).	N/A
**Discussion**				
Limitations	18	D;V	Discuss any limitations of the study (such as nonrepresentative sample, few events per predictor, missing data).	Discussion
Interpretation	19a	V	For validation, discuss the results with reference to performance in the development data, and any other validation data.	Discussion
	19b	D;V	Give an overall interpretation of the results, considering objectives, limitations, results from similar studies, and other relevant evidence.	Discussion
Implications	20	D;V	Discuss the potential clinical use of the model and implications for future research.	Discussion
**Other information**				
Supplementary information	21	D;V	Provide information about the availability of supplementary resources, such as study protocol, Web calculator, and data sets.	References to Supplemental Tables and Figures listed throughout text
Funding	22	D;V	Give the source of funding and the role of the funders for the present study.	N/A

9. References

1. Loomba R, Huang DQ, Sanyal AJ, et al. Liver stiffness thresholds to predict disease progression and clinical outcomes in bridging fibrosis and cirrhosis. *Gut*. Mar 2023;72(3):581-589. doi:10.1136/gutjnl-2022-327777
2. de Franchis R, Bosch J, Garcia-Tsao G, Reiberger T, Ripoll C, Baveno VIIF. Baveno VII - Renewing consensus in portal hypertension. *J Hepatol*. Apr 2022;76(4):959-974. doi:10.1016/j.jhep.2021.12.022
3. Harrison SA, Abdelmalek MF, Caldwell S, et al. Simtuzumab Is Ineffective for Patients With Bridging Fibrosis or Compensated Cirrhosis Caused by Nonalcoholic Steatohepatitis. *Gastroenterology*. Oct 2018;155(4):1140-1153. doi:10.1053/j.gastro.2018.07.006
4. Sanyal AJ, Harrison SA, Ratziu V, et al. The Natural History of Advanced Fibrosis Due to Nonalcoholic Steatohepatitis: Data From the Simtuzumab Trials. *Hepatology*. Dec 2019;70(6):1913-1927. doi:10.1002/hep.30664
5. Harrison SA, Wong VW, Okanoue T, et al. Selonsertib for patients with bridging fibrosis or compensated cirrhosis due to NASH: Results from randomized phase III STELLAR trials. *J Hepatol*. Jul 2020;73(1):26-39. doi:10.1016/j.jhep.2020.02.027
6. Younossi ZM, Anstee QM, Wai-Sun Wong V, et al. The Association of Histologic and Noninvasive Tests With Adverse Clinical and Patient-Reported Outcomes in Patients With Advanced Fibrosis Due to Nonalcoholic Steatohepatitis. *Gastroenterology*. Apr 2021;160(5):1608-1619 e13. doi:10.1053/j.gastro.2020.12.003
7. Sanyal AJ, Anstee QM, Trauner M, et al. Cirrhosis regression is associated with improved clinical outcomes in patients with nonalcoholic steatohepatitis. *Hepatology*. May 2022;75(5):1235-1246. doi:10.1002/hep.32204
8. Chalasani N, Abdelmalek MF, Garcia-Tsao G, et al. Effects of Belapectin, an Inhibitor of Galectin-3, in Patients With Nonalcoholic Steatohepatitis With Cirrhosis and Portal Hypertension. *Gastroenterology*. Apr 2020;158(5):1334-1345 e5. doi:10.1053/j.gastro.2019.11.296
9. Are VS, Vuppalanchi R, Vilar-Gomez E, Chalasani N. Enhanced Liver Fibrosis Score Can Be Used to Predict Liver-Related Events in Patients With Nonalcoholic Steatohepatitis and Compensated Cirrhosis. *Clin Gastroenterol Hepatol*. Jun 2021;19(6):1292-1293 e3. doi:10.1016/j.cgh.2020.06.070
10. Loomba R, Noureddin M, Kowdley KV, et al. Combination Therapies Including Cilofexor and Firsocostat for Bridging Fibrosis and Cirrhosis Attributable to NASH. *Hepatology*. Feb 2021;73(2):625-643. doi:10.1002/hep.31622
