# Supplementary material for: Aligned ELF and VCTE cut-offs facilitate risk staging in patients with metabolic dysfunction-associated steatohepatitis
Source: JHEP Rep. 2026 Sep 18;8(12):102041. doi: 10.1016/j.jhepr.2026.102041 (PMC13770651; doi:10.1016/j.jhepr.2026.102041)
Supplement: Supplementary file 2 [file mmc2.pdf]

JHEP|Reports

JHEP Reports

CTAT methods

Tables for a "Complete, Transparent, Accurate and Timely account" (CTAT) are now mandatory for all revised submissions. The aim is to enhance the reproducibility of methods.

- Only include the parts relevant to your study
- Refer to the CTAT in the main text as 'Supplementary CTAT Table'
- Do not add subheadings
- Add as many rows as needed to include all information
- Only include one item per row

If the CTAT form is not relevant to your study, please outline the reasons why:

Only sections 1.8 and 2.0 are applicable. See below.

1.1 Antibodies

Name	Citation	Supplier	Cat no.	Clone no.

1.2 Cell lines

Name	Citation	Supplier	Cat no.	Passage no.	Authentication test method

1.3 Organisms

Name	Citation	Supplier	Strain	Sex	Age	Overall n number

1.4 Sequence based reagents

Name	Sequence	Supplier

1.5 Biological samples

Description	Source	Identifier

1.6 Deposited data

Name of repository	Identifier	Link

Created : November, 2018

1.7 Software

Software name	Manufacturer	Version

1.8 Other (*e.g.* drugs, proteins, vectors etc.)

The ELF Test was performed using ADVIA Centaur reagents with testing completed on ADVIA Centaur XP systems. Siemens Healthcare Diagnostics Inc. (Tarrytown, NY) is the manufacturer of the ELF reagents and ADVIA Centaur XP system.

1.9 Please provide the details of the corresponding methods author for the manuscript:

N/A

2.0 Please confirm for randomised controlled trials all versions of the clinical protocol are included in the submission. These will be published online as supplementary information.

Clinical trial protocols are provided with the original reports for these studies as cited in the manuscript.
