# Supplementary material for: Aligned ELF and VCTE cut-offs facilitate risk staging in patients with metabolic dysfunction-associated steatohepatitis
Source: JHEP Rep. 2026 Sep 18;8(12):102041. doi: 10.1016/j.jhepr.2026.102041 (PMC13770651; doi:10.1016/j.jhepr.2026.102041)
Supplement: Supplementary file 4 [file mmc4.pdf]

Aligned ELF and VCTE cut-offs facilitate risk staging in patients with metabolic dysfunction-associated steatohepatitis

Authors

Matthew F.W. Gee, Andrew N. Billin, Donald B. Chalfin, …, Jordan J. Feld, Keyur Patel, **Arun J. Sanyal,**

Correspondence

matt.gee@mail.utoronto.ca (M.F.W. Gee).

Graphical abstract

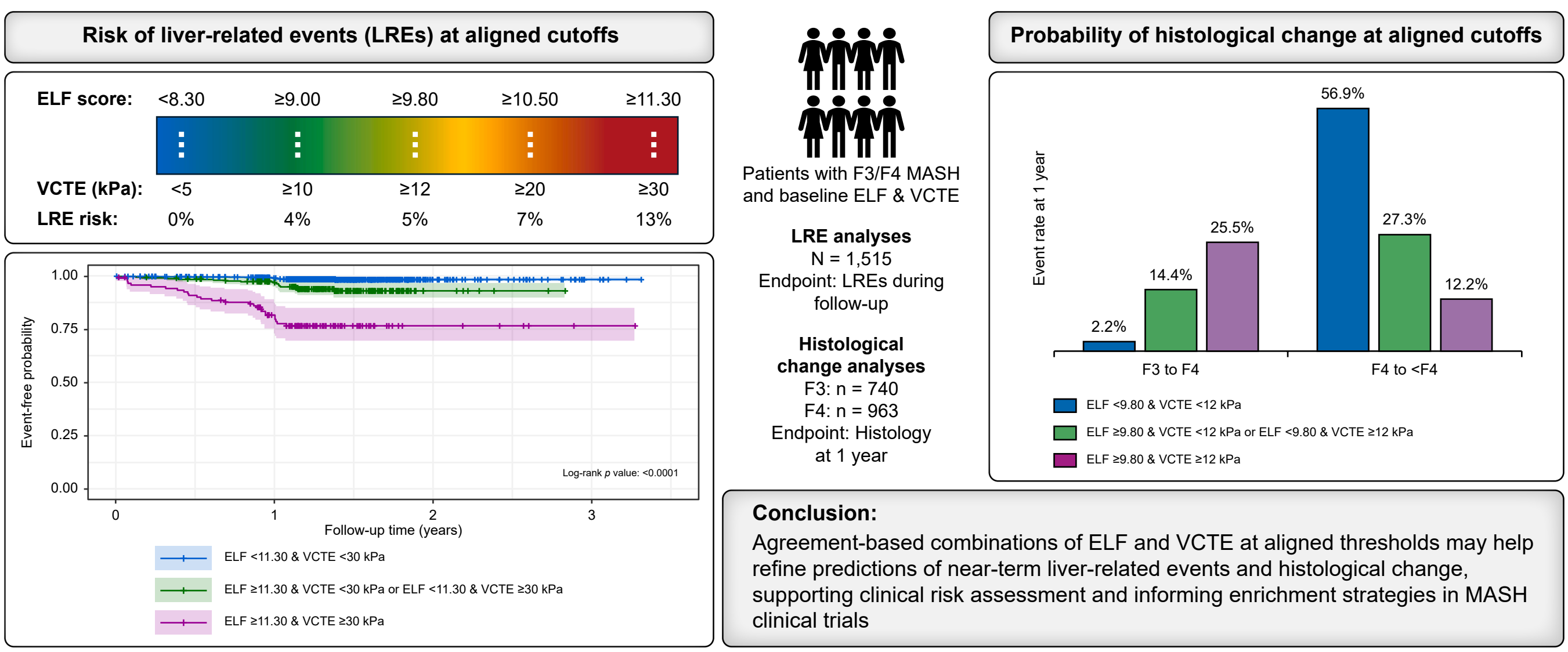

Highlights:

- ELF and VCTE thresholds can be aligned to enable comparable risk staging.
- Stratification based on ELF/VCTE agreement predicts clinical outcomes and fibrosis trajectory.
- Concordantly high ELF/VCTE results identify patients most likely to progress.
- Concordantly low ELF/VCTE results identify patients most likely to regress.
- Discordant ELF/VCTE results identify an intermediate-risk patient population.

Impact and implications:

Current MASH care pathways increasingly rely on non-invasive tests, yet recommended enhanced liver fibrosis (ELF) and vibration-controlled transient elastography (VCTE) thresholds have largely been established independently. We found that ELF and VCTE thresholds can be aligned according to comparable risk and that concordant and discordant test results provide clinically meaningful stratification of both liver-related outcomes and likelihood of histological progression and regression. These findings may help support more consistent risk assessment and patient stratification across clinical practice and research settings.

https://doi.org/10.1016/j.jhepr.2026.102041
© 2026 The Authors. Published by Elsevier B.V. on behalf of European Association for the Study of the Liver (EASL). This is an open access article under the CC BY license (http://creativecommons.org/licenses/by/4.0/).

Aligned ELF and VCTE cut-offs facilitate risk staging in patients with metabolic dysfunction-associated steatohepatitis

Matthew F.W. Gee[1,2,*], Andrew N. Billin[3], Donald B. Chalfin[2,4], Rachel Fonstad[2], Edward B. De Vol[2], Jordan J. Feld[1,5], Keyur Patel[1,5,‡], Arun J. Sanyal[6,‡]

JHEP Reports **2026**. vol. 8 | 1–11

Check for updates

Background & Aims: Clinical practice guidance for non-invasive test (NIT)-based management of metabolic dysfunction-associated steatohepatitis (MASH) includes the enhanced liver fibrosis (ELF) test and vibration-controlled transient elastography (VCTE). However, recommended thresholds were derived independently and not calibrated to yield equivalent liver-related event (LRE) risk across tests. We aimed to identify ELF and VCTE cut-offs that are prognostically aligned and to evaluate whether agreement-based combinations at aligned thresholds improve near-term risk stratification.

Methods: Individual-level data were pooled from patients across placebo-controlled trials of MASH and advanced fibrosis. Baseline ELF and VCTE were evaluated in relation to subsequent LREs and histological change at 1 year. Prognostic alignment was performed by identifying VCTE thresholds associated with observed LRE risks corresponding to pre-specified ELF cut-offs. The prognostic performance of individual tests and ELF/VCTE combinations was assessed using event risk, likelihood ratios, Kaplan-Meier analysis, and discrimination metrics (C-index and AUROC).

Results: Risk-aligned pairings supported ELF 9.80/VCTE 12.0 kPa, ELF 10.50/VCTE 20.0 kPa and ELF 11.30/VCTE 30.0 kPa. Agreement-based ELF/VCTE stratification improved discrimination *vs*. VCTE alone (AUROC 0.779 *vs*. 0.705; $p = 0.0129$). At ELF 11.30/VCTE 30.0 kPa, LRE risk increased across negative concordance, discordance, and positive concordance strata (1.3%, 5.4%, and 21.6%, respectively). For prediction of histological change using ELF 9.80/VCTE 12.0 kPa, F3-to-F4 progression rates were 2.2%, 14.4%, and 25.5%, and F4 regression rates were 56.9%, 27.3%, and 12.2% across negative concordance, discordance, and positive concordance strata, respectively.

Conclusions: ELF and VCTE thresholds can be aligned for comparable prognostic risk staging in advanced MASH. Agreement-based combinations of ELF and VCTE at aligned thresholds may help refine prediction of near-term LREs and histological change, supporting clinical risk assessment and informing MASH clinical trial enrichment strategies.

© 2026 The Authors. Published by Elsevier B.V. on behalf of European Association for the Study of the Liver (EASL). This is an open access article under the CC BY license (http://creativecommons.org/licenses/by/4.0/).

Introduction

Metabolic dysfunction-associated steatotic liver disease (MASLD) is the most common chronic liver disease worldwide, with a global prevalence of up to 38% and a prevalence as high as 65% among individuals with type 2 diabetes mellitus.[1–6] Metabolic dysfunction-associated steatohepatitis (MASH), characterized by hepatic steatosis, inflammation, and hepatocyte ballooning, is the more active form of the disease and a leading cause of cirrhosis complications, hepatocellular carcinoma, indication for transplantation, and liver-related death.[3,7,8]

Due to the invasive nature of liver biopsy and the inherent variability of histological findings, non-invasive tests (NITs), such as the enhanced liver fibrosis (ELF) test and liver stiffness measurement (LSM) by vibration-controlled transient elastography (VCTE), are recommended by clinical practice guidance from the American Association for the Study of Liver Diseases (AASLD) and the European Association for the Study of the Liver (EASL) as useful methods for detecting advanced fibrosis and assessing the risk of disease progression following first-line screening with Fibrosis-4 (FIB-4).[3,9,10]

The scientific literature delineates many different NIT cut-offs, with studies often deriving cut-offs *de novo* and optimizing them for their study population (*e.g*. quartiles, Youden index). This has led to non-standardized thresholds, such as ELF ≥9.50, often derived independently for either diagnostic or prognostic use.[11] Although there is more consistency amongst the cut-off recommendations in clinical practice guidelines,[3,9] the thresholds for different NITs were derived independently from one another.[12,13] The lack of head-to-head NIT cut-off validation in the same population is problematic due to the heterogeneity across study populations and test thresholds,

* Corresponding author. Address: Institute of Medical Science, Temerty Faculty of Medicine, University of Toronto, Toronto, Ontario, Canada.
E-mail address: matt.gee@mail.utoronto.ca (M.F.W. Gee).
‡ These authors contributed equally to the study.
https://doi.org/10.1016/j.jhepr.2026.102041

along with limited data on comparative studies, as was highlighted as a limitation in a recent AASLD systematic review of blood-based NITs for staging fibrosis in chronic liver disease.[14–16] Consequently, although many of the thresholds for ELF, VCTE, and FIB-4 recommended in clinical practice guidelines are supported by studies demonstrating good performance of individual NITs, whether thresholds across different NITs can yield equivalent diagnostic and prognostic performance for a given purpose has not been fully explored.

Studies have shown that ELF and VCTE prognosticate liver-related clinical outcomes, as would be expected due to their correlation with histology, a well-established predictor of clinical outcomes.[17–26] However, the guideline-recommended thresholds for these NITs were primarily established based on their correlation with histological fibrosis stage rather than their prognostic performance.[12,13] Rather than simply using NITs as a surrogate for histology, it is essential to establish test performance in terms of clinical outcome risk.[27] Accordingly, a common dataset is necessary to permit test comparisons and to determine which thresholds across NIT modalities are equivalent with respect to prognostic performance. However, to the best of our knowledge, no studies have evaluated which ELF and VCTE thresholds are most aligned in relation to the risk of clinical outcomes.

To compare the ability of NITs to identify histological fibrosis stage and predict clinical events, we analyzed data from large placebo-controlled trials in patients with advanced fibrosis due to MASH. This pooled dataset includes NIT measurements, biopsy results and recorded liver-related clinical outcomes, thereby permitting direct performance comparisons between the NITs. In this study, we aimed to determine which ELF and VCTE cut-offs yield equivalent prognostic performance for liver-related clinical outcomes, and whether combining ELF and VCTE at aligned thresholds improves risk assessment compared with either test alone.

Patients and methods

Study population

This study pooled individual-level data from previously published, randomized, placebo-controlled phase II and III trials in participants with advanced fibrosis (F3 & F4) due to MASH, which evaluated selonsertib (STELLAR-3 [NCT03053050], STELLAR-4 [NCT03053063]), simtuzumab (GS-US-321-0105 [NCT01672866], GS-US-321-0106 [NCT01672879]), and belapectin (NASH-CX [NCT02462967]). An additional study was included for assessment of histological change at 1 year (ATLAS [NCT03449446]).

The NASH-CX study concluded after the planned follow-up period of 52 weeks, while the selonsertib and simtuzumab studies were discontinued after their interim analyses at 48 weeks and 96 weeks, respectively, after determining that these therapies were ineffective. Preliminary findings from these studies, including analyses of the ELF test, and detailed methods have been reported previously.[17–19,21,22,28–31] As there were no meaningful differences between placebo and active treatment groups, all study arms were pooled for the analyses conducted in this study. Additionally, trial populations were pooled where appropriate to address study endpoints. A summary of relevant characteristics of the five clinical trials and the populations used for each analysis is presented in Table S1. The studies were approved by the institutional review boards or independent ethics committees at all participating sites and conducted in accordance with the Declarations of Helsinki and Istanbul. All participants provided informed consent.

Study assessments

Participants included in the analysis had complete baseline measurements for VCTE and laboratory tests (including ELF) and had recorded follow-up for clinical outcomes (Fig. S1).

Liver fibrosis was staged at the time of enrollment (all studies), Week 48 (STELLAR-3, STELLAR-4, GS-US-321-0105, GS-US-321-0106, ATLAS), and Week 52 (NASH-CX). All biopsies were read by a single central reader (Dr. Zachary Goodman, Inova Health System) who was blinded to treatment assignment but not biopsy sequence. Histological assessments included, among other features, the adequacy of the biopsy specimen, confirmation of the diagnosis, fibrosis stage according to the NASH Clinical Research Network (NASH CRN) criteria, and modified Ishak fibrosis classifications.[17–19,21,22,28–31]

The ELF test was performed using ADVIA Centaur XP systems (Siemens Healthcare Diagnostics Inc., Tarrytown, NY, USA), with the ELF score automatically calculated by the instrument. The ELF test is a non-invasive serum-based test that quantifies three extracellular matrix components reflecting liver fibrosis and fibrogenesis: hyaluronic acid, amino-terminal propeptide of type III procollagen, and tissue inhibitor of matrix metalloproteinase 1.[1–3] Results are combined in an algorithm, calculated automatically by the system, that yields a unitless ELF score.[4–6]

LSM by VCTE was performed according to manufacturer recommendations using FibroScan (Echosens, Paris, France) by trained operators, with participants in a fasting state and following standard reliability and probe selection criteria, as previously described.[22,32] Fasting blood samples were used to calculate the FIB-4 score according to the published formula.[33]

Liver-related events (LREs) were adjudicated by a central committee of experts and defined as clinically apparent ascites requiring treatment, Grade ≥2 hepatic encephalopathy according to the West Haven criteria that required treatment, and portal hypertension-related gastrointestinal bleeding, liver transplantation, qualification for transplantation (model for end-stage liver disease ≥15), liver-related death, or hepatocellular carcinoma.[17–19,21,22,28–31]

Analyses and endpoints

The primary aims of the study were to 1) determine which established ELF and VCTE cut-offs yield equivalent prognostic performance for LREs and 2) evaluate whether agreement-based combinations of ELF and VCTE at aligned thresholds could further stratify near-term LRE risk and improve prognostic performance compared with either test alone. The utility of agreement-based combinations of ELF and VCTE at aligned thresholds to predict histological change at 1 year was evaluated as a contextual, secondary analysis.

Statistical approaches

Observed risk of LREs was estimated as the proportion of participants with baseline NIT values at or above a given cut-off who experienced an event during follow-up. Exact binomial 95% CIs were calculated using the Clopper-Pearson method. Baseline ELF and VCTE measurements were categorized using thresholds described in clinical practice guidelines and prior studies.[3,9,22,34–36] Eight ELF cut-offs ranging from 7.70 to 12.80 and seven VCTE cut-offs ranging from 5.0 to 30.0 kPa were evaluated.

To align ELF and VCTE thresholds with respect to prognostic performance, event risks were calculated across the observed distributions of baseline ELF and VCTE values. For each ELF cut-off, the VCTE threshold yielding the closest observed LRE risk was identified by minimizing the absolute difference in event proportions above each threshold. This approach yielded risk-based alignments between ELF and VCTE cut-offs without assuming a linear or monotonic relationship between test values. To facilitate clinical interpretation, these empirically derived thresholds were mapped to the nearest established VCTE cut-offs used in prior studies and guidelines, and corresponding event risks were recalculated with exact binomial confidence intervals, as described in the Supplemental Materials. Inverse mapping was also performed to identify ELF cut-offs corresponding to established VCTE thresholds to verify the robustness of the risk-based alignment strategy.

Risk stratification according to ELF and VCTE, alone and in combination, and according to histologic fibrosis stage was further characterized using multiple complementary metrics, including absolute event rate, interval likelihood ratio, and incidence of LREs per 1,000 person-years of follow-up. Absolute event rate was calculated as the number of events divided by the number of participants within each stratum, with exact 95% CIs. Interval likelihood ratios were calculated as the ratio of the proportion of events occurring within a given stratum to the proportion of non-events occurring within that stratum. Time-to-event analyses were performed using Cox proportional hazards regression, with follow-up time expressed in years and LREs as the outcome. Kaplan-Meier methods were used to estimate event-free probability across the various risk strata.

For model-based discrimination analyses, Cox proportional hazards models were specified using categorical risk strata derived from single-test ELF and VCTE thresholds and from agreement-based combinations of aligned ELF and VCTE cut-offs. Single-test models were constructed using guideline-recommended thresholds for advanced fibrosis (ELF 9.80; VCTE 12.0 kPa) and cirrhosis rule-in (ELF 11.30; VCTE 20.0 kPa), which defined low-, intermediate-, and high-risk strata.[3,9] These models were compared with an agreement-based combination model defined using aligned thresholds of ELF 11.30 and VCTE 30.0 kPa to evaluate relative discrimination for LREs. Model discrimination was assessed using Harrell's concordance index (C-index). Model fit and relative performance were compared using log-likelihood, Akaike information criterion (AIC), and Bayesian information criterion (BIC), with lower AIC/BIC and higher log-likelihood indicating improved model fit.

Receiver-operating characteristic (ROC) analyses were used to further assess discrimination of agreement-based combination models relative to single-test models. These included logistic regression-based step-function ROC with comparison of area under the ROC curve (AUROC) using DeLong's test, Cox regression-based ROC, and time-dependent ROC analyses.

All statistical analyses were performed using R version 4.4.2 (R Foundation for Statistical Computing, Vienna, Austria), including the *survival*, *survivalROC*, *timeROC*, *ggsurvfit*, and *ggplot2* packages. Two-sided p values <0.05 were considered statistically significant.

Results

Demographics, baseline characteristics and clinical outcomes

Full demographic details for the studies included in this analysis have previously been described.[17–19,21,22,28–31] Summaries of the baseline demographics and clinical characteristics are presented in Table 1. Baseline mean and median VCTE measurements arranged by baseline ELF strata are presented in Table S2. The median (Q1, Q3) follow-up time in this dataset was 15.7 (13.5, 18.3) months. A total of 57 LREs were recorded during the follow-up period (Table S3).

Prognostic alignment

In this dataset, the probability of LREs increased non-linearly with baseline ELF, remaining low across much of the lower range before rising sharply at ELF values greater than 11.30, indicating a pronounced inflection at the upper end of the distribution (Fig. 1A). In contrast, baseline VCTE showed a more gradual increase in event probability across its range, without a clear inflection point (Fig. 1B). Given the non-linear relationship of ELF and VCTE with LRE risk, prognostic alignment was performed by identifying VCTE thresholds associated with event risks comparable to predefined ELF cut-offs.

Patients with baseline ELF scores greater than or equal to 7.70, 8.30, 9.00, 9.80, 10.50, 11.30, 12.00, and 12.80 had LRE risks of 3.8%, 3.8%, 4.1%, 4.9%, 7.2%, 12.8%, 21.3%, and 47.4%, respectively. VCTE cut-offs with event risks comparable to those for the predetermined ELF thresholds were 2.5, 5.0, 9.6, 12.4, 18.6, 29.2, 51.8, and 75.0 kPa, respectively. Although baseline ELF ≥12.80 was associated with a 47.4% risk of LREs, the highest point estimate for risk obtainable by VCTE (at the upper analytical range of 75 kPa) was 33.3% (Table 2). Inverse mapping to identify ELF cut-offs corresponding to established VCTE thresholds yielded similar findings (Table S4), and similar trends were observed when aligning ELF and FIB-4 cut-offs (Table S5). Additionally, the LRE risk associated with NASH CRN stage F4 in this population was 5.9% (95% CI 4.4–7.7%), with ELF and VCTE cut-offs of ≥10.14 and ≥15.5 kPa, respectively, corresponding to an equivalent risk. Conversely, the LRE risk associated with F3 fibrosis in this population was 1.1% (95% CI 0.4–2.2%), corresponding to ELF and VCTE cut-offs of <10.39 and <14.0 kPa, respectively (Table S6). Similar findings were observed when analyses were restricted to events occurring within 18 months of baseline assessment (Table S7).

Table 1. Baseline demographics and clinical characteristics.

Baseline parameter	Main dataset (progression to LREs)	Supplementary dataset (histological changes)
N	1,515	1,703
Female (%)	915 (60%)	1,041 (61%)
Type 2 diabetes present (%)	1,097 (72%)	1,237 (73%)
Age category (%)		
≤34	22 (1%)	20 (1%)
35-39	36 (2%)	37 (1%)
40-44	71 (5%)	73 (4%)
45-49	148 (10%)	154 (9%)
50-54	204 (13%)	222 (13%)
55-59	332 (22%)	381 (22%)
60-64	328 (22%)	377 (22%)
≥65	374 (25%)	439 (26%)
NASH CRN stage (%)		
F3	660 (44%)	740 (43.5%)
F4	853 (56%)	963 (56.5%)
Missing	2 (0.1%)	N/A
Median ELF score (IQR)	10.31 (9.69, 11.02)	10.26 (9.64, 10.96)
Median FIB-4 score (IQR)	2.16 (1.44, 3.24)	2.13 (1.44, 3.08)
Median VCTE in kPa (IQR)	16.8 (11.6, 25.2)	16.6 (11.5, 24.0)

ELF, enhanced liver fibrosis; FIB-4, fibrosis-4; LRE, liver-related event; NASH, non-alcoholic steatohepatitis; VCTE, vibration-controlled transient elastography.

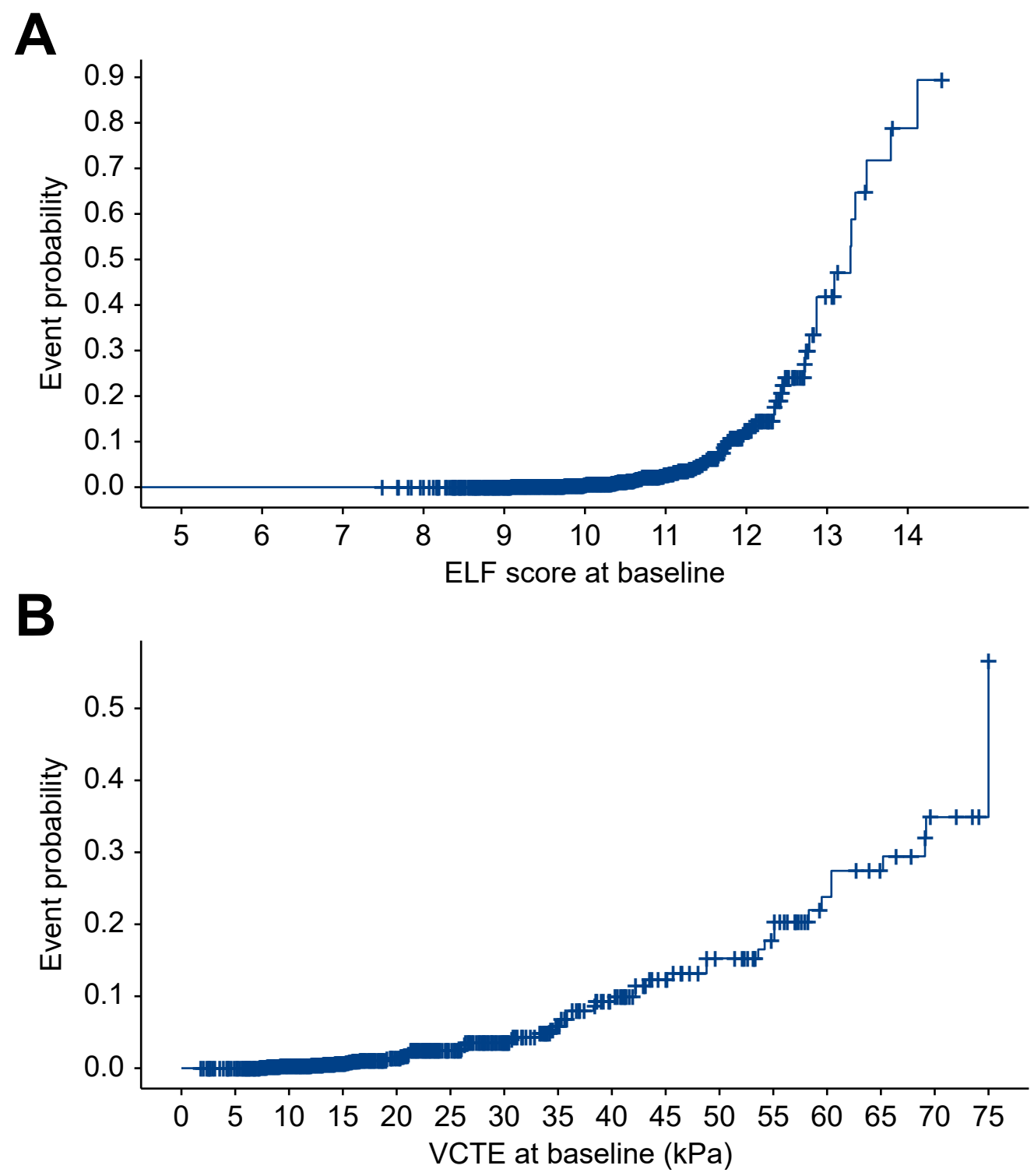

Fig. 1. Cumulative probability of liver-related clinical outcomes according to baseline NIT value, estimated using the Kaplan-Meier method. (A) ELF, (B) VCTE (kPa). ELF, enhanced liver fibrosis; NITs, non-invasive tests; VCTE, vibration-controlled transient elastography.

Many of the derived cut-offs were close to pre-established VCTE cut-offs of 5.0, 10.0, 12.0, 20.0, and 30.0 kPa. The risk of LREs at each of these pre-established cut-offs was not significantly different from the risk for the experimentally derived thresholds (Table 2). Similar trends between ELF and VCTE at the various cut-offs were observed using 12-month event risk to account for differences in follow-up time between patients (Table S8). Given that the same ELF and VCTE cut-offs are commonly used to assess liver fibrosis stage, diagnostic performance at these aligned thresholds was also evaluated against histology. At aligned cut-offs, ELF and VCTE demonstrated broadly comparable sensitivity and specificity with respect to histologic fibrosis stage, consistent with the intended use of these thresholds (Table S16–S20; Fig. S6).

NIT combinations

Having established ELF and VCTE clinical cut-offs aligned to predict LREs, we next evaluated the utility of combining these tests at their aligned thresholds to enhance near-term risk stratification (Table 3). For the ELF 9.80/VCTE 12.0 kPa combination, event risk remained low until both NITs were above threshold. For the ELF 10.50/VCTE 20.0 kPa and ELF 11.30/VCTE 30.0 kPa combinations, event risk was lowest when both NITs were below threshold (negative concordance), increased with discordance, and was highest when both tests were concordant above threshold (positive concordance). For example, within the ELF 11.30/VCTE 30.0 kPa pairing, event risk rose stepwise from 1.3% (95% CI 0.8%–2.2%) with negative concordance, to 5.4% (95% CI 3.1%–8.8%) with discordance, and to 21.6% (95% CI 14.7%–29.8%) with positive concordance. This stepwise increase in LRE risk across ELF and VCTE strata is illustrated in two-dimensional risk heatmaps (Fig. S2), which highlight the concentration of highest risk in patients with concordantly elevated ELF and VCTE values.

Across these cut-off pairings, event risk in the concordantly positive strata approximately doubled with increasing thresholds, from 5.9% (95% CI 4.4%–7.6%) for ELF 9.80/VCTE 12.0 kPa, to 10.0% (95% CI 7.2%–13.5%) for ELF 10.50/VCTE 20.0 kPa, and 21.6% (95% CI 14.7%–29.8%) for ELF 11.30/VCTE 30.0 kPa. Event risk did not differ significantly by Fisher's exact test between discordant strata, regardless of which test exceeded threshold. The ELF 9.80/VCTE 12.0 kPa pairing showed event risk comparable to NASH CRN F4 staging (5.9%), whereas higher threshold pairings were associated with markedly higher risk (Table S6).

To further characterize the discordant strata, baseline laboratory characteristics were compared across concordance groups (Table S8). Although patients with discordant results demonstrated similar LRE risks regardless of whether ELF or VCTE exceeded the threshold, biochemical profiles were not identical. Patients with ELF ≥11.30 and VCTE <30.0 kPa had higher AST, ALT, and ALP concentrations than patients with ELF <11.30 and VCTE ≥30.0 kPa, whereas platelet counts, GGT, and bilirubin levels were comparable between discordant groups. These findings suggest that ELF and VCTE may identify partially overlapping but distinct manifestations of advanced liver disease despite being associated with similar intermediate clinical risk.

Analyses using alternative, non-aligned cut-offs showed comparable discrimination, with modest variation in absolute risk. The positive concordant stratum for the ELF 11.30/VCTE 25.0 kPa pairing demonstrated slightly lower event risk (18.5%; 95% CI 12.7%–25.4%) than the corresponding aligned ELF

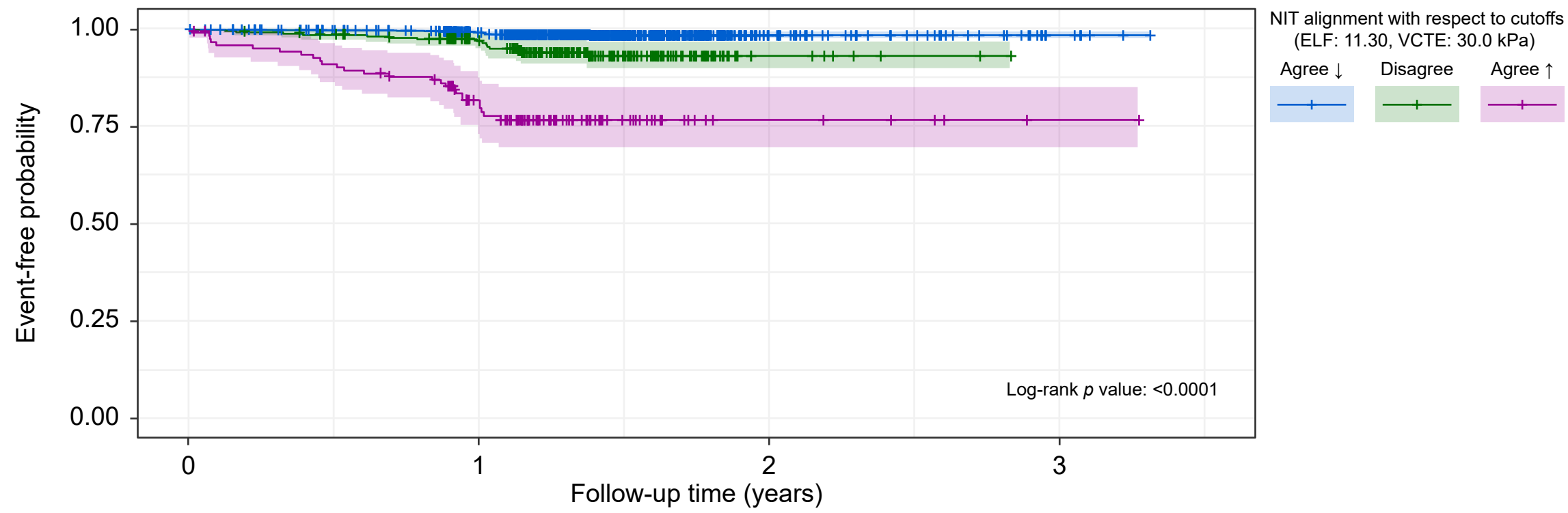

N° at risk				
Agree ↓	1,113 (0)	947 (9)	49 (15)	6 (15)
Disagree	277 (0)	199 (8)	7 (15)	0 (15)
Agree ↑	125 (0)	81 (22)	6 (27)	1 (27)

Fig. 2. Kaplan-Meier curves for event-free probability of LREs Risk stratification based on NIT alignment with respect to cut-offs (ELF:11.30 and VCTE:30.0 kPa). "Agree ↓" (Blue): ELF <11.30 and VCTE <30.0 kPa; "Disagree" (Green): ELF ≥11.30 and VCTE <30.0 kPa OR ELF <11.30 and VCTE ≥30.0 kPa; "Agree ↑" (Purple): ELF ≥11.30 and VCTE ≥30.0 kPa. ELF, enhanced liver fibrosis; LREs, liver-related events; NITs, non-invasive tests; VCTE, vibration-controlled transient elastography.

11.30/VCTE 30.0 kPa pairing (Tables S6 and S9). In addition, a three-NIT concordance approach incorporating ELF, VCTE, and FIB-4 yielded qualitatively similar risk stratification, with the highest LRE risk observed in the positive concordant stratum (26.2%; 95% CI 17.2%–36.9%) (Table S10).

As the ELF 11.30/VCTE 30.0 kPa pairing yielded the highest LRE risk across all two-NIT analyses evaluated, it was selected for further evaluation. Kaplan-Meier analysis showed clear and graded separation in event-free probability across concordant and discordant strata within the ELF 11.30/VCTE 30.0 kPa group (Fig. 2). By comparison, stratification by NASH CRN stage yielded minimal separation between F3 and F4 (Fig. S3), while the three-NIT concordance model showed comparable separation to the ELF/VCTE combination (Fig. S4).

To evaluate whether this framework also reflects underlying histological disease activity, we assessed its association with fibrosis progression and regression using thresholds relevant to fibrosis staging. Using lower aligned cut-offs commonly used for identification of advanced fibrosis (ELF 9.80 and VCTE 12.0 kPa),[3,9] we next evaluated whether agreement-based combinations of ELF and VCTE stratified the likelihood of short-term histological progression and regression (Table 4). Among patients with baseline F3 fibrosis, the probability of progression to F4 increased across concordance strata, rising from 2.2% (95% CI 0.6%–5.7%) in the negative agreement group to 25.4% (95% CI 20.3%–31.0%) in the positive agreement group, with intermediate risk observed in discordant strata (14.4%; 95% CI 10.6%–18.9%). Conversely, among patients with baseline F4 fibrosis, the likelihood of regression to <F4 was highest in the negative agreement group (56.9%; 95% CI 43.2%–69.8%) and lowest in the positive agreement group (12.2%; 95% CI 9.9%–14.9%), with discordant strata demonstrating intermediate probabilities (27.3%; 95% CI 21.6%–33.6%). Similar to the analyses of liver-related clinical outcomes, rates of histological change did not differ significantly between discordant strata, regardless of whether ELF or VCTE exceeded the threshold. These findings demonstrate a consistent, graded relationship between ELF/VCTE concordance and histological disease dynamics, whereby concordantly elevated values identify patients at higher risk of progression and lower likelihood of regression, while concordantly low values indicate a more favorable histological trajectory. We next evaluated whether this agreement-based risk

Table 2. Corresponding VCTE thresholds to established ELF cut-offs with respect to risk of LREs.

ELF		VCTE				
Established ELF cut-off	LRE risk (95% CI)	Empirically derived cut-off (kPa)	Cut-off range (kPa)[a]	LRE risk (95% CI)	Nearest VCTE cut-off in clinical use (kPa)	LRE risk (95% CI)
≥7.70	3.8% (2.9%–4.9%)	≥2.5	1.8 to 6.4	3.8% (2.9%–4.9%)	≥5.0	3.8% (2.9%–4.9%)
≥8.30	3.8% (2.9%–4.9%)	≥5.0	1.8 to 7.4	3.8% (2.9%–4.9%)		
≥9.00	4.1% (3.1%–5.3%)	≥9.6	8.6 to 10.0	4.1% (3.1%–5.3%)	≥10.0[c]	4.2% (3.1%–5.4%)
≥9.80	4.9% (3.7%–6.4%)	≥12.4	12.0 to 12.5	4.9% (3.7%–6.4%)	≥12.0	4.8% (3.6%–6.2%)
≥10.50	7.2% (5.3%–9.4%)	≥18.6	18.4 to 19.6	7.2% (5.3%–9.5%)	≥20.0	7.3% (5.4%–9.7%)
≥11.30	12.8% (9.1%–17.3%)	≥29.2	28.9 to 29.3	12.8% (9.1%–17.5%)	≥30.0[d]	13.4% (9.5%–18.3%)
≥12.00	21.3% (13.5%–30.9%)	≥51.8	51.5 to 54.8	21.3% (13.5%–30.9%)	N/A	N/A
≥12.80	47.4% (24.4%–71.1%)	≥75.0[a]	Not calculable	33.3% (11.8%–61.6%)[b]	N/A	N/A

ELF, enhanced liver fibrosis; LRE, liver-related event; VCTE, vibration-controlled transient elastography.
[a]Range of VCTE thresholds associated with an observed LRE risk within ±0.1 percentage points of the corresponding ELF-associated risk.
[b]A VCTE threshold matching the LRE risk of ELF ≥12.80 was not identified. 75 kPa reflects the upper limit of the analytical range for LSM by VCTE.
[c]The LRE risk at VCTE ≥8.0 kPa is 4.0% (3.0%, 5.2%) and may be considered an alternate aligned cut-off for ELF ≥9.00.
[d]The LRE risk at VCTE ≥25.0 kPa is 9.9% (7.1%, 13.4%).

Table 3. Risk of liver-related clinical outcomes stratified by ELF and VCTE combinations.

Interpretation	ELF and VCTE relative to respective cut-offs	Total patients	Patients with events	LRE risk (95% CI)[a]	Likelihood ratio[b] (95% CI)	LRE incidence per 1,000 PY (95% CI)	Cox proportional hazard ratio (95% CI) [*p* value]
Agree below (↓)	ELF <9.80; VCTE <12.0 kPa	206	2	1.0% (0.1%–3.5%)	0.25 (0.06–0.98)	6.9 (0.0–17.3)	1.00 (Reference)
Disagree	ELF <9.80; VCTE ≥12.0 kPa	233	2	0.9% (0.1%–3.1%)	0.22 (0.06–0.87)	6.2 (0.0–15.6)	–
	ELF ≥9.80; VCTE <12.0 kPa	205	2	1.0% (0.1%–3.5%)	0.25 (0.06–0.98)	6.9 (0.0–17.3)	–
	Total	438	4	1.0% (0.1%–3.5%)	0.24 (0.09–0.61)	6.6 (1.6–13.2)	0.99 (0.18–5.42) [p = 0.9924]
Agree above (↑)	ELF ≥9.80; VCTE ≥12.0 kPa	871	51	5.9% (4.4%–7.6%)	1.59 (1.44–1.76)	45.7 (34.0–58.2)	6.59 (1.60–27.07) [p = 0.0089]
Agree below (↓)	ELF <10.50; VCTE <20.0 kPa	640	4	0.6% (0.2%–1.6%)	0.16 (0.06–0.41)	4.5 (1.1–8.9)	1.00 (Reference)
Disagree	ELF <10.50; VCTE ≥20.0 kPa	221	6	2.7% (1.0%–5.8%)	0.71 (0.33–1.54)	20.3 (6.8–37.2)	–
	ELF ≥10.50; VCTE <20.0 kPa	275	9	3.3% (1.5%–6.1%)	0.87 (0.47–1.59)	25.3 (11.2–42.2)	–
	Total	496	15	3.0% (1.7%–4.9%)	0.80 (0.51–1.24)	23.0 (12.3–35.3)	5.14 (1.71–15.48) [p = 0.0036]
Agree above (↑)	ELF ≥10.50; VCTE ≥20.0 kPa	379	38	10.0% (7.2%–13.5%)	2.85 (2.32–3.50)	82.0 (56.1–110.1)	18.12 (6.46–50.78) [p <0.0001]
Agree below (↓)	ELF <11.30; VCTE <30.0 kPa	1,113	15	1.3% (0.8%–2.2%)	0.35 (0.23–0.54)	9.9 (5.3–15.2)	1.00 (Reference)
Disagree	ELF <11.30; VCTE ≥30.0 kPa	128	7	5.5% (2.2%–10.9%)	1.48 (0.72–3.02)	41.3 (11.8–76.7)	–
	ELF ≥11.30; VCTE <30.0 kPa	149	8	5.4% (2.3%–10.3%)	1.45 (0.75–2.81)	42.8 (16.1–74.9)	–
	Total	277	15	5.4% (3.1%–8.8%)	1.46 (0.94–2.29)	42.1 (22.5–64.6)	4.31 (2.11–8.81) [p <0.0001]
Agree above (↑)	ELF ≥11.30; VCTE ≥30.0 kPa	125	27	21.6% (14.7%–29.8%)	7.05 (5.05–9.84)	188.2 (118.5–264.9)	19.23 (10.22–36.18) [p <0.0001]

ELF, enhanced liver fibrosis; LRE, liver-related event; PY, patient-years; VCTE, vibration-controlled transient elastography.
p values for the respective ELF/VCTE cut-off pairings were: 9.80/12.0 kPa (p = 1.000), 10.50/20.0 kPa (p = 0.7966), and 11.30/30.0 kPa (p = 1.000).
[a]Fisher's exact test was used to compare LRE rates between discordant groups (VCTE < cut-off & ELF ≥ cut-off *vs.* ELF < cut-off & VCTE ≥ cut-off).
[b]LR = proportion of events in stratum / proportion of non-events in stratum.

stratification translated into improved model discrimination compared with guideline-based single-test approaches.

Model comparisons

In comparisons of LRE prognostication models, agreement-based combinations of ELF and VCTE demonstrated greater discrimination for LREs than either test alone (Table S11). While ELF alone demonstrated better discrimination than VCTE alone (C-index 0.758 *vs.* 0.708), the combination of ELF 11.30 and VCTE 30.0 kPa demonstrated the highest discrimination (C-index = 0.786) and best model fit (lowest AIC/BIC). Overall, these findings suggest that the combination of ELF and VCTE provides incremental value primarily at higher, concordant risk thresholds, where agreement between markers identifies a subgroup with more distinct risk stratification.

ROC analyses showed a similar pattern, with improved discrimination for the ELF/VCTE combination compared with models using either test alone at typical thresholds. In logistic regression-based step-function ROC analyses (Fig. 3A), the ELF/VCTE combination achieved a higher AUROC (0.779; 95% CI 0.714, 0.844) than VCTE alone (0.705; 95% CI 0.651, 0.760; DeLong p = 0.0129), while differences between the ELF/VCTE combination and ELF alone (AUROC 0.753; 95% CI 0.692, 0.815) were not statistically significant (p = 0.2632). Similar patterns were observed in Cox regression-based ROC analyses at 12 months (Fig. 3B), with the ELF/VCTE combination demonstrating the highest discrimination (AUROC 0.801; 95% CI 0.711, 0.848), compared with ELF (0.790; 95% CI 0.696, 0.825) and VCTE (0.694; 95% CI 0.650, 0.769).

Time-dependent ROC analyses further supported the discriminative utility of the ELF/VCTE combination over follow-up (Fig. S5; Table S12). Across evaluated time points from 3 to 12 months, AUC(t) values for the ELF/VCTE combination were significantly higher than those for VCTE alone (all p <0.01). Although AUC(t) values for the ELF/VCTE combination remained numerically higher than those for VCTE through later follow-up, these differences were not statistically significant beyond 1 year. Relative to ELF alone, AUC(t) values for the ELF/VCTE combination were comparable.

Collectively, these findings indicate that agreement-based combinations of ELF and VCTE maintain or improve discrimination for LREs relative to either test alone, particularly compared with VCTE, and complement the risk stratification patterns observed in concordance and time-to-event analyses.

Discussion

Our study generated several insights with potential implications for clinical practice. Current clinical guidelines recommend ELF and VCTE thresholds that are often interpreted as broadly equivalent across tests. However, these thresholds are based on independent studies, were typically validated against histological fibrosis stage and have not been explicitly calibrated to yield equivalent risk of LREs across tests. For example, commonly cited guidelines associate ELF 7.70 with VCTE 8.0 kPa, ELF 9.80 with VCTE 12.0 kPa, and ELF 11.30 with VCTE 20.0 kPa.[3,9,37,38] While our analyses confirm the pairing of ELF 9.80/VCTE 12.0 kPa, they suggest that the lower and upper thresholds may be more appropriately aligned as ELF 9.00/VCTE 8.0 kPa and ELF 11.30/VCTE 30.0 kPa, respectively. These findings indicate that risk-aligned

Table 4. Likelihood of histological progression and regression at 1 year stratified by ELF and VCTE combinations.

Interpretation	ELF and VCTE relative to respective cut-offs	Total patients	Patients with events	Event rate (95% CI)[a]	Likelihood ratio[b] (95% CI)
**Histological progression from F3 to F4[c]**					
Agree below (↓)	ELF <9.80; VCTE <12.0 kPa	178	4	2.2% (0.6%–5.7%)	0.13 (0.05–0.33)
Disagree	ELF <9.80; VCTE ≥12.0 kPa	145	23	15.9% (10.3%–22.8%)	1.03 (0.69–1.53)
	ELF ≥9.80; VCTE <12.0 kPa	146	19	13.0% (8.0%–19.6%)	0.82 (0.53–1.27)
	Total	291	42	14.4% (10.6%–19.0%)	0.92 (0.71–1.19)
Agree above (↑)	ELF ≥9.80; VCTE ≥12.0 kPa	271	69	25.5% (20.4%–31.1%)	1.86 (1.54–2.25)
Overall	—	740	115	15.5% (13.0%–18.4%)	—
**Histological regression from F4 to <F4[d]**					
Agree below (↓)	ELF <9.80; VCTE <12.0 kPa	58	33	56.9% (43.2%–69.8%)	5.82 (3.55–9.54)
Disagree	ELF <9.80; VCTE ≥12.0 kPa	139	37	26.6% (19.5 %–34.8%)	1.60 (1.14–2.25)
	ELF ≥9.80; VCTE <12.0 kPa	88	25	28.4% (19.3%–39.0%)	1.75 (1.13–2.70)
	Total	227	62	27.3% (21.6%–33.6%)	1.66 (1.30–2.11)
Agree above (↑)	ELF ≥9.80; VCTE ≥12.0 kPa	678	83	12.2% (9.9%–14.9%)	0.62 (0.52–0.72)
Overall	—	963	178	18.5% (16.1%–21.1%)	—

P values for the progression and regression analyses were $p = 0.5093$ and $p = 0.7625$, respectively.
[a]Fisher's exact test was used to compare rates of histological change between discordant groups (VCTE <12.0 kPa & ELF ≥9.80 *vs*. ELF <9.80 & VCTE ≥12.0 kPa).
[b]LR = proportion of events in stratum / proportion of non-events in stratum.
[c]Baseline: all patients staged as NASH CRN F3. Events: histological staging of F4 at Week 48.
[d]Baseline: all patients staged as NASH CRN F4. Events: histological staging of F0 to F3 at Week 48/52.

threshold pairings may enable more consistent prognostic assessment in patients with MASH.

While ELF ≥11.30 and VCTE ≥30.0 kPa were each individually associated with an event risk of approximately 13%, concordance at these thresholds was associated with a substantially higher risk of 21.6%. In contrast, when the tests were discordant, with one above and one below threshold, the event risk was lower, at 5.4%. This pattern suggests a synergistic rather than additive effect, likely due to ELF and VCTE capturing distinct but converging biological processes of matrix turnover and architectural distortion, respectively.[39] Discordance of ELF and VCTE may indicate an intermediate phase of fibroinflammatory injury with elevated near-term risk and likely reflects a clinically meaningful transitional state. Rather than defaulting to the "more severe" test or dismissing the discrepancy, clinicians could consider discordance itself as a marker of elevated risk that warrants closer monitoring or further evaluation. Although patients in this putative transitional phase may be ideal candidates for treatment, recent recommendations exclude patients with either ELF ≥11.30 or VCTE ≥20.0 kPa from treatments indicated for patients with F2/F3 fibrosis.[40,41] Analyses from ongoing phase III studies evaluating clinical and liver-related outcomes will likely be needed to determine whether patients with discordant ELF and VCTE measurements respond to MASH therapeutics.

The biochemical analysis of discordant ELF and VCTE results provides additional insight into the potential biological basis of risk stratification. Although patients with ELF <11.30/VCTE ≥30.0 kPa and ELF ≥11.30/VCTE <30.0 kPa had nearly identical LRE risks, their biochemical profiles were not identical. Patients with ELF ≥11.30/VCTE <30.0 kPa had significantly higher AST and ALT concentrations, and modestly higher ALP concentrations, than patients with ELF <11.30/VCTE ≥30.0 kPa, whereas platelet count, GGT, and bilirubin levels were comparable between discordant groups. These findings suggest that patients identified as high-risk by ELF alone may exhibit greater evidence of ongoing hepatocellular injury despite having LSM below the highest-risk threshold. Conversely, patients identified as high-risk by VCTE alone may have comparable clinical risk despite less pronounced biochemical evidence of liver injury. Together, these observations support the concept that ELF and VCTE capture partially overlapping but distinct biological dimensions of advanced liver disease and provide complementary prognostic information when interpreted together.

A similar pattern was observed when the aligned cut-off framework was evaluated against histological progression and regression using lower aligned thresholds of ELF 9.80 and VCTE 12.0 kPa. Among patients with baseline F3 fibrosis, concordantly elevated ELF and VCTE values were associated with the highest likelihood of progression to F4, whereas concordantly low results identified patients with the lowest likelihood of progression. Conversely, among patients with baseline F4 fibrosis, concordantly low ELF and VCTE values were associated with the greatest likelihood of regression to <F4, while concordantly elevated values identified patients least likely to regress. Notably, rates of histological change were similar between discordant strata, regardless of whether ELF or VCTE exceeded the threshold. The consistency of concordance patterns across both histological and clinical endpoints suggests that the relationship between ELF/VCTE agreement and risk reflects a broader biological continuum of disease severity and progression rather than an association specific to a single outcome measure. Together, these findings suggest that agreement between ELF and VCTE reflects underlying disease biology, and that discordance may represent an intermediate disease state characterized by neither the low-risk profile associated with concordantly negative results nor the high-risk profile associated with concordantly positive results.

Although this dataset included a high proportion of patients with F4 fibrosis (56%), the findings from this study have important implications for the design of non-cirrhotic MASH clinical trials. Natural history studies suggest that the expected rate of decompensation in patients with F2 or F3 fibrosis is approximately 2% over 4 years.[26] Given that current MASH therapeutic trials typically span 4-5 years, most primary endpoints are likely to reflect progression to cirrhosis.[42–44] As a result, despite efforts to replace histological endpoints with non-invasive alternatives

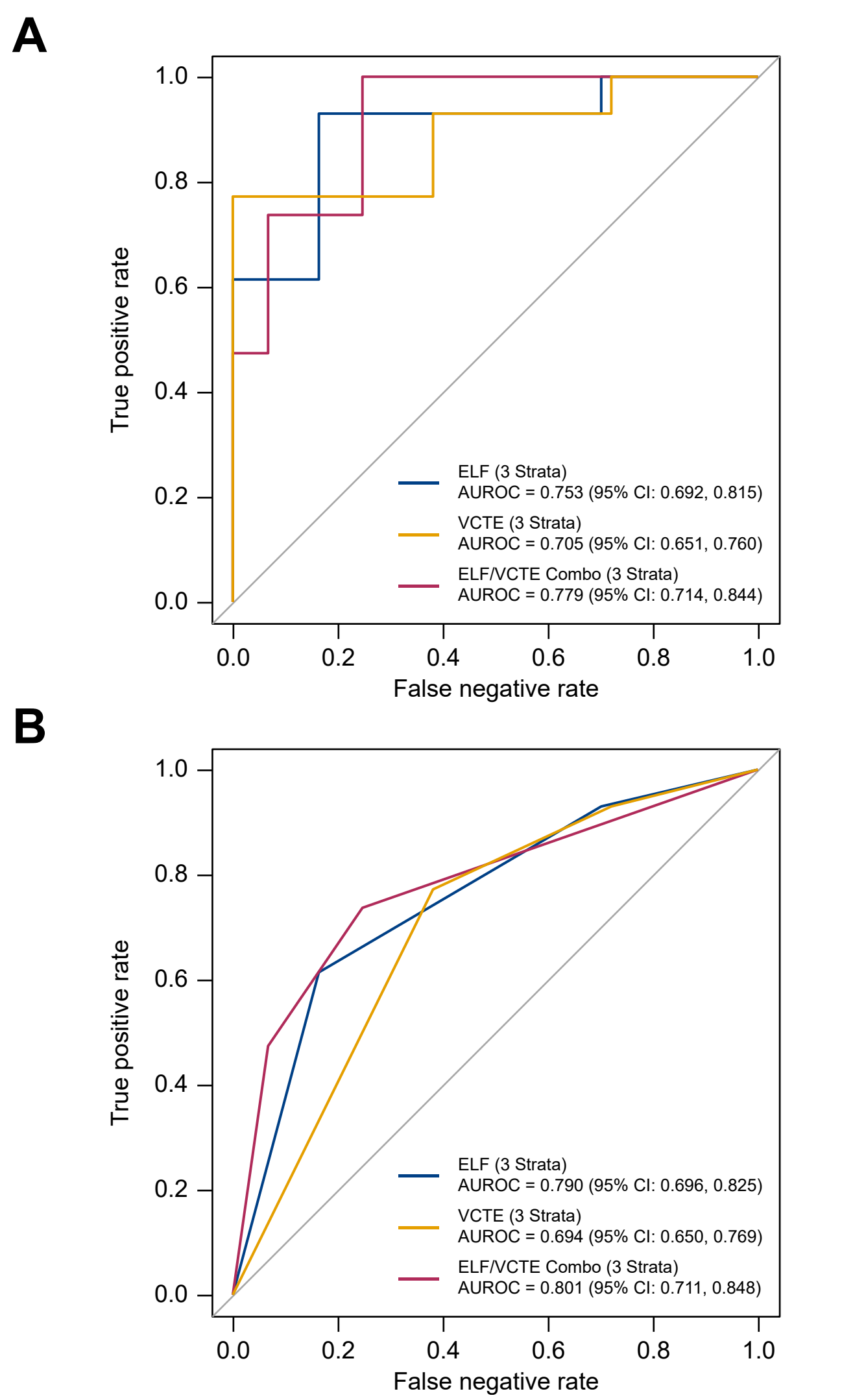

Fig. 3. Receiver-operating characteristic curves by measurement of baseline ELF (stratified by cut-offs at 9.80 and 11.30), VCTE (stratified by cut-offs at 12.0 and 20.0 kPa), or ELF and VCTE combination (agreement-based stratification using 11.30 and 30.0 kPa cut-offs). (A) Logistic regression-based step-function ROC curves. *p* values of DeLong's test: ELF *vs*. VCTE (*p* = 0.1448), ELF/VCTE Combo *vs*. ELF (*p* = 0.2632), ELF/VCTE Combo *vs*. VCTE (*p* = 0.0129). (B) Cox regression-based ROC curves at 12 months. AUROC, area under the ROC curve; ELF, enhanced liver fibrosis; ROC, receiver-operating characteristic; VCTE, vibration-controlled transient elastography.

for accelerated approval, traditional approval will likely continue to rely on histologically confirmed progression unless a validated surrogate endpoint is qualified.[45]

The US FDA has indicated that specific NITs, or combinations thereof, may qualify as surrogate endpoints if highly predictive of clinical outcomes.[45] Our study provides evidence that ELF ≥11.30 and VCTE ≥30.0 kPa are individually associated with more than double the risk of LREs compared with histologically staged F4, and that concordance at these thresholds is associated with nearly threefold higher risk. This suggests that the combination of ELF and VCTE at these thresholds represents a potential candidate for a validated surrogate endpoint, while also reducing potential analytical noise associated with false results from a single test. Notably, even discordant results at these thresholds confer a risk comparable to histological F4, offering additional reassurance that this combination identifies a clinically meaningful high-risk population. Further studies are warranted to determine whether this combination can support biomarker qualification as a validated surrogate endpoint.

In this study, we evaluated a VCTE threshold of 30.0 kPa, extending beyond the highest guideline-recommended cut-off of 25.0 kPa proposed in the Baveno VII consensus to rule-in clinically significant portal hypertension. While a VCTE threshold of 25.0 kPa was associated with slightly lower LRE risk than a threshold of 30.0 kPa when used alone or in combination with ELF, these differences were not statistically significant. Importantly, when paired with an ELF threshold of 11.30, both VCTE cut-offs identified patients at similarly elevated risk, suggesting that 25.0 kPa represents an acceptable threshold for clinical application within combined ELF/VCTE risk stratification frameworks. However, the use of 30.0 kPa provided greater separation of prognostic risk strata, supporting its use when the objective is greater discrimination of advanced risk rather than alignment with clinically significant portal hypertension rule-in criteria alone.

There are some limitations to this study. The primary analyses in this study were limited to ELF and VCTE, which were selected based on data availability and their prominent inclusion in current clinical practice guidance.[3,9] Although a growing number of newer non-invasive markers and composite scores (*e.g*. FAST, MAST, Agile scores, NIS4/NIS2+) have been developed,[46–49] these were not available for inclusion in the present dataset. Future studies incorporating both established and emerging NITs may further refine risk-aligned threshold combination strategies.

An additional limitation relates to the pooling of data from five clinical trials, including participants from both placebo and treatment arms. While this approach increased statistical power, it also introduced heterogeneity in the study design. Although no significant differences in LRE risk were observed across ELF strata between treatment and placebo arms and in other sensitivity analyses (Tables S13–S15), the potential influence of investigational therapies on disease course cannot be entirely excluded. Additionally, all biopsies were read by a single pathologist, which reduced inter-reader variability, but may have introduced interpretation bias.

It should also be recognized that the risk-aligned ELF and VCTE thresholds were derived empirically from observed event rates within this pooled clinical trial dataset. Although threshold-tolerance analyses suggested that the principal clinically relevant alignments were relatively stable across small differences in observed LRE risk, these analyses do not account for all sources of variability, including biological variability, analytical variability, measurement variability, or VCTE reliability characteristics. In particular, interquartile range-to-median ratio (IQR/M) data were not available for VCTE measurements. Consequently, the potential impact of LSM reliability on risk stratification and threshold alignment could not be assessed.

In this study, participants were enrolled through clinical trial eligibility criteria and therefore represent a selected population characterized by older age, advanced liver disease, and a high prevalence of advanced fibrosis. As a result, the observed risk relationships and threshold alignments may not be directly

applicable to younger patients, individuals with less advanced fibrosis, or routine clinical practice populations. The absence of an external validation cohort represents a limitation of the present study. Therefore, although the proposed risk-aligned thresholds were supported by internal consistency across analyses of LREs and histological change, they were derived from a selected advanced-fibrosis clinical trial population with a limited number of clinical events. Independent validation in external real-world and clinical trial cohorts will be necessary to establish their reproducibility, generalizability, and clinical utility.

Despite these limitations, this study demonstrates that ELF and VCTE cut-offs can be aligned to yield broadly comparable prognostic performance. Accordingly, modest refinement of guideline-recommended thresholds may be informative when these methods are interpreted in relation to one another for LRE risk staging, rather than as strictly diagnostic surrogates. In addition, our findings show that combining ELF and VCTE can improve prognostication of liver-related clinical outcomes, particularly in higher risk strata. Collectively, these results may support more consistent risk assessment in specialist settings and improve patient enrichment strategies in clinical trials of therapeutics for MASH.

Affiliations

[1]*Institute of Medical Science, Temerty Faculty of Medicine, University of Toronto, Toronto, Ontario, Canada;* [2]*Siemens Healthcare Diagnostics Inc., Tarrytown, New York, United States;* [3]*Billin Life Sciences Consulting, LLC, San Mateo, California, United States;* [4]*University of California School of Medicine, San Francisco, California, United States;* [5]*Division of Gastroenterology, Department of Medicine, Toronto Centre for Liver Disease, Toronto General Hospital, University Health Network, Toronto, Ontario, Canada;* [6]*Stravitz-Sanyal Institute for Liver Disease and Metabolic Health, Division of Gastroenterology, Hepatology and Nutrition, Virginia Commonwealth University School of Medicine, Richmond, Virginia, United States*

Abbreviations

AUROC, area under the ROC curve; ELF, enhanced liver fibrosis; LRE, liver-related event; LSM, liver stiffness measurement; MASH, metabolic dysfunction-associated steatohepatitis; MASLD, metabolic dysfunction-associated steatotic liver disease; NAFLD, nonalcoholic fatty liver disease; NASH CRN, Nonalcoholic Steatohepatitis Clinical Research Network; NITs, non-invasive tests; ROC, receiver-operating characteristic; VCTE, vibration-controlled transient elastography.

Financial support

This study was supported by Siemens Healthcare Diagnostics Inc. (USA) and by the Novo DevSTAR programme (Denmark).

Conflicts of interest

MG, DBC, RF, ED and RB are employees of Siemens Healthineers. ANB was an employee and is a stockholder of Gilead Sciences; KP is consultant for Novo, Merck, and Resalis, and has received grants from Novo. AJS has stock options in Durect, Inversago, Tiziana, Rivus, Exhalenz, Genfit. He has served as a consultant to Intercept, Gilead, Takeda, Meck, Eli Lilly, Novo, Astra Zeneca, Boehringer Ingelheim, Alnylam, Regeneron, Histoindex, Path AI, Pfizer, 89Bio, Altimmune, Northsea, Akero, Madrigal, Salix, Myovant, Poxel, Surrozen, Hanmi, Aligos, Promed, Zydus. His institution has received grants from Intercept, Novo, Eli Lilly, Boehringer Ingelheim, Echosens, Hanmi, Madrigal, Gilead, Salix, Meck, Takeda. He receives royalties from Elsevier and Wolter Kluwers. KP has served as a consultant to Gilead Sciences, Galectin Therapeutics, Novo, Resalis, Inipharm, and Merck. He has received grants from Novo, Madrigal, Gilead Sciences, Merck, GlaxoSmithKline, and 89bio.

Please refer to the accompanying ICMJE disclosure forms for further details.

Authors' contributions

MG: study concept and design, interpretation of data, and drafting of manuscript; ANB: study concept, review and editing of manuscript; DBC: interpretation of data, review and editing of manuscript; RF, ED and CB: statistical analysis, interpretation of data, review and editing of manuscript; AJS, JJF and KP: study supervision, data interpretation, critical revision of the manuscript.

Data availability

The data underlying this study were obtained under data sharing agreements and cannot be made publicly available by the authors. Access to the original datasets is subject to the approval and data-sharing policies of the respective data owners.

Supplementary data

Supplementary data to this article can be found online at https://doi.org/10.1016/j.jhepr.2026.102041.

References

Author names in bold designate shared co-first authorship

[1] Setiawan VW, Stram DO, Porcel J, et al. Prevalence of chronic liver disease and cirrhosis by underlying cause in understudied ethnic groups: the multiethnic cohort. Hepatology Dec 2016;64(6):1969–1977. https://doi.org/10.1002/hep.28677.

[2] Younossi Z, Anstee QM, Marietti M, et al. Global burden of NAFLD and NASH: trends, predictions, risk factors and prevention. Nat Rev Gastroenterol Hepatol Jan 2018;15(1):11–20. https://doi.org/10.1038/nrgastro.2017.109.

[3] European Association for the Study of the Liver (EASL). European association for the study of diabetes (EASD), European association for the study of Obesity (EASO). EASL-EASD-EASO clinical practice guidelines on the management of metabolic dysfunction-associated steatotic liver disease (MASLD). J Hepatol Sep 2024;81(3):492–542. https://doi.org/10.1016/j.jhep.2024.04.031.

[4] Younossi ZM, Golabi P, de Avila L, et al. The global epidemiology of NAFLD and NASH in patients with type 2 diabetes: a systematic review and meta-analysis. J Hepatol Oct 2019;71(4):793–801. https://doi.org/10.1016/j.jhep.2019.06.021.

[5] Younossi ZM, Golabi P, Price JK, et al. The global epidemiology of nonalcoholic fatty liver disease and nonalcoholic steatohepatitis among patients with type 2 diabetes. Clin Gastroenterol Hepatol Mar 21 2024;22(10):1999–2010.e8. https://doi.org/10.1016/j.cgh.2024.03.006.

[6] **Wong VW**, **Ekstedt M**, Wong GL, et al. Changing epidemiology, global trends and implications for outcomes of NAFLD. J Hepatol Sep 2023;79(3):842–852. https://doi.org/10.1016/j.jhep.2023.04.036.

[7] Singh S, Allen AM, Wang Z, et al. Fibrosis progression in nonalcoholic fatty liver vs nonalcoholic steatohepatitis: a systematic review and meta-analysis of paired-biopsy studies. Clin Gastroenterol Hepatol Apr 2015;13(4):643–654. https://doi.org/10.1016/j.cgh.2014.04.014. e1-9; quiz e39-40.

[8] Younossi ZM, Stepanova M, Ong J, et al. Nonalcoholic steatohepatitis is the most Rapidly increasing indication for liver transplantation in the United States. Clin Gastroenterol Hepatol Mar 2021;19(3):580–589 e5. https://doi.org/10.1016/j.cgh.2020.05.064.

[9] Rinella ME, Neuschwander-Tetri BA, Siddiqui MS, et al. AASLD Practice Guidance on the clinical assessment and management of nonalcoholic fatty liver disease. Hepatology May 1 2023;77(5):1797–1835. https://doi.org/10.1097/HEP.0000000000000323.

[10] Lazarus JV, Mark HE, Anstee QM, et al. Advancing the global public health agenda for NAFLD: a consensus statement. Nat Rev Gastroenterol Hepatol Jan 2022;19(1):60–78. https://doi.org/10.1038/s41575-021-00523-4.

[11] Srivastava A, Gailer R, Tanwar S, et al. Prospective evaluation of a primary care referral pathway for patients with non-alcoholic fatty liver disease. J Hepatol Aug 2019;71(2):371–378. https://doi.org/10.1016/j.jhep.2019.03.033.

[12] Day J, Patel P, Parkes J, et al. Derivation and performance of standardized enhanced liver fibrosis (ELF) test thresholds for the detection and Prognosis

of liver fibrosis. J Appl Lab Med Mar 2019;3(5):815–826. https://doi.org/10.1373/jalm.2018.027359.
[13] Mozes FE, Lee JA, Selvaraj EA, et al. Diagnostic accuracy of non-invasive tests for advanced fibrosis in patients with NAFLD: an individual patient data meta-analysis. Gut May 2022;71(5):1006–1019. https://doi.org/10.1136/gutjnl-2021-324243.
[14] Patel K, Asrani SK, Fiel MI, et al. Accuracy of blood-based biomarkers for staging liver fibrosis in chronic liver disease: a systematic review supporting the AASLD Practice Guideline. Hepatology Jan 1 2025;81(1):358–379. https://doi.org/10.1097/HEP.0000000000000842.
[15] Lijmer JG, Bossuyt PM, Heisterkamp SH. Exploring sources of heterogeneity in systematic reviews of diagnostic tests. Stat Med Jun 15 2002;21(11):1525–1537. https://doi.org/10.1002/sim.1185.
[16] Macaskill P, Gatsonis C, Deeks JJ, et al. Chapter 10: Analysing and presenting results. In: Deeks JJ, Bossuyt PM, Gatsonis C, editors. Cochrane Handbook for systematic reviews of diagnostic test Accuracy. The Cochrane Collaboration; 2010. Version 1.0.
[17] Sanyal AJ, Harrison SA, Ratziu V, et al. The natural history of advanced fibrosis due to nonalcoholic steatohepatitis: data from the simtuzumab trials. Hepatology Dec 2019;70(6):1913–1927. https://doi.org/10.1002/hep.30664.
[18] Are VS, Vuppalanchi R, Vilar-Gomez E, et al. Enhanced liver fibrosis score can Be used to predict liver-related events in patients with nonalcoholic steatohepatitis and compensated cirrhosis. Clin Gastroenterol Hepatol Jun 2021;19(6):1292–1293 e3. https://doi.org/10.1016/j.cgh.2020.06.070.
[19] Younossi ZM, Anstee QM, Wai-Sun Wong V, et al. The association of histologic and noninvasive tests with Adverse clinical and patient-reported outcomes in patients with advanced fibrosis due to nonalcoholic steatohepatitis. Gastroenterology Apr 2021;160(5):1608–1619 e13. https://doi.org/10.1053/j.gastro.2020.12.003.
[20] Johnson AL, Hayward KL, Patel P, et al. Predicting liver-related outcomes in People with nonalcoholic fatty liver disease: the prognostic value of noninvasive fibrosis tests. Hepatol Commun Apr 2022;6(4):728–739. https://doi.org/10.1002/hep4.1852.
[21] Sanyal AJ, Anstee QM, Trauner M, et al. Cirrhosis regression is associated with improved clinical outcomes in patients with nonalcoholic steatohepatitis. Hepatology May 2022;75(5):1235–1246. https://doi.org/10.1002/hep.32204.
[22] Loomba R, Huang DQ, Sanyal AJ, et al. Liver stiffness thresholds to predict disease progression and clinical outcomes in bridging fibrosis and cirrhosis. Gut Mar 2023;72(3):581–589. https://doi.org/10.1136/gutjnl-2022-327777.
[23] Mozes FE, Lee JA, Vali Y, et al. Performance of non-invasive tests and histology for the prediction of clinical outcomes in patients with non-alcoholic fatty liver disease: an individual participant data meta-analysis. Lancet Gastroenterol Hepatol Aug 2023;8(8):704–713. https://doi.org/10.1016/S2468-1253(23)00141-3.
[24] Angulo P, Kleiner DE, Dam-Larsen S, et al. Liver fibrosis, but No other histologic features, is associated with Long-term outcomes of patients with nonalcoholic fatty liver disease. Gastroenterology Aug 2015;149(2):389–97 e10. https://doi.org/10.1053/j.gastro.2015.04.043.
[25] **Hagstrom H**, **Nasr P**, Ekstedt M, et al. Fibrosis stage but not NASH predicts mortality and time to development of severe liver disease in biopsy-proven NAFLD. J Hepatol Dec 2017;67(6):1265–1273. https://doi.org/10.1016/j.jhep.2017.07.027.
[26] Sanyal AJ, Van Natta ML, Clark J, et al. Prospective study of outcomes in Adults with nonalcoholic fatty liver disease. N Engl J Med Oct 21 2021;385(17):1559–1569. https://doi.org/10.1056/NEJMoa2029349.
[27] Harrison SA, Dubourg J. Liver biopsy evaluation in MASH drug development: Think thrice, act wise. J Hepatol 2024;81(5):886–894. https://doi.org/10.1016/j.jhep.2024.06.008.
[28] Chalasani N, Abdelmalek MF, Garcia-Tsao G, et al. Effects of belapectin, an inhibitor of Galectin-3, in patients with nonalcoholic steatohepatitis with cirrhosis and portal hypertension. Gastroenterology Apr 2020;158(5):1334–1345 e5. https://doi.org/10.1053/j.gastro.2019.11.296.
[29] Harrison SA, Abdelmalek MF, Caldwell S, et al. Simtuzumab is Ineffective for patients with bridging fibrosis or compensated cirrhosis caused by nonalcoholic steatohepatitis. Gastroenterology Oct 2018;155(4):1140–1153. https://doi.org/10.1053/j.gastro.2018.07.006.
[30] Harrison SA, Wong VW, Okanoue T, et al. Selonsertib for patients with bridging fibrosis or compensated cirrhosis due to NASH: results from randomized phase III STELLAR trials. J Hepatol Jul 2020;73(1):26–39. https://doi.org/10.1016/j.jhep.2020.02.027.
[31] Loomba R, Noureddin M, Kowdley KV, et al. Combination therapies including Cilofexor and Firsocostat for bridging fibrosis and cirrhosis Attributable to NASH. Hepatology Feb 2021;73(2):625–643. https://doi.org/10.1002/hep.31622.
[32] Anstee QM, Lawitz EJ, Alkhouri N, et al. Noninvasive tests accurately identify advanced fibrosis due to NASH: baseline data from the STELLAR trials. Hepatology Nov 2019;70(5):1521–1530. https://doi.org/10.1002/hep.30842.
[33] Sterling RK, Lissen E, Clumeck N, et al. Development of a simple noninvasive index to predict significant fibrosis in patients with HIV/HCV coinfection. Hepatology Jun 2006;43(6):1317–1325. https://doi.org/10.1002/hep.21178.
[34] Connoley D, Patel PJ, Hogan B, et al. The Enhanced Liver Fibrosis test maintains its diagnostic and prognostic performance in alcohol-related liver disease: a cohort study. BMC Gastroenterol Jun 28 2021;21(1):268. https://doi.org/10.1186/s12876-021-01795-5.
[35] Gee MFW, Fonstad R, De Vol EB, et al. Multi-threshold analysis of the ELF Test versus histology for prognostication of disease progression and regression in MASH. Hepatol Commun May 2026;10(5):e0931.
[36] de Franchis R, Bosch J, Garcia-Tsao G, et al. Baveno VII - Renewing consensus in portal hypertension. J Hepatol Apr 2022;76(4):959–974. https://doi.org/10.1016/j.jhep.2021.12.022.
[37] Cusi K, Isaacs S, Barb D, et al. American association of clinical Endocrinology clinical practice guideline for the diagnosis and management of nonalcoholic fatty liver disease in primary care and Endocrinology clinical settings: Co-Sponsored by the American association for the study of liver diseases (AASLD). Endocr Pract May 2022;28(5):528–562. https://doi.org/10.1016/j.eprac.2022.03.010.
[38] **Long MT**, **Noureddin M**, Lim JK. AGA clinical practice Update: diagnosis and management of nonalcoholic fatty liver disease in lean individuals: Expert review. Gastroenterology Sep 2022;163(3):764–774 e1. https://doi.org/10.1053/j.gastro.2022.06.023.
[39] Patel K, Sebastiani G. Limitations of non-invasive tests for assessment of liver fibrosis. JHEP Rep Apr 2020;2(2):100067. https://doi.org/10.1016/j.jhepr.2020.100067.
[40] Chen VL, Morgan TR, Rotman Y, et al. Resmetirom therapy for metabolic dysfunction-associated steatotic liver disease: October 2024 updates to AASLD Practice Guidance. Hepatology Jan 1 2025;81(1):312–320. https://doi.org/10.1097/HEP.0000000000001112.
[41] Bansal MB, Patton H, Morgan TR, et al. Semaglutide therapy for metabolic dysfunction-associated steatohepatitis: November 2025 updates to AASLD Practice Guidance. Hepatology Nov 7 2025. https://doi.org/10.1097/HEP.0000000000001608.
[42] Sanyal AJ, Newsome PN, Kliers I, et al. Phase 3 trial of Semaglutide in metabolic dysfunction-associated steatohepatitis. N Engl J Med Jun 5 2025;392(21):2089–2099. https://doi.org/10.1056/NEJMoa2413258.
[43] Harrison SA, Bedossa P, Guy CD, et al. A phase 3, randomized, controlled trial of Resmetirom in NASH with liver fibrosis. N Engl J Med Feb 8 2024;390(6):497–509. https://doi.org/10.1056/NEJMoa2309000.
[44] Anania FA, Dimick-Santos L, Mehta R, et al. Nonalcoholic steatohepatitis: current Thinking from the Division of Hepatology and Nutrition at the Food and drug Administration. Hepatology May 2021;73(5):2023–2027. https://doi.org/10.1002/hep.31687.
[45] Anania FA, Hager R, Higgins K, et al. Non-invasive tests: Establishing efficacy for metabolic dysfunction-associated steatohepatitis beyond the biopsy-current perspectives from the Division of Hepatology and Nutrition, US Food and drug Administration. Hepatology Aug 26 2025;84(1):321–327. https://doi.org/10.1097/HEP.0000000000001509.
[46] **Harrison SA**, **Ratziu V**, Magnanensi J, et al. NIS2+, an optimisation of the blood-based biomarker NIS4(R) technology for the detection of at-risk NASH: a prospective derivation and validation study. J Hepatol Sep 2023;79(3):758–767. https://doi.org/10.1016/j.jhep.2023.04.031.
[47] Newsome PN, Sasso M, Deeks JJ, et al. FibroScan-AST (FAST) score for the non-invasive identification of patients with non-alcoholic steatohepatitis with significant activity and fibrosis: a prospective derivation and global validation study. Lancet Gastroenterol Hepatol Apr 2020;5(4):362–373. https://doi.org/10.1016/S2468-1253(19)30383-8.
[48] Noureddin M, Truong E, Gornbein JA, et al. MRI-based (MAST) score accurately identifies patients with NASH and significant fibrosis. J Hepatol Apr 2022;76(4):781–787. https://doi.org/10.1016/j.jhep.2021.11.012.
[49] Sanyal AJ, Foucquier J, Younossi ZM, et al. Enhanced diagnosis of advanced fibrosis and cirrhosis in individuals with NAFLD using FibroScan-based Agile scores. J Hepatol Feb 2023;78(2):247–259. https://doi.org/10.1016/j.jhep.2022.10.034.

Keywords: Enhanced liver fibrosis (ELF) test; Fibrosis-4 (FIB-4); Steatotic liver disease (SLD); Metabolic dysfunction-associated steatotic liver disease (MASLD); Nonalcoholic fatty liver disease (NAFLD); Metabolic dysfunction-associated steatohepatitis (MASH); Nonalcoholic steatohepatitis (NASH); Immunoassay; Liver-related event (LRE); Liver stiffness measurement (LSM); Vibration-controlled transient elastography (VCTE).

Received 7 May 2026; received in revised form 4 September 2026; accepted 15 September 2026; Available online 18 September 2026

Journal of Hepatology, Volume *8*

Supplemental information

Aligned ELF and VCTE cut-offs facilitate risk staging in patients with metabolic dysfunction-associated steatohepatitis

Matthew F.W. Gee, Andrew N. Billin, Donald B. Chalfin, Rachel Fonstad, Edward B. De Vol, Jordan J. Feld, Keyur Patel, and Arun J. Sanyal

Aligned ELF and VCTE cutoffs facilitate risk staging in patients with metabolic dysfunction-associated steatohepatitis

Matthew F.W. Gee, Andrew N. Billin, Donald B. Chalfin, Rachel Fonstad, Edward B. De Vol, Jordan J. Feld, Keyur Patel, Arun J. Sanyal

Table of Contents

1. Study Population and Baseline Characteristics 2
2. Clinical Outcomes 5
3. Prognostic Alignment 6
   3.1 Methodology to Derive Equivalent Thresholds with Respect to LRE Risk 6
4. NIT Combinations....................12
5. Model Comparisons 19
6. Sensitivity Analyses....................23
7. Diagnostic Analyses 25
8. TRIPOD Statement Checklist 32
9. References....................36

1. Study Population and Baseline Characteristics

Fig. S1. Exclusions from liver-related event prognostic analyses

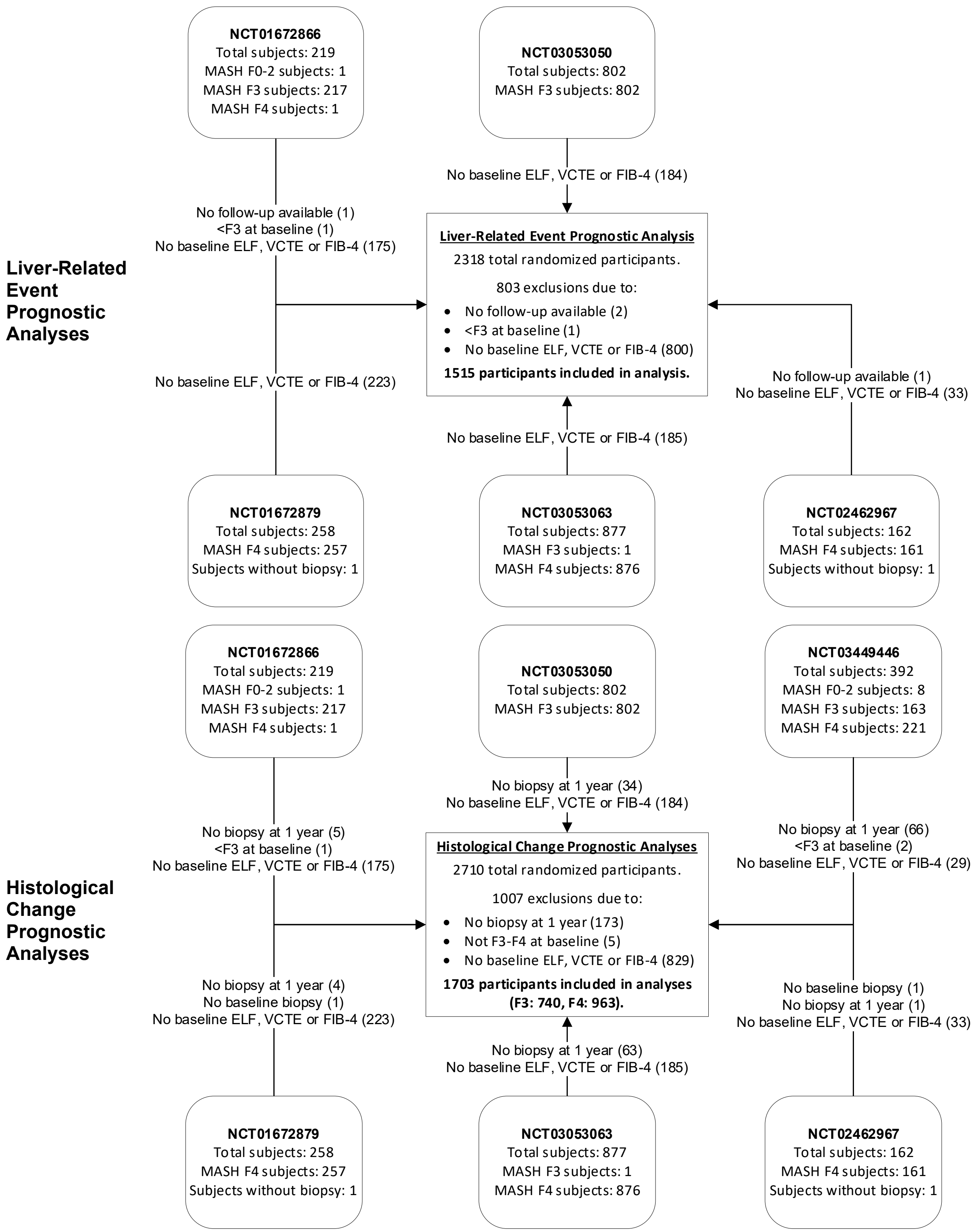

Table S1. Description of clinical trial datasets used for analyses

**Study Identifiers**	NCT01672866<br>GS-US-321-0105	NCT03053050<br>STELLAR-3	NCT01672879<br>GS-US-321-0106	NCT03053063<br>STELLAR-4	NCT02462967<br>NASH-CX	NCT03449446<br>ATLAS[a]
**Patient Population**	Bridging fibrosis (F3) due to MASH	Bridging fibrosis (F3) due to MASH	Compensated cirrhosis (F4) due to MASH	Compensated cirrhosis (F4) due to MASH	Compensated cirrhosis (F4) due to MASH	Bridging fibrosis (F3) & compensated cirrhosis (F4) due to MASH
**Phase**	Phase 2b	Phase 3	Phase 2b	Phase 3	Phase 2b	Phase 2b
**Study Drug**	Simtuzumab (Sim)	Selonsertib (Sel)	Simtuzumab (Sim)	Selonsertib (Sel)	Belapectin (Bel)	Selonsertib (Sel)<br>Firsocostat (Fir)<br>Cilofexor (Cilo)
**Randomization**	1:1:1<br>Sim 75 mg<br>Sim 125 mg<br>Placebo	2:2:1<br>Sel 6 mg<br>Sel 18 mg<br>Placebo	1:1:1<br>Sim 200 mg<br>Sim 700 mg<br>Placebo	2:2:1<br>Sel 6 mg<br>Sel 18 mg<br>Placebo	1:1:1<br>Bel 2 mg/kg<br>Bel 8 mg/kg<br>Placebo	1:1:1:2:2:2:1<br>Sel 18 mg<br>Fir 20 mg<br>Cilo 30 mg<br>Fir/Sel<br>Cilo/Sel<br>Cilo/Fir<br>Placebo
**Total Subjects in Study**	219	802	258	877	162	392
**Study Dates**	2012-2016	2017-2019	2012-2017	2017-2019	2015-2017	2018-2019
**Study Setting / Locations**	Refer to clinicaltrials.gov for full details					
**Eligibility Criteria**	Refer to clinicaltrials.gov for full details					
**Demographics**	Described in Harrison et al. (2018)[3] and Sanyal et al. (2019)[4]	Described in Harrison et al. (2020)[5] and Younossi et al. (2021)[6]	Described in Harrison et al. (2018),[3] Sanyal et al. (2019)[4] and Sanyal et al. (2022)[7]	Described in Harrison et al. (2020),[5] Younossi et al. (2021)[6] and Sanyal et al. (2022)[7]	Described in Chalasani et al. (2020)[8] and Are et al. (2021)[9]	Described in Loomba et al. (2021)[10]

[a] This dataset was not used for the liver-related event prognostic analyses.

Table S2. Distributions of VCTE and FIB-4 measurements at baseline arranged by ELF strata

ELF Score	N	VCTE (kPa)		FIB-4	
		Mean (SD)	Median (IQR)	Mean (SD)	Median (IQR)
<9.00	127	14.3 (9.5)	12.0 (8.9, 15.3)	1.37 (0.97)	1.20 (0.88, 1.58)
≥9.00 to <9.80	312	14.6 (8.1)	12.4 (9.5, 17.3)	1.71 (1.14)	1.46 (1.08, 2.06)
≥9.80 to <10.50	422	18.4 (11.1)	15.8 (11.4, 21.8)	2.18 (1.05)	2.00 (1.43, 2.67)
≥10.50 to <11.30	380	22.1 (12.5)	19.2 (14.3, 26.3)	3.02 (1.69)	2.64 (1.89, 3.64)
≥11.30 to <12.00	180	29.4 (17.1)	26.3 (16.8, 35.5)	3.87 (1.73)	3.49 (2.58, 4.97)
≥12.00 to <12.80	75	35.1 (17.6)	31.6 (21.3, 47.2)	4.79 (2.40)	4.36 (3.14, 6.12)
≥7.70	1511	20.6 (13.7)	16.8 (11.6, 25.4)	2.60 (1.72)	2.17 (1.44, 3.24)
≥8.30	1494	20.7 (13.7)	16.8 (11.7, 25.4)	2.62 (1.72)	2.19 (1.45, 3.26)
≥9.00	1388	21.2 (13.8)	17.3 (11.9, 26.0)	2.71 (1.73)	2.29 (1.52, 3.34)
≥9.80	1076	23.1 (14.5)	19.3 (13.3, 27.0)	3.00 (1.76)	2.58 (1.79, 3.65)
≥10.50	654	26.2 (15.7)	21.6 (15.4, 32.0)	3.52 (1.92)	3.09 (2.21, 4.36)
≥11.30	274	31.8 (17.8)	27.0 (18.7, 39.7)	4.22 (2.00)	3.81 (2.78, 5.29)
≥12.00	94	36.4 (18.2)	32.6 (21.3, 48.6)	4.89 (2.29)	4.38 (3.30, 6.13)
≥12.80	19	41.1 (20.3)	38.5 (25.9, 51.2)	5.31 (1.82)	4.41 (3.93, 6.79)

2. Clinical Outcomes

Table S3. Liver-related clinical events observed during patient follow-up

**Description of First Recorded Liver-Related Event**	**Number of Subjects with Liver-Related Clinical Outcome as a First Recorded Event[a]**
Clinically apparent ascites	16
Hepatic encephalopathy	12
Portal hypertension-related gastrointestinal events	20
Progression from small to medium or large varices	8
Newly diagnosed varies in those without prior varices	5
Esophageal variceal bleeding	1
Upper gastrointestinal bleeding	5
Gastropathy hemorrhage	1
Qualification for liver transplant (MELD Score ≥15)	4
≥2 increase in CPT Score	1
Liver-related death	0
Liver transplantation	1
Other decompensation event (description not available)	3
**Total**	**57**

[a] Only the first recorded clinical outcome was used in the analysis. Subsequent events occurring for these individuals was not considered.

3. Prognostic Alignment

3.1 Methodology to Derive Equivalent Thresholds with Respect to LRE Risk

All calculations were performed using R version 4.4.2. The following presents the workflow to empirically derive VCTE thresholds aligned in terms of LRE risk to established ELF cutoffs. The same approach was also used to derive ELF cutoffs from established VCTE thresholds, and to derive FIB-4 cutoffs from established ELF thresholds.

Step 1: Determine LRE Risk at Target ELF Cutoffs

Pre-specified ELF cutoffs were selected based on usage in guidelines and prior studies as follows: 7.70, 8.30, 9.00, 9.80, 10.50, 11.30, 12.00, 12.80.

$$c_{ELF} = \{7.70, 8.30, 9.00, 9.80, 10.50, 11.30, 12.00, 12.80\}$$

For each c_{ELF}, the observed risk of liver-related events for each ELF cutoff was calculated as the proportion of participants at or above the cutoff who experienced an event, as follows:

$$\hat{p}_{ELF} = \frac{k_{ELF}}{n_{ELF}}$$

where k_{ELF} is the number participants with an ELF value ≥ c_{ELF} who experienced an LRE, and n_{ELF} is the number participants with an ELF value ≥ c_{ELF}.

Exact binomial 95% confidence intervals (CIs) were calculated using the binom.confint() function with the “exact” method.

Step 2: Generate Candidate VCTE Thresholds

All unique observed baseline VCTE values for use as candidate thresholds were generated by using the R function sort(unique()) function with decreasing = TRUE, and were defined by:

$$T_{VCTE} = \{VCTE_1, VCTE_2 \ldots VCTE_n\}$$

Step 3: Calculate Observed LRE Risk at Each Candidate VCTE Threshold

Analogous to Step 1, the observed risk of liver-related events for each candidate VCTE threshold was calculated as the proportion of participants at or above the threshold who experienced an event, as follows

$$\hat{p}_{VCTE} = \frac{k_{VCTE}}{n_{VCTE}}$$

Step 4: Map ELF Cutoffs to VCTE Threshold

For each c_{ELF}, the equivalent VCTE threshold was defined as the value where the observed LRE risk is closest that for ELF.

$$t^*_{VCTE} = \underset{t \in T_{VCTE}}{\operatorname{argmin}} |\, \hat{p}_{VCTE} - \hat{p}_{ELF}|$$

Step 5: Map Empirically Derived VCTE Cutoffs to Nearest Established Cutoff

To enhance clinical interpretability, each derived t^*_{VCTE} was mapped to the nearest established VCTE cutoff in the set of 5.0, 8.0, 10.0, 12.0, 15.0, 20.0, 25.0, and 30 kPa. (NOTE: The previously derived 30.7 kPa prognostic cutoff for VCTE was rounded down to 30.0 kPa to better reflect the rule-of-five framework established by Baveno VII.)[1,2]

Observed risks were recalculated for these mapped thresholds with 95% CIs derived using the exact binomial approach.

Table S4. Corresponding ELF thresholds to established VCTE cutoffs with respect to risk of liver-related events

<table>
<tr><th colspan="2">VCTE</th><th colspan="4">ELF</th></tr>
<tr><th>Established VCTE Cutoff (kPa)</th><th>LRE Risk (95% CI)</th><th>Empirically Derived ELF Cutoff</th><th>LRE Risk (95% CI)</th><th>Nearest ELF Cutoff in Clinical Use</th><th>LRE Risk (95% CI)</th></tr>
<tr><td>≥5.0</td><td>3.8% (2.9%, 4.9%)</td><td>≥8.30</td><td>3.8% (2.9%, 4.9%)</td><td>≥8.30</td><td>3.8% (2.9%, 4.9%)</td></tr>
<tr><td>≥8.0</td><td>4.0% (3.0%, 5.2%)</td><td>≥8.82</td><td>4.0% (3.0%, 5.2%)</td><td rowspan="2">≥9.00</td><td rowspan="2">4.1% (3.1%, 5.3%)</td></tr>
<tr><td>≥10.0</td><td>4.2% (3.1%, 5.4%)</td><td>≥9.14</td><td>4.2% (3.2%, 5.4%)</td></tr>
<tr><td>≥12.0</td><td>4.8% (3.6%, 6.2%)</td><td>≥9.64</td><td>4.8% (3.6%, 6.2%)</td><td rowspan="2">≥9.80</td><td rowspan="2">4.9% (3.7%, 6.4%)</td></tr>
<tr><td>≥15.0</td><td>5.7% (4.3%, 7.5%)</td><td>≥10.09</td><td>5.7% (4.3%, 7.5%)</td></tr>
<tr><td>≥20.0</td><td>7.3% (5.4%, 9.7%)</td><td>≥10.54</td><td>7.3% (5.4%, 9.7%)</td><td>≥10.50</td><td>7.2% (5.3%, 9.4%)</td></tr>
<tr><td>≥25.0</td><td>9.9% (7.1%, 13.4%)</td><td>≥10.98</td><td>9.9% (7.2%, 13.2%)</td><td>≥11.30</td><td>12.8% (9.1%, 17.3%)</td></tr>
</table>

LRE: liver-related event

Table S5. Corresponding FIB-4 thresholds to established ELF cutoffs with respect to risk of liver-related events

ELF		FIB-4			
**Established ELF Cutoff**	**LRE Risk (95% CI)**	**Empirically Derived Cutoff**	**LRE Risk (95% CI)**	**Nearest FIB-4 Cutoff in Clinical Use (kPa)**	**LRE Risk (95% CI)**
≥7.70	3.8% (2.9%, 4.9%)	≥0.50	3.8% (2.9%, 4.9%)	≥1.00	4.1% (3.1%, 5.3%)
≥8.30	3.8% (2.9%, 4.9%)	≥0.60	3.8% (2.9%, 4.9%)		
≥9.00	4.1% (3.1%, 5.3%)	≥1.00	4.1% (3.1%, 5.3%)		
≥9.80	4.9% (3.7%, 6.4%)	≥1.40	4.9% (3.7%, 6.4%)	≥1.30[a]	4.6% (3.5%, 6.0%)
≥10.50	7.2% (5.3%, 9.4%)	≥2.20	7.2% (5.4%, 9.4%)	≥2.00[b]	6.2% (4.7%, 8.1%)
≥11.30	12.8% (9.1%, 17.3%)	≥3.60	12.7% (9.1%, 17.0%)	≥3.48	11.9% (8.5%, 15.9%)
≥12.00	21.3% (13.5%, 30.9%)	≥6.30	21.3% (11.9%, 33.7%)	N/A	N/A
≥12.80	47.4% (24.4%, 71.1%)	≥13.60	50.0% (1.3%, 98.7%)	N/A	N/A

LRE: liver-related event

[a] The LRE risk at FIB-4 ≥1.67 is 5.5% (4.2%, 7.1%).

[b] The LRE risk at FIB-4 ≥2.67 is 8.3% (6.1%, 11.0%).

Table S6. Risk of LREs at multiple different thresholds

Test / Combination[a]	Total Patients	Patients with Events	Clinical Outcome Risk (95% CI)	Likelihood Ratio (95% CI)	LRE Incidence per 1000 PY (95% CI)
**Histology**					
NASH CRN F3	660	7	1.1% (0.4%, 2.2%)	0.27 (0.14, 0.55)	7.7 (2.2, 14.4)
NASH CRN F4	853	50	5.9% (4.4%, 7.7%)	1.59 (1.43, 1.77)	45.2 (33.5, 57.9)
Overall (F3 & F4)	1513	57	3.8% (2.9%, 4,9%)	---	28.4 (21.4, 35.8)
**ELF Alone**					
ELF <10.39[b]	793	9	1.1% (0.5%, 2.1%)	0.29 (0.16, 0.54)	8.2 (3.6, 13.6)
ELF ≥9.80	1076	53	4.9% (3.7%, 6.4%)	1.33 (1.22, 1.43)	37.8 (27.8, 48.5)
ELF ≥10.14[c]	870	51	5.9% (4.4%, 7.6%)	1.59 (1.44, 1.76)	45.8 (34.1, 58.3)
ELF ≥10.50	654	47	7.2% (5.3%, 9.4%)	1.98 (1.73, 2.27)	57.4 (41.5, 74.5)
ELF ≥11.30	274	35	12.8% (9.1%, 17.3%)	3.75 (2.96, 4.74)	105.9 (72.6, 142.3)
**VCTE Alone**					
VCTE <14.0 kPa[b]	563	6	1.1% (0.4%, 2.3%)	0.28 (0.13, 0.59)	7.6 (2.5, 14.0)
VCTE ≥12.0 kPa	1104	53	4.8% (3.6%, 6.2%)	1.29 (1.19, 1.39)	36.9 (27.1, 47.3)
VCTE ≥15.0 kPa	875	50	5.7% (4.3%, 7.5%)	1.55 (1.39, 1.73)	44.6 (33.0, 57.0)
VCTE ≥15.5 kPa[c]	836	49	5.9% (4.4%, 7.7%)	1.59 (1.42, 1.79)	45.9 (33.7, 59.0)
VCTE ≥20.0 kPa	600	44	7.3% (5.4%, 9.7%)	2.02 (1.73, 2.36)	57.9 (40.8, 75.1)
VCTE ≥25.0 kPa	382	38	9.9% (7.1%, 13.4%)	2.83 (2.30, 3.47)	80.3 (55.0, 107.8)
VCTE ≥30.0 kPa	253	34	13.4% (9.5%, 18.3%)	3.97 (3.11, 5.08)	108.7 (73.5, 147.0)
**ELF and VCTE Combination**					
ELF ≥9.80 & VCTE ≥12.0 kPa	871	51	5.9% (4.4%, 7.6%)	1.59 (1.44, 1.76)	45.7 (34.0, 58.2)
ELF ≥10.50 & VCTE ≥15.0 kPa	504	42	8.3% (6.1%, 11.1%)	2.33 (1.96, 2.76)	67.6 (48.3, 88.6)
ELF ≥10.50 & VCTE ≥20.0 kPa	379	38	10.0% (7.2%, 13.5%)	2.85 (2.32, 3.50)	82.0 (56.1, 110.1)
ELF ≥11.30 & VCTE ≥20.0 kPa	200	31	15.5% (10.8%, 21.3%)	4.69 (3.56, 6.19)	131.5 (89.1, 178.1)
ELF ≥11.30 & VCTE ≥25.0 kPa	157	29	18.5% (12.7%, 25.4%)	5.80 (4.28, 7.85)	160.6 (105.2, 221.5)
ELF ≥11.30 & VCTE ≥30.0 kPa	125	27	21.6% (14.7%, 29.8%)	7.05 (5.05, 9.84)	188.2 (118.5, 264.9)

[a] Rows shaded in blue and green demonstrate liver-related event risk comparable to NASH CRN stage F3 and F4, respectively across multiple risk metrics.

[b] Empirically derived ELF (<10.39) and VCTE (<14.0 kPa) thresholds with equivalent risk as NASH CRN stage F3.

[c] Empirically derived ELF (≥10.14) and VCTE (≥15.5 kPa) thresholds with equivalent risk as NASH CRN stage F4.

Table S7.
Comparison of liver-related event risks at 18 months for ELF and VCTE at established thresholds

ELF		VCTE	
**ELF Cutoff**	**Clinical Outcome Risk (95% CI)**	**VCTE Cutoff (kPa)**	**Clinical Outcome Risk (95% CI)**
≥8.30	12.1% (9.3%, 15.4%)	≥5.0	12.1% (9.3%, 15.4%)
≥9.00	12.9% (9.9%, 16.4%)	≥8.0	13.0% (10.0%, 16.5%)
		≥10.0	13.6% (10.4%, 17.4%)
≥9.80	15.8% (12.0%, 20.1%)	≥12.0	16.0% (12.2%, 20.4%)
≥10.50	24.6% (18.7%, 31.3%)	≥20.0	24.7% (18.6%, 31.7%)
≥11.30	37.6% (27.8%, 48.3%)	≥30.0	37.0% (27.1%, 47.7%)
≥12.00	62.5% (43.7%, 78.9%)	N/A	N/A
≥12.80	75.0% (42.8%, 94.5%)	N/A	N/A

18-month event risk was calculated with the following groups:
Patients with Clinical Outcome: number of patients with LRE ≤18 months
Patients without Clinical Outcome: number of patients without LRE and follow-up >18 months

4. NIT Combinations

Table S8. Laboratory characteristics of patients stratified by concordance / discordance of ELF and VCTE measurements

Interpretation	ELF and VCTE Relative to Respective Cutoffs	Platelets [count]		AST [IU/L]		ALT [IU/L]		GGT [IU/L]		ALP [IU/L]		Bilirubin [mg/dL]	
		Median (IQR)	Wilcoxon Rank Sum p-value	Median (IQR)	Wilcoxon Rank Sum p-value	Median (IQR)	Wilcoxon Rank Sum p-value	Median (IQR)	Wilcoxon Rank Sum p-value	Median (IQR)	Wilcoxon Rank Sum p-value	Median (IQR)	Wilcoxon Rank Sum p-value
Agree Below (↓)	ELF <11.30 VCTE <30.0 kPa	190.0 (147.0, 244.0)		41.5 (31.5, 55.7)		45.5 (32.0, 68.0)		58.0 (38.0, 101.0)		81.0 (67.0, 101.0)		0.6 (0.4, 0.8)	
Disagree	ELF <11.30 VCTE ≥30.0 kPa	153.0 (118.0, 219.8)	0.0823	47.2 (32.4, 62.2)	<0.0001	39.8 (29.9, 63.0)	0.0002	102.0 (62.5, 164.0)	0.9084	92.5 (76.8, 118.2)	0.0328	0.6 (0.4, 0.8)	0.3469
	ELF ≥11.30 VCTE <30.0 kPa	143.0 (110.0, 181.0)		61.0 (46.0, 79.0)		54.5 (38.0, 79.5)		98.0 (58.0, 185.0)		101.0 (82.0, 124.0)		0.6 (0.5, 0.9)	
Agree Above (↑)	ELF ≥11.30 VCTE ≥30.0 kPa	118.0 (97.0, 169.0)		59.0 (46.0, 82.0)		41.0 (32.0, 59.0)		126.0 (77.0, 227.0)		123.0 (90.0, 152.0)		0.8 (0.5, 1.1)	

Table S9. Risk of liver-related clinical outcomes stratified by ELF and VCTE combinations (alternate cutoff pairs)

Interpretation	ELF and VCTE Relative to Respective Cutoffs	Total Patients	Patients with Events	LRE Risk (95% CI)[a]	Interval Likelihood Ratio (95% CI)	LRE Incidence per 1000 PY (95% CI)	Cox Proportional Hazard Ratio (95% CI) [P-Value]
Agree Below (↓)	ELF <10.50; VCTE <15.0 kPa	490	2	0.4% (0.0%, 1.5%)	0.10 (0.03, 0.41)	2.9 (0.0, 7.2)	1.00 (Reference)
Disagree	ELF <10.50; VCTE ≥15.0 kPa	371	8	2.2% (0.9%, 4.2%)	0.56 (0.29, 1.08)	16.0 (6.0, 27.9)	---
	ELF ≥10.50; VCTE <15.0 kPa	150	5	3.3% (1.1%, 7.6%)	0.88 (0.38, 2.07)	25.3 (5.1, 50.5)	---
	Total	521	13	2.5% (1.3%, 4.2%)	0.65 (0.40, 1.06)	18.6 (8.6, 30.0)	6.35 (1.43, 28.15) [p=0.0149]
Agree Above (↑)	ELF ≥10.50; VCTE ≥15.0 kPa	504	42	8.3% (6.1%, 11.1%)	2.33 (1.96, 2.76)	67.6 (48.3, 88.6)	23.02 (5.57, 95.11) [p<0.0001]
Agree Below (↓)	ELF <11.30; VCTE <20.0 kPa	841	9	1.1% (0.5%, 2.0%)	0.28 (0.15, 0.50)	7.8 (3.5, 12.9)	1.00 (Reference)
Disagree	ELF <11.30; VCTE ≥20.0 kPa	400	13	3.2% (1.7%, 5.5%)	0.86 (0.53, 1.40)	24.8 (11.5, 40.1)	---
	ELF ≥11.30; VCTE <20.0 kPa	74	4	5.4% (1.5%, 13.3%)	1.46 (0.55, 3.86)	42.3 (10.6, 84.6)	---
	Total	474	17	3.6% (2.1%, 5.7%)	0.95 (0.63, 1.43)	27.5 (14.6, 42.1)	3.50 (1.56, 7.86) [p=0.0024]
Agree Above (↑)	ELF ≥11.30; VCTE ≥20.0 kPa	200	31	15.5% (10.8%, 21.3%)	4.69 (3.56, 6.19)	131.5 (89.1, 178.1)	17.07 (8.12, 35.88) [p<0.0001]
Agree Below (↓)	ELF <11.30; VCTE <25.0 kPa	1016	13	1.3% (0.7%, 2.2%)	0.33 (0.21, 0.54)	9.4 (4.3, 15.1)	1.00 (Reference)
Disagree	ELF <11.30; VCTE ≥25.0 kPa	225	9	4.0% (1.8%, 7.5%)	1.07 (0.58, 1.97)	30.8 (13.7, 51.3)	---
	ELF ≥11.30; VCTE <25.0 kPa	117	6	5.1% (1.9%, 10.8%)	1.38 (0.64, 3.01)	40.1 (13.4, 73.5)	---
	Total	342	15	4.4% (2.5%, 7.1%)	1.17 (0.75, 1.83)	33.9 (18.1, 52.0)	3.64 (1.73, 7.65) [p=0.0007]
Agree Above (↑)	ELF ≥11.30; VCTE ≥25.0 kPa	157	29	18.5% (12.7%, 25.4%)	5.80 (4.28, 7.85)	160.6 (105.2, 221.5)	17.50 (9.09, 33.69) [p<0.0001]

[a] Fisher's exact test was used to compare liver-related event rates between discordant groups (VCTE < cutoff & ELF ≥ cutoff vs. ELF < cutoff & VCTE ≥ cutoff). P-values for the respective ELF/VCTE cutoff pairings were: 10.50/15.0 kPa (p=0.5350), 11.30/20.0 kPa (p=0.3191), and 11.30/25.0 kPa (p=0.5925).

Table S10. Risk of liver-related clinical outcomes stratified by ELF, VCTE and FIB-4 combinations

Interpretation[a]	NIT Cutoffs	Total Patients	Patients with Events	LRE Risk (95% CI)[a]	Interval Likelihood Ratio (95% CI)	LRE Incidence per 1000 PY (95% CI)	Cox Proportional Hazard Ratio (95% CI) [P-Value]
All Below (↓)	ELF: 9.80 VCTE: 12.0 kPa FIB-4: 1.30	96	0	0.0% (0.0%, 3.8%)	0.00	0.0	0.00
Disagree	ELF: 9.80 VCTE: 12.0 kPa FIB-4: 1.30	618	6	1.0% (0.4%, 2.1%)	0.25 (0.12, 0.54)	6.9 (2.3, 12.7)	1.00 (Reference)
All Above (↑)	ELF: 9.80 VCTE: 12.0 kPa FIB-4: 1.30	801	51	6.4% (4.8%, 8.3%)	1.74 (1.57, 1.93)	50.1 (37.3, 63.8)	6.95 (2.98, 16.19) [p<0.0001]
All Below (↓)	ELF: 10.50 VCTE: 20.0 kPa FIB-4: 2.67	551	1	0.2% (0.0%, 1.0%)	0.05 (0.01, 0.32)	1.3 (0.0, 3.9)	1.00 (Reference)
Disagree	ELF: 10.50 VCTE: 20.0 kPa FIB-4: 2.67	698	24	3.4% (2.2%, 5.1%)	0.91 (0.67, 1.24)	26.2 (16.4, 37.1)	20.27 (2.74, 149.86) [p=0.0032]
All Above (↑)	ELF: 10.50 VCTE: 20.0 kPa FIB-4: 2.67	266	32	12.0% (8.4%, 16.6%)	3.50 (2.70, 4.53)	100.3 (65.8, 137.9)	77.34 (10.56, 566.21) [p<0.0001]
All Below (↓)	ELF: 11.30 VCTE: 30.0 kPa FIB-4: 3.48	988	7	0.7% (0.3%, 1.5%)	0.18 (0.09, 0.37)	5.2 (1.5, 9.6)	1.00 (Reference)
Disagree	ELF: 11.30 VCTE: 30.0 kPa FIB-4: 3.48	443	28	6.3% (4.2%, 9.0%)	1.73 (1.31, 2.28)	49.6 (31.9, 69.1)	9.72 (4.24, 22.26) [p<0.0001]
All Above (↑)	ELF: 11.30 VCTE: 30.0 kPa FIB-4: 3.48	84	22	26.2% (17.2%, 36.9%)	9.08 (6.03, 13.65)	243.8 (144.1, 354.7)	48.16 (20.55, 112.87) [p<0.0001]

[a] All Below: all 3 NITs < respective cutoffs; Disagree: 1 or 2 NITs < respective cutoffs; All Above: all 3 NITs ≥ respective cutoffs

Fig. S2.

Heatmaps of observed liver-related event risk by baseline ELF and VCTE category

A

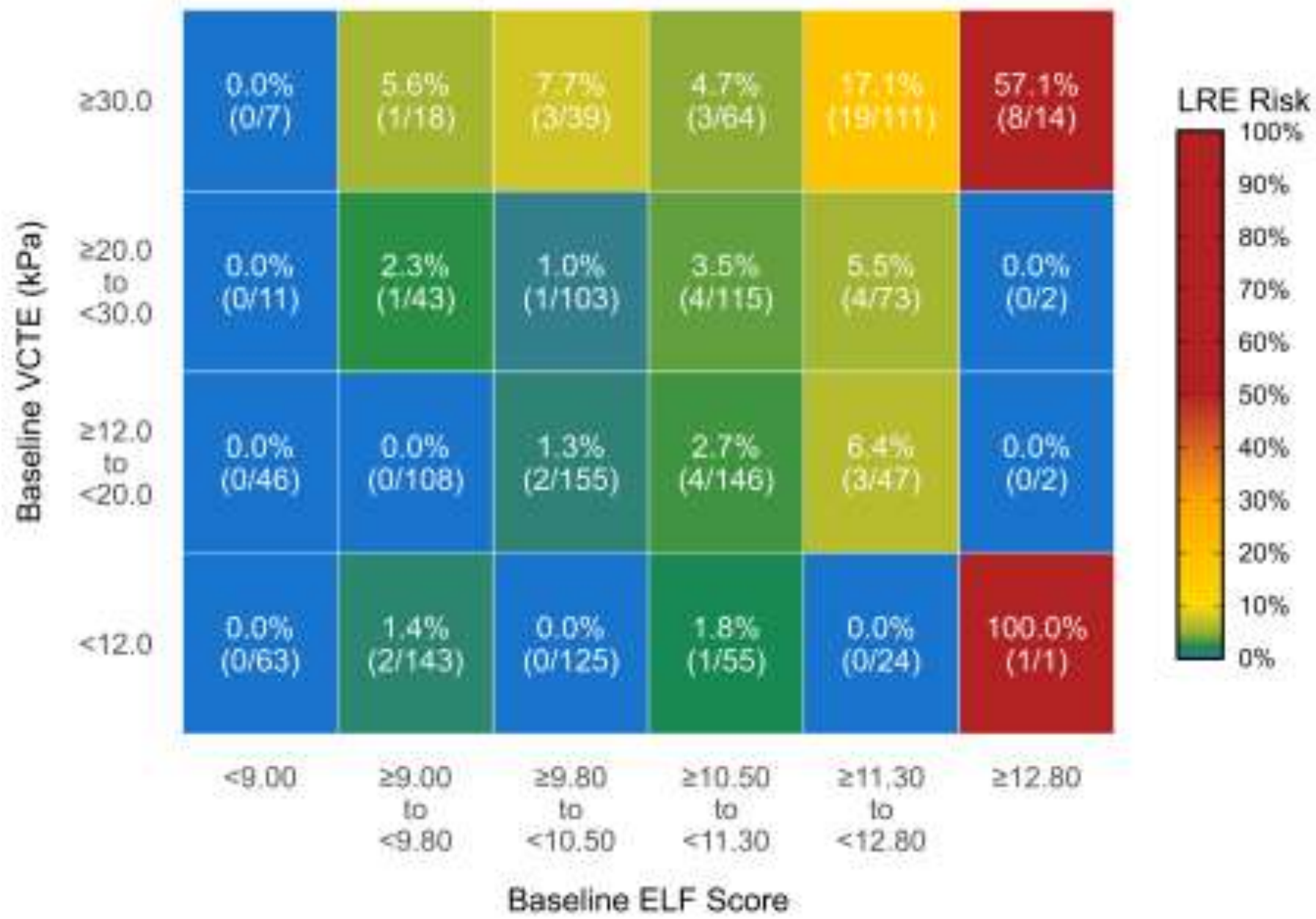

B

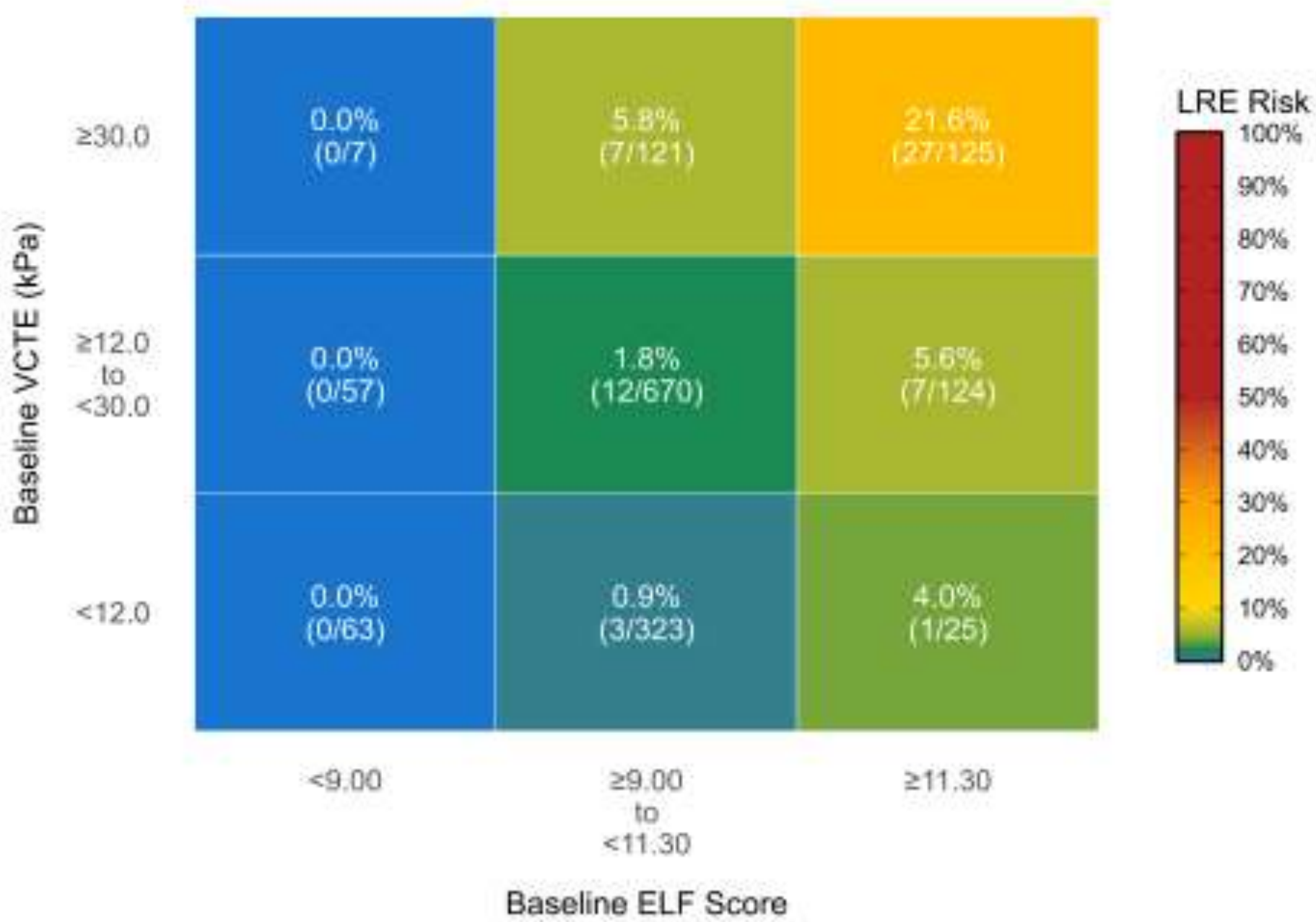

Cells display the proportion of patients experiencing a liver-related event within each ELF/VCTE stratum, with denominators shown in parentheses. Colour intensity reflects increasing event risk, but does not imply statistical significance between strata. (A) Full stratification of baseline ELF and VCTE categories. (B) Reduced stratification combining adjacent strata with similar event rates and limited sample sizes for display purposes.

Fig. S3.
Kaplan-Meier curves for event-free probability of LREs (stratification by baseline NASH CRN stage)

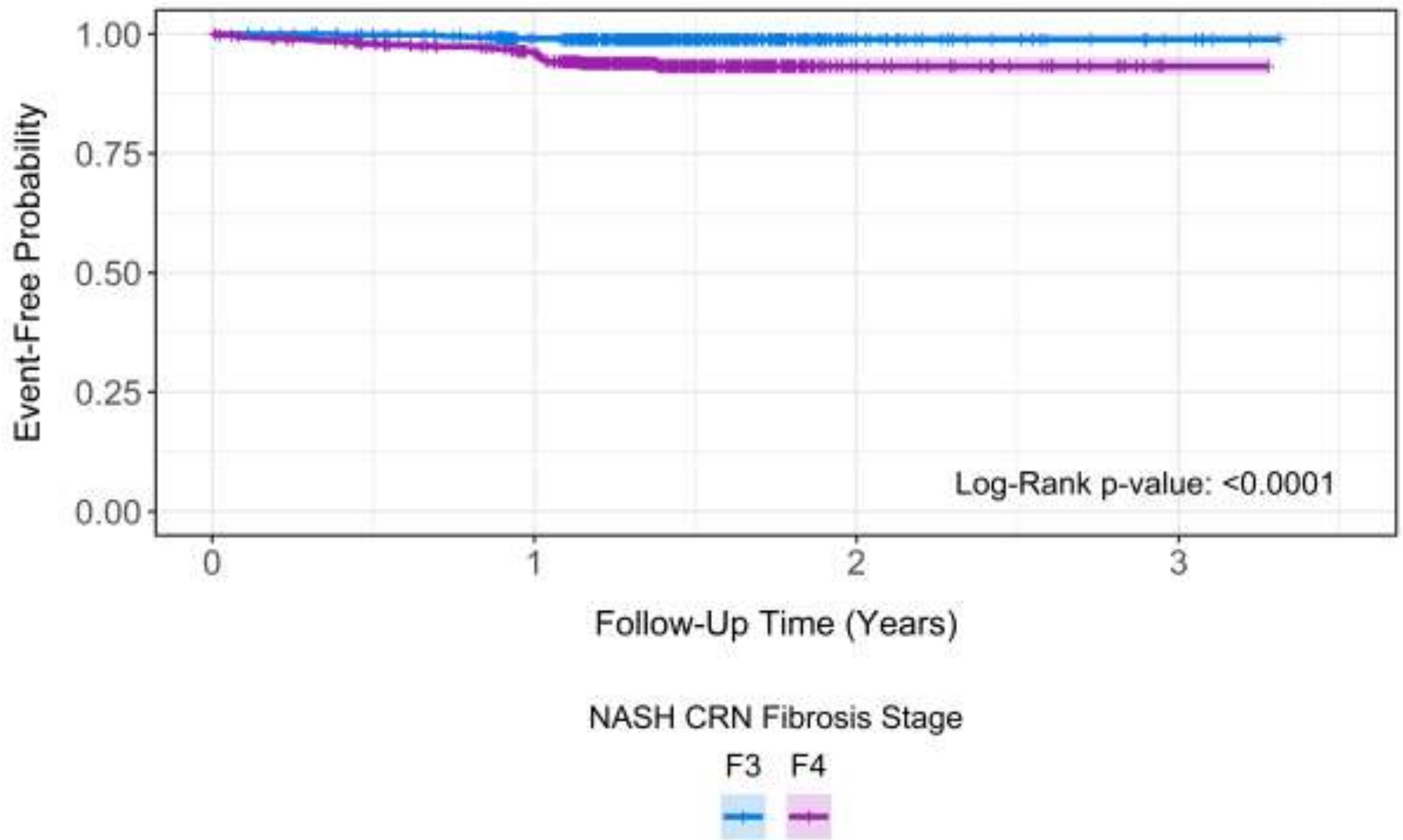

At Risk (Events)

F3	660 (0)	537 (6)	32 (7)	6 (7)
F4	853 (0)	689 (33)	29 (50)	1 (50)

Fig. S4.
Kaplan-Meier curves for event-free probability of LREs based on alignment of ELF, VCTE and FIB-4

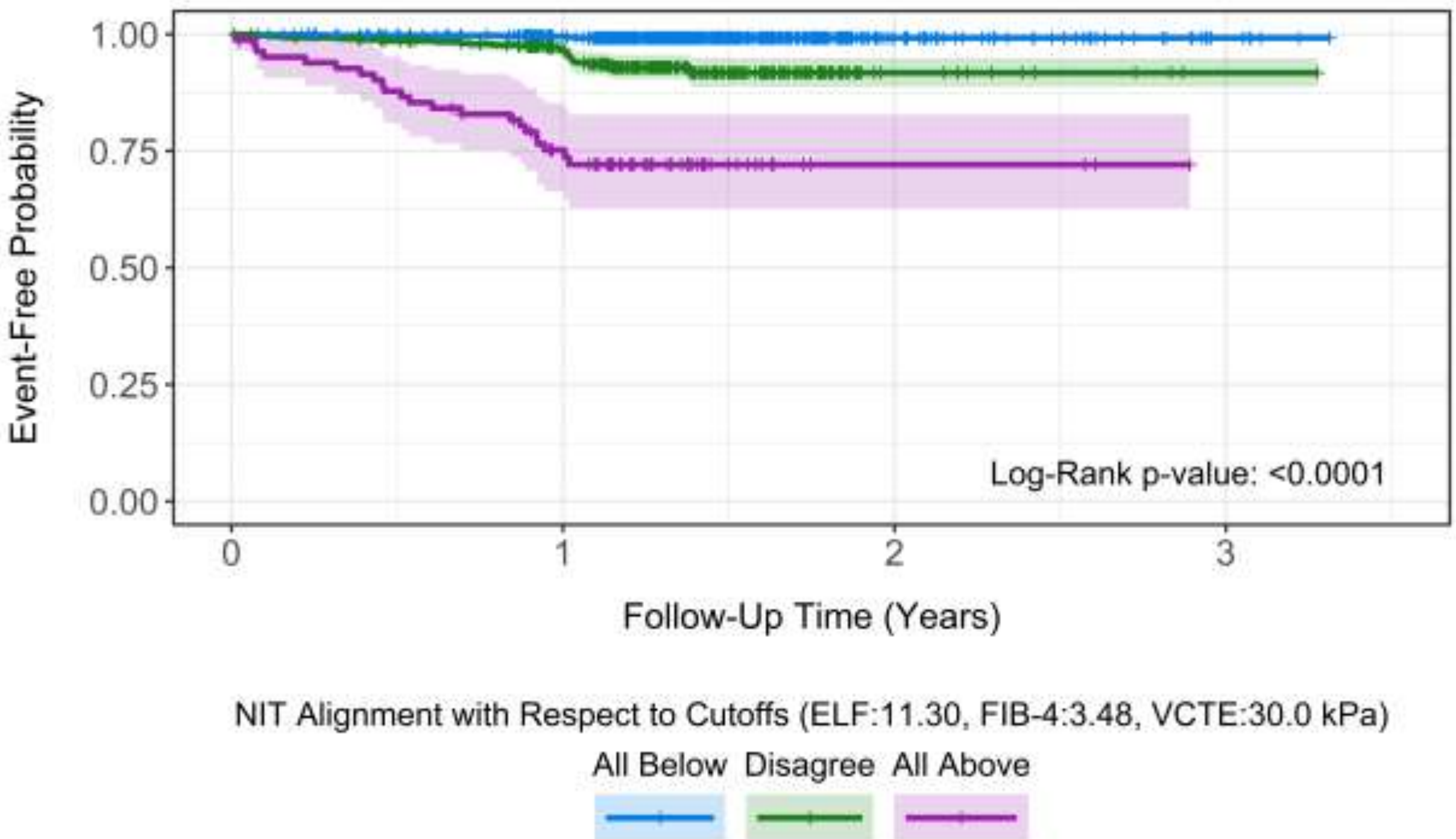

At Risk (Events)

All Below	988 (0)	858 (5)	48 (7)	6 (7)
Disagree	443 (0)	321 (14)	11 (28)	1 (28)
All Above	84 (0)	48 (20)	3 (22)	0 (22)

Risk stratification based on NIT alignment with respect to cutoffs (ELF:11.30, VCTE:30.0 kPa, FIB-4: 3.48). “All Below” (Blue): ELF <11.30 & VCTE <30.0 kPa & FIB-4 <3.48 ; “Disagree” (Green): ELF <11.30 & VCTE <30.0 kPa & FIB-4 ≥3.48 OR ELF <11.30 & VCTE ≥30.0 kPa & FIB-4 <3.48 OR ELF ≥11.30 & VCTE <30.0 kPa & FIB-4 <3.48 OR ELF ≥11.30 & VCTE ≥30.0 kPa & FIB-4 <3.48 OR ELF ≥11.30 & VCTE <30.0 kPa & FIB-4 ≥3.48 OR ELF <11.30 & VCTE ≥30.0 kPa & FIB-4 ≥3.48; “All Agree” (Purple): ELF ≥11.30 & VCTE ≥30.0 kPa & FIB-4 ≥3.48

5. Model Comparisons

Table S11. Summary statistics from Cox regressions for risk-stratification models of ELF and VCTE alone and in combination

Model	Low Stratum	Mid Stratum	High Stratum	C-Index	logLik	AIC	BIC
VCTE Alone 12.0 / 20.0 kPa	<12.0 kPa	≥12.0 to <20.0 kPa	≥20.0 kPa	0.708	-390.88	785.75	789.84
ELF Alone 9.80 / 11.30	<9.80	≥9.80 to <11.30	≥11.30	0.758	-379.01	762.03	766.12
ELF/VCTE Combo 1 10.50 / 15.0 kPa	ELF <10.50 & VCTE <15.0 kPa	Disagree[a]	ELF ≥10.50 & VCTE ≥15.0 kPa	0.750	-383.67	771.34	775.43
ELF/VCTE Combo 2 10.50 / 20.0 kPa	ELF <10.50 & VCTE <20.0 kPa	Disagree	ELF ≥10.50 & VCTE ≥20.0 kPa	0.770	-380.35	764.70	768.78
ELF/VCTE Combo 3 11.30 / 20.0 kPa	ELF <11.30 & VCTE <20.0 kPa	Disagree	ELF ≥11.30 & VCTE ≥20.0 kPa	0.781	-374.25	752.5	756.59
ELF/VCTE Combo 4 11.30 / 25.0 kPa	ELF <11.30 & VCTE <25.0 kPa	Disagree	ELF ≥11.30 & VCTE ≥25.0 kPa	0.783	-370.84	745.68	749.77
ELF/VCTE Combo 5 11.30 / 30.0 kPa	ELF <11.30 & VCTE <30.0 kPa	Disagree	ELF ≥11.30 & VCTE ≥30.0 kPa	0.786	-368.21	740.42	744.51

[a] "Disagree" for the combination models refers to one NIT < cutoff and the other NIT ≥ cutoff

Fig. S5.
Time-dependent area under the receiver operator characteristic curve by baseline ELF, VCTE or ELF/VCTE combination

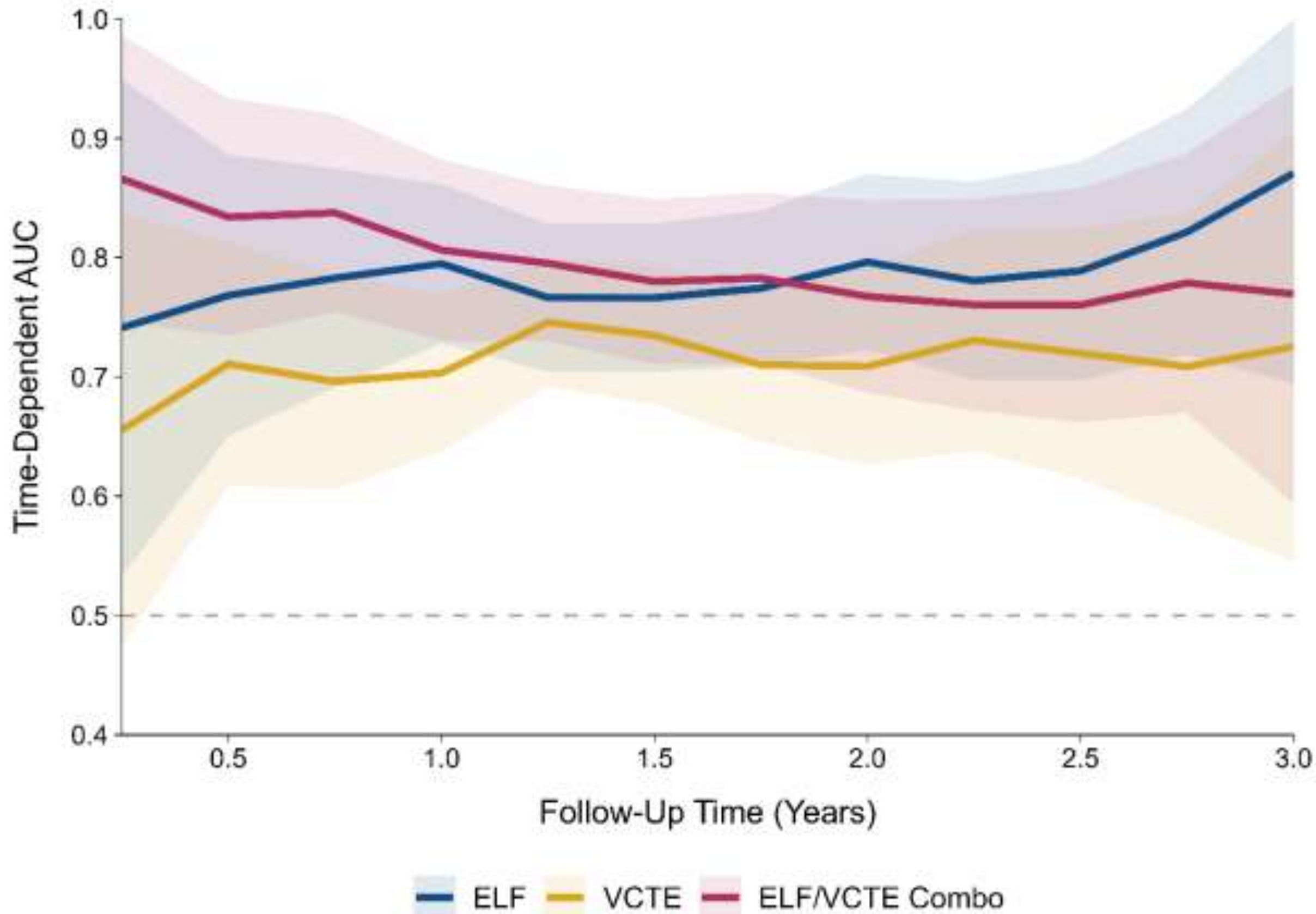

ELF (stratified by cutoffs at 9.80 and 11.30), VCTE (stratified by cutoffs at 12.0 and 20.0 kPa), or ELF and VCTE combination (agreement-based stratification using 11.30 and 30.0 kPa cutoffs).

Table S12.
Time-dependent area under the receiver operator characteristic curve (AUC(t)) by baseline ELF and VCTE alone and combination

Time (Years)	AUC(t) (95% CI) for Each Model			P-Value for Comparisons		
	ELF	VCTE	ELF/VCTE Combination	ELF vs VCTE	ELF/VCTE vs ELF	ELF/VCTE vs VCTE
0.25	0.741 (0.533, 0.950)	0.656 (0.474, 0.837)	0.866 (0.746, 0.986)	0.4508	0.0767	0.0004
0.50	0.768 (0.650, 0.886)	0.711 (0.609, 0.813)	0.834 (0.735, 0.933)	0.3561	0.1364	0.0099
0.75	0.783 (0.691, 0.875)	0.696 (0.606, 0.787)	0.838 (0.755, 0.921)	0.0930	0.1170	0.0001
1.00	0.795 (0.728, 0.862)	0.703 (0.637, 0.770)	0.806 (0.730, 0.882)	0.0097	0.6613	0.0010
1.25	0.766 (0.704, 0.829)	0.746 (0.692, 0.800)	0.795 (0.730, 0.861)	0.5294	0.2209	0.1057
1.50	0.766 (0.704, 0.829)	0.735 (0.677, 0.793)	0.780 (0.711, 0.849)	0.3648	0.5748	0.1589
1.75	0.774 (0.709, 0.840)	0.710 (0.645, 0.775)	0.783 (0.711, 0.854)	0.0888	0.7403	0.0354
2.00	0.796 (0.722, 0.870)	0.709 (0.626, 0.792)	0.767 (0.687, 0.848)	0.0443	0.2807	0.1462
2.25	0.781 (0.697, 0.864)	0.731 (0.638, 0.823)	0.760 (0.672, 0.849)	0.2969	0.4675	0.4840
2.50	0.789 (0.697, 0.881)	0.720 (0.615, 0.825)	0.760 (0.662, 0.858)	0.1870	0.3179	0.3982
2.75	0.821 (0.718, 0.925)	0.709 (0.580, 0.837)	0.779 (0.670, 0.888)	0.0801	0.1798	0.2436
3.00	0.871 (0.695, 1.000)	0.725 (0.545, 0.905)	0.769 (0.594, 0.945)	0.0729	0.0001	0.5767

<u>ELF Strata</u>
Low: ELF <9.80
Mid: ELF ≥9.80 to <11.30
High: ELF ≥11.30

<u>VCTE Strata</u>
Low: VCTE <12.0 kPa
Mid: VCTE ≥12.0 to <20.0 kPa
High: VCTE ≥20.0 kPa

<u>ELF + VCTE Combination Strata</u>
Agree ↓: ELF <11.30 & VCTE <30.0 kPa
Disagree: ELF ≥11.30 & VCTE <30.0 kPa; OR ELF <11.30 & VCTE ≥30.0 kPa
Agree ↑: ELF ≥11.30 & VCTE ≥30.0 kPa

6. Sensitivity Analyses

Table S13.
Baseline ELF/VCTE combination to predict liver-related events (sensitivity analysis: treatment vs placebo)

Baseline ELF/VCTE Strata	Study Arm	n	Events	Event Rate (95% CI)	P-value[a]
Agree Above (↑): ELF ≥11.30 & VCTE ≥30.0 kPa	Treatment	106	24	22.6% (15.1%, 31.8%)	0.7625
	Placebo	19	3	15.8% (3.4%, 39.6%)	
	Pooled	125	27	21.6% (14.7%, 29.8%)	---
Disagree: ELF ≥11.30 & VCTE <30.0 kPa OR ELF <11.30 & VCTE ≥30.0 kPa	Treatment	219	10	4.6% (2.2%, 8.2%)	0.3530
	Placebo	58	5	8.6% (2.9%, 19.0%)	
	Pooled	277	15	5.4% (3.1%, 8.8%)	---
Agree Below (↓): ELF <11.30 & VCTE <30.0 kPa	Treatment	866	9	1.0% (0.5%, 2.0%)	0.1148
	Placebo	247	6	2.4% (0.9%, 5.2%)	
	Pooled	1113	15	1.3% (0.8%, 2.2%)	---

[a] P-values determined by Fisher's exact 2-sided test.

Table S14.
Baseline ELF/VCTE combination to predict liver-related events (sensitivity analysis: diabetes status)

Baseline ELF/VCTE Strata	Diabetes	n	Events	Event Rate (95% CI)	P-value[a]
Agree Above (↑): ELF ≥11.30 & VCTE ≥30.0 kPa	Present	95	17	17.9% (10.8%, 27.1%)	0.0812
	Absent	30	10	33.3% (17.3%, 52.8%)	
	Pooled	125	27	21.6% (14.7%, 29.8%)	---
Disagree: ELF ≥11.30 & VCTE <30.0 kPa OR ELF <11.30 & VCTE ≥30.0 kPa	Present	191	9	4.7% (2.2%, 8.8%)	0.5664
	Absent	86	6	7.0% (2.6%, 14.6%)	
	Pooled	277	15	5.4% (3.1%, 8.8%)	---
Agree Below (↓): ELF <11.30 & VCTE <30.0 kPa	Present	811	12	1.5% (0.8%, 2.6%)	0.7710
	Absent	302	3	1.0% (0.2%, 2.9%)	
	Pooled	1113	15	1.3% (0.8%, 2.2%)	---

[a] P-values determined by Fisher's exact 2-sided test.

Table S15.
Baseline ELF/VCTE combination to predict liver-related events (sensitivity analysis: age)

Baseline ELF/VCTE Strata	Age	n	Events	Event Rate (95% CI)	P-value[a]
Agree Above (↑): ELF ≥11.30 & VCTE ≥30.0 kPa	<60	61	9	14.8% (7.0%, 26.2%)	0.0839
	≥60	64	18	28.1% (17.6%, 40.8%)	
	Pooled	125	27	21.6% (14.7%, 29.8%)	---
Disagree: ELF ≥11.30 & VCTE <30.0 kPa OR ELF <11.30 & VCTE ≥30.0 kPa	<60	144	6	4.2% (1.5%, 8.8%)	0.4287
	≥60	133	9	6.8% (3.1%, 12.5%)	
	Pooled	277	15	5.4% (3.1%, 8.8%)	---
Agree Below (↓): ELF <11.30 & VCTE <30.0 kPa	<60	608	6	1.0% (0.4%, 2.1%)	0.3012
	≥60	505	9	1.8% (0.8%, 3.4%)	
	Pooled	1113	15	1.3% (0.8%, 2.2%)	---

[a] P-values determined by Fisher's exact 2-sided test.

7. Diagnostic Analyses

Comparability of diagnostic performance of the prognostically-matched NIT cutoffs was evaluated. Data were pooled from 1443 participants in the following 6 clinical trials: STELLAR-3, STELLAR-4, GS-US-321-0105, GS-US-321-0106, ATLAS, and NASH-CX (Fig. S6). Samples were included in the analysis which had complete measurements at 1-year for NASH CRN fibrosis stage, VCTE, and laboratory tests including ELF (N = 1443). Additionally, an expanded dataset with complete cases for ELF and NASH CRN stage (N = 2390) was also used for ELF-only analyses.

The ELF cutoffs used in this comparison were: 8.30, 9.00, 9.80, 10.50, 11.30, and 12.00. The cutoffs for VCTE were 5.0, 10.0, 12.0, 20.0, 30.0, and 50.0 kPa. These thresholds were used individually and in combination as a prognostically-aligned ELF/VCTE pair.

Analyses for the discrimination of significant fibrosis (≥F2), advanced fibrosis (≥F3) and cirrhosis (F4) were evaluated using samples with histological stage (NASH CRN), ELF, VCTE, and FIB-4 measurements. Measurements of histology and NITs were based on the 1-year time point of the respective trials due to limited <F2 samples at baseline. Calculations were performed for each NIT alone and in combination with one another. To assess correlation to histology, sensitivity, specificity, diagnostic accuracy, and associated Clopper-Pearson exact 95% confidence intervals were calculated.

Summaries of the baseline demographics and clinical characteristics in the diagnostic datasets are presented in Table S16.

Results for are presented in Table S17 (≥F2 vs <F2), Table S18 (≥F3 vs <F3), Table S19 (F4 vs <F4), and Table S20 (expanded dataset for ELF).

Fig. S6. Exclusions from primary diagnostic analyses

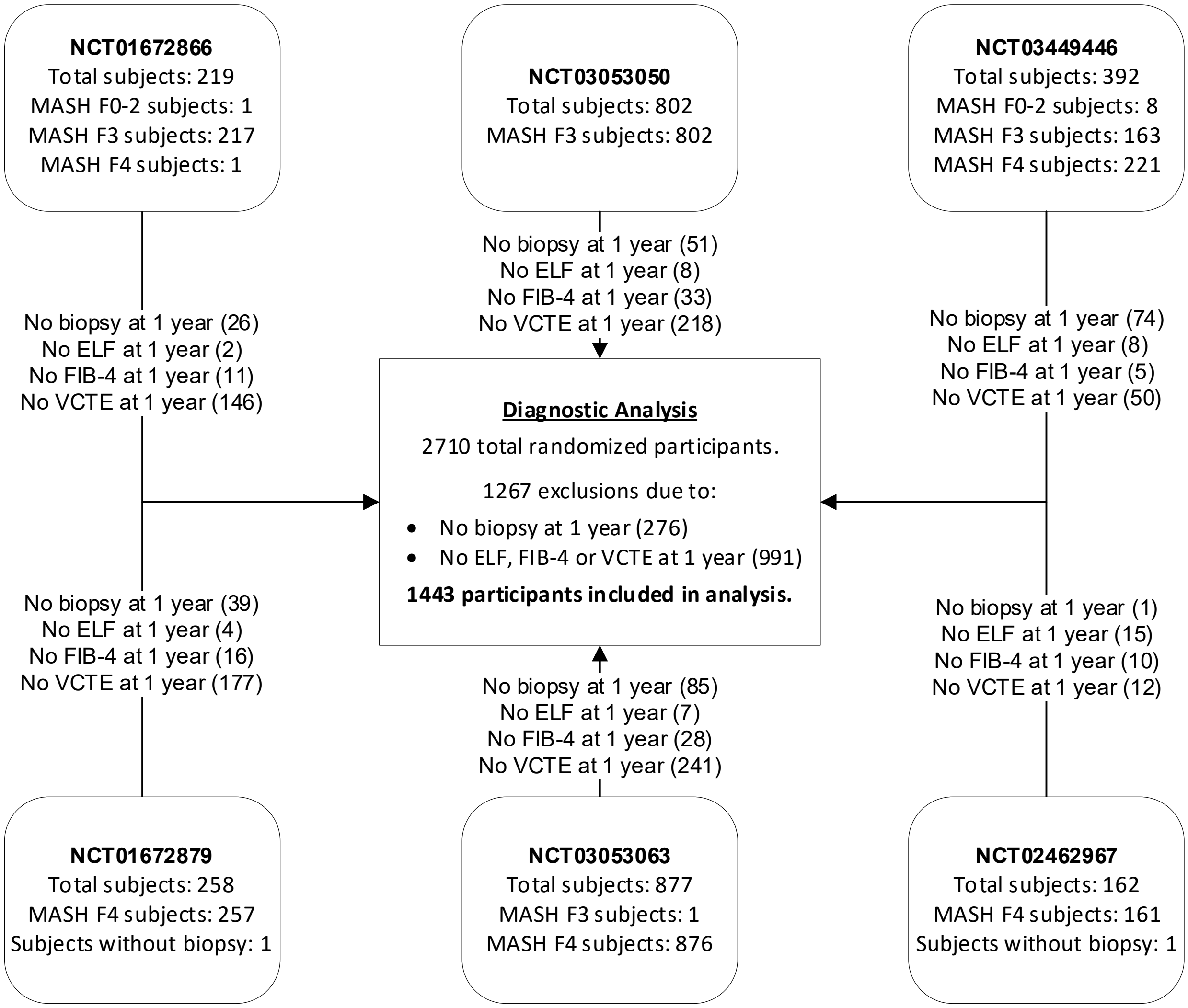

Table S16. Baseline demographics and clinical characteristics of diagnostic datasets

Baseline Parameter	Primary Diagnostic Dataset: Complete Cases for ELF, VCTE, FIB-4, NASH CRN Stage at 1 Year	Expanded Dataset: Complete Cases for ELF and NASH CRN Stage at 1 Year
N	1443	2390
Female (%)	883 (61%)	883 (61%)
Type 2 diabetes present (%)	1047 (73%)	1717 (72%)
Age category (%)		
≤34	18 (1%)	39 (2%)
35-39	31 (2%)	50 (2%)
40-44	64 (4%)	93 (4%)
45-49	133 (9%)	227 (9%)
50-54	190 (13%)	322 (13%)
55-59	312 (22%)	550 (23%)
60-64	316 (22%)	550 (23%)
≥65	379 (26%)	559 (23%)
NASH CRN stage (%)[a]		
F0	4 (0%)	6 (0%)
F1	21 (1%)	38 (1%)
F2	73 (5%)	123 (5%)
F3	586 (41%)	945 (41%)
F4	759 (53%)	1278 (53%)
Median ELF score (IQR)[a]	10.38 (9.70, 11.10)	10.40 (9.69, 11.16)
Median FIB-4 score (IQR)[a]	2.09 (1.40, 3.16)	2.11 (1.40, 3.26)
Median VCTE in kPa (IQR)[a]	15.3 (10.6, 21.8)	15.4 (10.7, 21.8)

[a] Parameters for the diagnostic dataset were obtained at the 1-year timepoint.

Table S17. ELF, VCTE and combinations vs NASH CRN stage at 1-year timepoint (≥F2 vs <F2)

Analysis	ELF Cutoff	VCTE Cutoff (kPa)	TP	FP	FN	TN	Sensitivity (95% CI)	Specificity (95% CI)	Total Agreement (95% CI)
ELF Only	8.30	---	1394	23	24	2	98.3% (97.5%, 98.9%)	8.0% (1.0%, 26.0%)	96.7% (95.7%, 97.6%)
	9.00	---	1303	14	115	11	91.9% (90.3%, 93.3%)	44.0% (24.4%, 65.1%)	91.1% (89.5%, 92.5%)
	9.80	---	1035	6	383	19	73.0% (70.6%, 75.3%)	76.0% (54.9%, 90.6%)	73.0% (70.7%, 75.3%)
	10.50	---	664	1	754	24	46.8% (44.2%, 49.5%)	96.0% (79.6%, 99.9%)	47.7% (45.1%, 50.3%)
	11.30	---	294	0	1124	25	20.7% (18.7%, 22.9%)	100.0% (86.3%, 100.0%)	22.1% (20.0%, 24.3%)
	12.00	---	102	0	1316	25	7.2% (5.9%, 8.7%)	100.0% (86.3%, 100.0%)	8.8% (7.4%, 10.4%)
VCTE Only	---	5.0	1403	21	15	4	98.9% (98.3%, 99.4%)	16.0% (4.5%, 36.1%)	97.5% (96.6%, 98.2%)
	---	8.0[a]	1284	10	134	15	90.6% (88.9%, 92.0%)	60.0% (38.7%, 78.9%)	90.0% (88.4%, 91.5%)
	---	10.0[a]	1139	5	279	20	80.3% (78.2%, 82.4%)	80.0% (59.3%, 93.2%)	80.3% (78.2%, 82.3%)
	---	12.0	969	3	449	22	68.3% (65.8%, 70.8%)	88.0% (68.8%, 97.5%)	68.7% (66.2%, 71.1%)
	---	20.0	474	1	944	24	33.4% (31.0%, 36.0%)	96.0% (79.6%, 99.9%)	34.5% (32.1%, 37.0%)
	---	30.0	192	1	1226	24	13.5% (11.8%, 15.4%)	96.0% (79.6%, 99.9%)	15.0% (13.2%, 16.9%)
	---	50.0	47	0	1371	25	3.3% (2.4%, 4.4%)	100.0% (86.3%, 100.0%)	5.0% (3.9%, 6.2%)
ELF/VCTE Combo	8.30	5.0	1381	20	37	5	97.4% (96.4%, 98.2%)	20.0% (6.8%, 40.7%)	96.0% (94.9%, 97.0%)
	9.00	8.0[a]	1206	5	212	20	85.0% (83.1%, 86.9%)	80.0% (59.3%, 93.2%)	85.0% (83.0%, 86.8%)
	9.00	10.0[a]	1085	1	333	24	76.5% (74.2%, 78.7%)	96.0% (79.6%, 99.9%)	76.9% (74.6%, 79.0%)
	9.80	12.0	806	1	612	24	56.8% (54.2%, 59.4%)	96.0% (79.6%, 99.9%)	57.5% (54.9%, 60.1%)
	10.50	20.0	349	0	1069	25	24.6% (22.4%, 26.9%)	100.0% (86.3%, 100.0%)	25.9% (23.7%, 28.3%)
	11.30	30.0	105	0	1313	25	7.4% (6.1%, 8.9%)	100.0% (86.3%, 100.0%)	9.0% (7.6%, 10.6%)
	12.00	50.0	20	0	1398	25	1.4% (0.9%, 2.2%)	100.0% (86.3%, 100.0%)	3.1% (2.3%, 4.2%)

TP = True Positive, FP = False Positive, FN = False Negative, TN = True Negative

[a] VCTE cutoffs of 8.0 and 10.0 kPa both have prognostic alignment to ELF 9.00.

Table S18. ELF, VCTE and combinations vs NASH CRN stage at 1-year timepoint (≥F3 vs <F3)

Analysis	ELF Cutoff	VCTE Cutoff (kPa)	TP	FP	FN	TN	Sensitivity (95% CI)	Specificity (95% CI)	Total Agreement (95% CI)
ELF Only	8.30	---	1329	88	16	10	98.8% (98.1%, 99.3%)	10.2% (5.0%, 18.0%)	92.8% (91.3%, 94.1%)
	9.00	---	1253	64	92	34	93.2% (91.7%, 94.5%)	34.7% (25.4%, 45.0%)	89.2% (87.5%, 90.7%)
	9.80	---	1012	29	333	69	75.2% (72.8%, 77.5%)	70.4% (60.3%, 79.2%)	74.9% (72.6%, 77.1%)
	10.50	---	655	10	690	88	48.7% (46.0%, 51.4%)	89.8% (82.0%, 95.0%)	51.5% (48.9%, 54.1%)
	11.30	---	294	0	1051	98	21.9% (19.7%, 24.2%)	100.0% (96.3%, 100.0%)	27.2% (24.9%, 29.5%)
	12.00	---	102	0	1243	98	7.6% (6.2%, 9.1%)	100.0% (96.3%, 100.0%)	13.9% (12.1%, 15.8%)
VCTE Only	---	5.0	1331	93	14	5	99.0% (98.3%, 99.4%)	5.1% (1.7%, 11.5%)	92.6% (91.1%, 93.9%)
	---	8.0[a]	1233	61	112	37	91.7% (90.1%, 93.1%)	37.8% (28.2%, 48.1%)	88.0% (86.2%, 89.6%)
	---	10.0[a]	1102	42	243	56	81.9% (79.8%, 84.0%)	57.1% (46.7%, 67.1%)	80.2% (78.1%, 82.3%)
	---	12.0	950	22	395	76	70.6% (68.1%, 73.1%)	77.6% (68.0%, 85.4%)	71.1% (68.7%, 73.4%)
	---	20.0	471	4	874	94	35.0% (32.5%, 37.6%)	95.9% (89.9%, 98.9%)	39.2% (36.6%, 41.7%)
	---	30.0	190	3	1155	95	14.1% (12.3%, 16.1%)	96.9% (91.3%, 99.4%)	19.8% (17.7%, 21.9%)
	---	50.0	47	0	1298	98	3.5% (2.6%, 4.6%)	100.0% (96.3%, 100.0%)	10.0% (8.5%, 11.7%)
ELF/VCTE Combo	8.30	5.0	1317	84	28	14	97.9% (97.0%, 98.6%)	14.3% (8.0%, 22.8%)	92.2% (90.7%, 93.6%)
	9.00	8.0[a]	1168	43	177	55	86.8% (84.9%, 88.6%)	56.1% (45.7%, 66.1%)	84.8% (82.8%, 86.6%)
	9.00	10.0[a]	1057	29	288	69	78.6% (76.3%, 80.8%)	70.4% (60.3%, 79.2%)	78.0% (75.8%, 80.1%)
	9.80	12.0	800	7	545	91	59.5% (56.8%, 62.1%)	92.9% (85.8%, 97.1%)	61.7% (59.2%, 64.3%)
	10.50	20.0	349	0	996	98	25.9% (23.6%, 28.4%)	100.0% (96.3%, 100.0%)	31.0% (28.6%, 33.4%)
	11.30	30.0	105	0	1240	98	7.8% (6.4%, 9.4%)	100.0% (96.3%, 100.0%)	14.1% (12.3%, 16.0%)
	12.00	50.0	20	0	1325	98	1.5% (0.9%, 2.3%)	100.0% (96.3%, 100.0%)	8.2% (6.8%, 9.7%)

TP = True Positive, FP = False Positive, FN = False Negative, TN = True Negative

[a] VCTE cutoffs of 8.0 and 10.0 kPa both have prognostic alignment to ELF 9.00.

Table S19. ELF, VCTE and combinations vs NASH CRN stage at 1-year timepoint (F4 vs <F4)

Analysis	ELF Cutoff	VCTE Cutoff (kPa)	TP	FP	FN	TN	Sensitivity (95% CI)	Specificity (95% CI)	Total Agreement (95% CI)
ELF Only	8.30	---	749	668	10	16	98.7% (97.6%, 99.4%)	2.3% (1.3%, 3.8%)	53.0% (50.4%, 55.6%)
	9.00	---	732	585	27	99	96.4% (94.9%, 97.6%)	14.5% (11.9%, 17.3%)	57.6% (55.0%, 60.2%)
	9.80	---	646	395	113	289	85.1% (82.4%, 87.6%)	42.3% (38.5%, 46.1%)	64.8% (62.3%, 67.3%)
	10.50	---	464	201	295	483	61.1% (57.6%, 64.6%)	70.6% (67.0%, 74.0%)	65.6% (63.1%, 68.1%)
	11.30	---	238	56	521	628	31.4% (28.1%, 34.8%)	91.8% (89.5%, 93.8%)	60.0% (57.4%, 62.6%)
	12.00	---	90	12	669	672	11.9% (9.6%, 14.4%)	98.2% (97.0%, 99.1%)	52.8% (50.2%, 55.4%)
VCTE Only	---	5.0	757	667	2	17	99.7% (99.1%, 100.0%)	2.5% (1.5%, 3.9%)	53.6% (51.0%, 56.2%)
	---	8.0[a]	746	548	13	136	98.3% (97.1%, 99.1%)	19.9% (17.0%, 23.1%)	61.1% (58.6%, 63.6%)
	---	10.0[a]	712	432	47	252	93.8% (91.9%, 95.4%)	36.8% (33.2%, 40.6%)	66.8% (64.3%, 69.2%)
	---	12.0	651	321	108	363	85.8% (83.1%, 88.2%)	53.1% (49.3%, 56.9%)	70.3% (67.8%, 72.6%)
	---	20.0	396	79	363	605	52.2% (48.6%, 55.8%)	88.5% (85.8%, 90.7%)	69.4% (66.9%, 71.7%)
	---	30.0	172	21	587	663	22.7% (19.7%, 25.8%)	96.9% (95.3%, 98.1%)	57.9% (55.3%, 60.4%)
	---	50.0	46	1	713	683	6.1% (4.5%, 8.0%)	99.9% (99.2%, 100.0%)	50.5% (47.9%, 53.1%)
ELF/VCTE Combo	8.30	5.0	747	654	12	30	98.4% (97.3%, 99.2%)	4.4% (3.0%, 6.2%)	53.8% (51.2%, 56.4%)
	9.00	8.0[a]	722	489	37	195	95.1% (93.3%, 96.5%)	28.5% (25.2%, 32.1%)	63.5% (61.0%, 66.0%)
	9.00	10.0[a]	693	393	66	291	91.3% (89.1%, 93.2%)	42.5% (38.8%, 46.3%)	68.2% (65.7%, 70.6%)
	9.80	12.0	575	232	184	452	75.8% (72.5%, 78.8%)	66.1% (62.4%, 69.6%)	71.2% (68.8%, 73.5%)
	10.50	20.0	301	48	458	636	39.7% (36.2%, 43.2%)	93.0% (90.8%, 94.8%)	64.9% (62.4%, 67.4%)
	11.30	30.0	99	6	660	678	13.0% (10.7%, 15.6%)	99.1% (98.1%, 99.7%)	53.8% (51.2%, 56.4%)
	12.00	50.0	20	0	739	684	2.6% (1.6%, 4.0%)	100.0% (99.5%, 100.0%)	48.8% (46.2%, 51.4%)

TP = True Positive, FP = False Positive, FN = False Negative, TN = True Negative

[a] VCTE cutoffs of 8.0 and 10.0 kPa both have prognostic alignment to ELF 9.00.

Table S20. ELF vs NASH CRN stage at 1-year timepoint for fibrosis staging (expanded dataset)

Comparison	ELF Cutoff	TP	FP	FN	TN	Sensitivity (95% CI)	Specificity (95% CI)	Total Agreement (95% CI)
≥F2 vs <F2	8.30	2306	40	40	4	98.3% (97.7%, 98.8%)	9.1% (2.5%, 21.7%)	96.7% (95.9%, 97.3%)
	9.00	2149	27	197	17	91.6% (90.4%, 92.7%)	38.6% (24.4%, 54.5%)	90.6% (89.4%, 91.8%)
	9.80	1703	11	643	33	72.6 (70.7%, 74.4%)	75.0% (59.7%, 86.8%)	72.6% (70.8%, 74.4%)
	10.50	1121	4	1225	40	47.8% (45.7%, 49.8%)	90.9% (78.3%, 97.5%)	48.6% (46.6%, 50.6%)
	11.30	510	1	1836	43	21.7% (20.1%, 23.5%)	97.7% (88.0%, 99.9%)	23.1% (21.5%, 24.9%)
	12.00	200	1	2146	43	8.5% (7.4%, 9.7%)	97.7% (88.0%, 99.9%)	10.2% (9.0%, 11.4%)
≥F3 vs <F3	8.30	2191	155	32	12	98.6% (98.0%, 99.0%)	7.2% (3.8%, 12.2%)	92.2% (91.0%, 93.2%)
	9.00	2061	115	162	52	92.7% (91.6%, 93.8%)	31.1% (24.2%, 38.8%)	88.4% (87.1%, 89.7%)
	9.80	1667	47	556	120	75.0% (73.1%, 76.8%)	71.9% (64.4%, 78.5%)	74.8% (73.0%, 76.5%)
	10.50	1105	20	1118	147	49.7% (47.6%, 51.8%)	88.0% (82.1%, 92.5%)	52.4% (50.4%, 54.4%)
	11.30	508	3	1715	164	22.9% (21.1%, 24.7%)	98.2% (94.8%, 99.6%)	28.1% (26.3%, 30.0%)
	12.00	199	2	2024	165	9.0% (7.8%, 10.2%)	98.8% (95.7%, 99.9%)	15.2% (13.8%, 16.7%)
F4 vs <F4	8.30	1263	1083	15	29	98.8% (98.1%, 99.3%)	2.6% (1.8%, 3.7%)	54.1% (52.0%, 56.1%)
	9.00	1231	945	47	167	96.3% (95.1%, 97.3%)	15.0% (13.0%, 17.3%)	58.5% (56.5%, 60.5%)
	9.80	1088	626	190	486	85.1% (83.1%, 87.0%)	43.7% (40.8%, 46.7%)	65.9% (63.9%, 67.8%)
	10.50	809	316	469	796	63.3% (60.6%, 66.0%)	71.6% (68.8%, 74.2%)	67.2% (65.2%, 69.0%)
	11.30	424	87	854	1025	33.2% (30.6%, 35.8%)	92.2% (90.4%, 93.7%)	60.6% (58.6%, 62.6%)
	12.00	180	21	1098	1091	14.1% (12.2%, 16.1%)	98.1% (97.1%, 98.8%)	53.2% (51.2%, 55.2%)

TP = True Positive, FP = False Positive, FN = False Negative, TN = True Negative

8. TRIPOD Statement Checklist

Section/Topic	Item	Development (D) or Validation (V)?	Checklist Item	Section in Manuscript
**Title and Abstract**				
Title	1	D;V	Identify the study as developing and/or validating a multivariable prediction model, the target population, and the outcome to be predicted.	Title
Abstract	2	D;V	Provide a summary of objectives, study design, setting, participants, sample size, predictors, outcome, statistical analysis, results, and conclusions.	Abstract
**Introduction**				
Background and objectives	3a	D;V	Explain the medical context (including whether diagnostic or prognostic) and rationale for developing or validating the multivariable prediction model, including references to existing models.	Introduction
	3b	D;V	Specify the objectives, including whether the study describes the development or validation of the model, or both.	Introduction
**Methods**				
Source of data	4a	D;V	Describe the study design or source of data (e.g., randomized trial, cohort, or registry data), separately for the development and validation data sets, if applicable.	Methods<br>(Study population)
	4b	D;V	Specify the key study dates, including start of accrual; end of accrual; and, if applicable, end of follow-up.	Table S2
Participants	5a	D;V	Specify key elements of the study setting (e.g., primary care, secondary care, general population) including number and location of centres.	Table S2
	5b	D;V	Describe eligibility criteria for participants.	Table S2
	5c	D;V	Give details of treatments received, if relevant.	Table S2

Section/Topic	Item	Development (D) or Validation (V)?	Checklist Item	Section in Manuscript
Outcome	6a	D;V	Clearly define the outcome that is predicted by the prediction model, including how and when assessed.	Methods<br>(Analyses and endpoints)
	6b	D;V	Report any actions to blind assessment of the outcome to be predicted.	Methods<br>(Study assessments)
Predictors	7a	D;V	Clearly define all predictors used in developing the multivariable prediction model, including how and when they were measured.	Methods<br>(Analyses and endpoints)
	7b	D;V	Report any actions to blind assessment of predictors for the outcome and other predictors.	Methods<br>(Study assessments)
Sample size	8	D;V	Explain how the study size was arrived at.	Methods<br>(Study population)
Missing data	9	D;V	Describe how missing data were handled (e.g., complete-case analysis, single imputation, multiple imputation) with details of any imputation method.	Study inclusion required samples with results for ELF, VCTE and FIB-4 at baseline. There were no missing data for critical variables.
Statistical analysis methods	10a	D	Describe how predictors were handled in the analyses.	N/A
	10b	D	Specify type of model, all model-building procedures (including any predictor selection), and method for internal validation.	N/A
	10c	V	For validation, describe how the predictions were calculated.	Methods<br>(Analyses and endpoints)
	10d	D;V	Specify all measures used to assess model performance and, if relevant, to compare multiple models.	Methods<br>(Analyses and endpoints)
	10e	V	Describe any model updating (e.g., recalibration) arising from the validation, if done.	N/A

Section/Topic	Item	Development (D) or Validation (V)?	Checklist Item	Section in Manuscript
Risk groups	11	D;V	Provide details on how risk groups were created, if done.	Methods (Analyses and endpoints)
Development vs. validation	12	V	For validation, identify any differences from the development data in setting, eligibility criteria, outcome, and predictors.	Methods (Analyses and endpoints)
**Results**				
Participants	13a	D;V	Describe the flow of participants through the study, including the number of participants with and without the outcome and, if applicable, a summary of the follow-up time. A diagram may be helpful.	Fig. S1
	13b	D;V	Describe the characteristics of the participants (basic demographics, clinical features, available predictors), including the number of participants with missing data for predictors and outcome.	Table S2
	13c	V	For validation, show a comparison with the development data of the distribution of important variables (demographics, predictors and outcome).	Discussion
Model development	14a	D	Specify the number of participants and outcome events in each analysis.	N/A
	14b	D	If done, report the unadjusted association between each candidate predictor and outcome.	N/A
Model specification	15a	D	Present the full prediction model to allow predictions for individuals (i.e., all regression coefficients, and model intercept or baseline survival at a given time point).	N/A
	15b	D	Explain how to use the prediction model.	N/A
Model performance	16	D;V	Report performance measures (with CIs) for the prediction model.	Results

Section/Topic	Item	Development (D) or Validation (V)?	Checklist Item	Section in Manuscript
Model updating	17	V	If done, report the results from any model updating (i.e., model specification, model performance).	N/A
**Discussion**				
Limitations	18	D;V	Discuss any limitations of the study (such as nonrepresentative sample, few events per predictor, missing data).	Discussion
Interpretation	19a	V	For validation, discuss the results with reference to performance in the development data, and any other validation data.	Discussion
	19b	D;V	Give an overall interpretation of the results, considering objectives, limitations, results from similar studies, and other relevant evidence.	Discussion
Implications	20	D;V	Discuss the potential clinical use of the model and implications for future research.	Discussion
**Other information**				
Supplementary information	21	D;V	Provide information about the availability of supplementary resources, such as study protocol, Web calculator, and data sets.	References to Supplemental Tables and Figures listed throughout text
Funding	22	D;V	Give the source of funding and the role of the funders for the present study.	N/A

9. References

1. Loomba R, Huang DQ, Sanyal AJ, et al. Liver stiffness thresholds to predict disease progression and clinical outcomes in bridging fibrosis and cirrhosis. *Gut*. Mar 2023;72(3):581-589. doi:10.1136/gutjnl-2022-327777
2. de Franchis R, Bosch J, Garcia-Tsao G, Reiberger T, Ripoll C, Baveno VIIF. Baveno VII - Renewing consensus in portal hypertension. *J Hepatol*. Apr 2022;76(4):959-974. doi:10.1016/j.jhep.2021.12.022
3. Harrison SA, Abdelmalek MF, Caldwell S, et al. Simtuzumab Is Ineffective for Patients With Bridging Fibrosis or Compensated Cirrhosis Caused by Nonalcoholic Steatohepatitis. *Gastroenterology*. Oct 2018;155(4):1140-1153. doi:10.1053/j.gastro.2018.07.006
4. Sanyal AJ, Harrison SA, Ratziu V, et al. The Natural History of Advanced Fibrosis Due to Nonalcoholic Steatohepatitis: Data From the Simtuzumab Trials. *Hepatology*. Dec 2019;70(6):1913-1927. doi:10.1002/hep.30664
5. Harrison SA, Wong VW, Okanoue T, et al. Selonsertib for patients with bridging fibrosis or compensated cirrhosis due to NASH: Results from randomized phase III STELLAR trials. *J Hepatol*. Jul 2020;73(1):26-39. doi:10.1016/j.jhep.2020.02.027
6. Younossi ZM, Anstee QM, Wai-Sun Wong V, et al. The Association of Histologic and Noninvasive Tests With Adverse Clinical and Patient-Reported Outcomes in Patients With Advanced Fibrosis Due to Nonalcoholic Steatohepatitis. *Gastroenterology*. Apr 2021;160(5):1608-1619 e13. doi:10.1053/j.gastro.2020.12.003
7. Sanyal AJ, Anstee QM, Trauner M, et al. Cirrhosis regression is associated with improved clinical outcomes in patients with nonalcoholic steatohepatitis. *Hepatology*. May 2022;75(5):1235-1246. doi:10.1002/hep.32204
8. Chalasani N, Abdelmalek MF, Garcia-Tsao G, et al. Effects of Belapectin, an Inhibitor of Galectin-3, in Patients With Nonalcoholic Steatohepatitis With Cirrhosis and Portal Hypertension. *Gastroenterology*. Apr 2020;158(5):1334-1345 e5. doi:10.1053/j.gastro.2019.11.296
9. Are VS, Vuppalanchi R, Vilar-Gomez E, Chalasani N. Enhanced Liver Fibrosis Score Can Be Used to Predict Liver-Related Events in Patients With Nonalcoholic Steatohepatitis and Compensated Cirrhosis. *Clin Gastroenterol Hepatol*. Jun 2021;19(6):1292-1293 e3. doi:10.1016/j.cgh.2020.06.070
10. Loomba R, Noureddin M, Kowdley KV, et al. Combination Therapies Including Cilofexor and Firsocostat for Bridging Fibrosis and Cirrhosis Attributable to NASH. *Hepatology*. Feb 2021;73(2):625-643. doi:10.1002/hep.31622
